# M^6^A transcriptome-wide map of circRNAs identified in the testis of normal and AZ-treated *Xenopus laevis*

**DOI:** 10.1186/s41021-023-00279-0

**Published:** 2023-09-01

**Authors:** Xin Zhang, Linlin Sai, Weiliang Zhang, Xingzheng Kan, Qiang Jia, Cunxiang Bo, Wenhui Yin, Hua Shao, Mingming Han, Cheng Peng

**Affiliations:** 1 Shandong Academy of Occupational Health and Occupational Medicine, Shandong First Medical University & Shandong Academy of Medical Sciences, Ji’nan, Shandong China; 2 Shandong First Medical University & Shandong Academy of Medical Sciences, Shandong Academy of Occupational Health and Occupational Medicine, Ji’nan, Shandong China; 3 Eusyn Institute of Health Science, Brisbane, QLD 4102 Australia

**Keywords:** RNA methylation, M^6^A, CircRNA, Amphibious, Atrazine

## Abstract

**Background:**

Evidence showed that N^6^-methyladenosine (m^6^A) is strongly associated with male germline development. However, the role of m^6^A methylation on circRNAs in amphibians remains unknown. In this study, we conducted m^6^A sequencing analysis to explore the m^6^A transcriptome-wide profile of circRNAs in testis tissues of *Xenopus laevis* (*X. laevis*) with and without treatment with 100 µg/L atrazine (AZ).

**Results:**

The analysis showed that m^6^A modification of circRNAs enriched in sense overlapping in testes of *X. laevis*. We identified the differential m^6^A modification sites within circRNAs in testes of AZ-exposed *X. laevis* and compared that with animals from control group. The results showed that a total of 1507 methylated m^6^A sites was induced by AZ (760 up-methylated and 747 down-methylated). The cross-analysis exhibited a negative correlation of differentially methylated m^6^A peaks and circRNAs expression level. The Kyoto Encyclopedia of Genes and Genomes (KEGG) analysis indicated that 20 key pathways may be involved in the mechanism of testis damage of AZ-exposed *X. laevis*.

**Conclusions:**

These findings indicated that differentially m^6^A-methylated circRNAs may play important roles in abnormal testis development of AZ-exposed *X. laevis*. This study is the first report about a map of m^6^A modification of circRNAs in male *X. laevis* and provides a basis for further studying on the function and mechanism of m^6^A methylation of circRNAs in the testis development of amphibian.

**Supplementary Information:**

The online version contains supplementary material available at 10.1186/s41021-023-00279-0.

## Introduction

N^6^-methyladenosine (m^6^A), as the most widespread post-transcriptional RNA modification, has attracted extensive attention in the field of epigenetics [1]. Emerging evidence suggested that m^6^A modifications played an important role in RNA metabolism, and its dynamic regulation was significantly correlated with gene expression [2]. Additionally, a previous study reported that knockout of RNA m^6^A regulators in the testis led to abnormal metabolism of the RNAs, which eventually caused spermatogenetic disorders and infertility [3]. Recently, it is reported that the process of spermatogenesis was influenced by gene regulation at transcriptional, post-transcriptional and epigenetic levels [4]. RNA m^6^A modification played an important role in spermatogenesis [5].

Circular RNAs (circRNAs) are a class of non-coding RNA with the length more than 200 nucleotides [6]. Its closed-loop structures lacking a 5’-cap structure and a 3’-poly (a) tail, were generated by back-splicing of linear mRNAs [7]. They were abundant, stable and highly conserved in evolution [8]. CircRNAs have pivotally important role in regulating gene expression by sequestering specific miRNAs like a sponge or buffering inhibition of mRNA targets [9]. At present, m^6^A modification has been identified in circRNAs, and most m^6^A circRNAs are expressed in a cell type-specific manner suggesting that m^6^A circRNAs play different biological functions in specific cell types [10]. For example, Xu et al. found that METTL3/FTO/YTHDF1/2-mediated m^6^A modification can enhance the stability of circRNA-SORE and induce sorafenib resistance in hepatocellular carcinoma (HCC) [11]. METTL3-mediated m^6^A methylation regulates the expression of circMETTL3 and promotes breast cancer cell proliferation and migration [12]. M^6^A modification improves the expression of circCUX1 and makes hypopharyngeal squamous cell carcinoma radioresistant [13]. Mettl14-mediated m^6^A methylation could also export circGFRa1 to cytoplasm from nucleus and promote female germline stem cells (FGSCs) self-renewal [14]. Tang et al. found that m^6^A modification modulates the biogenesis of a subset of circRNAs in male germ cells and further regulates spermatogenesis [15]. To our knowledge, the m^6^A transcriptome-wide map of circRNAs in amphibians remains to be explored.

Studies have found that environmental pollution could negatively affect amphibians’ health status and fertility, ultimately leading to a significant decrease in their population [16–18]. Atrazine (2-chloro-4-ethylamino-6-isopropylamino-s-triazine, AZ), an endocrine disrupting chemical (EDCs), is a highly effective triazine herbicide widely used in China, the USA and other countries [19]. It is often detected in groundwater, surface and drinking water [20]. It has been reported that AZ could cause metamorphosis of tadpoles and gonadal dysplasia of in several frog species [21–23], such as demasculinization and complete feminization of *X. laevis* [21]. This may be one of the factors causing global amphibian declines [24, 25]. In our previous study, we examined the damage of *X. laevis* exposed to AZ (0.1, 10, or 100 µg/L) for 90 days in the water environment. The results showed that AZ caused the decrease of gonad weight and gonad somatic index and the histological damage of testis in *X. laevis* [26]. In addition, we reported that m^6^A transcriptome-wide map of an amphibian species *X. laevis*. These findings provided clues to reveal the role of m^6^A which may affect amphibian testis development [27]. However, the mechanism of reproductive toxicity in male *X. laevis* chronically exposed to AZ remains unclear. Therefore, it is necessary to investigate the potential changes of m^6^A modification of circRNAs.

Thus, this study aimed to address whether changes in m^6^A modification of circRNAs are associated with abnormal testicular development in *X. laevis* chronically exposed AZ. We comprehensively analyzed the m^6^A modification profiles of circRNAs in normal and 100 µg/L AZ-exposed *X. laevis* for 180 days, respectively. Then, we predicted the signal pathways in which dysregulated m^6^A methylation of circRNAs involved by KEGG pathway analysis. Meanwhile, our work will provide a basis for further studying on the function and mechanism of m^6^A methylation of circRNAs in the abnormal testis development of *X. laevis* and also provide novel insights into its function and biological significance in amphibians.

## Methods

### Ethic approval

All animal experiments were carried out according to relevant guidelines and care regulations. All procedures complied with the “Principles of Animal Care”. The protocol was assessed and approved by the Committee on the Ethics of Animal Experiments of Shandong Academy of Occupational Health and Occupational Medicine [28].

### Animal treatment

Three pairs of adult male and female *X. laevis* were purchased from the Chinese Academy of Sciences (Beijing, China). The offspring were generated by natural mating. Laboratory freshwater produced by UV treatment and carbon filtration was used for the acclimatization of frogs in the laboratory and for all subsequent exposures. The *X. laevis* were kept at an average water temperature of 22 ± 2 °C at pH 7.5, under 12 h light and 12 h dark cycle. Tadpoles were fed fairy shrimp (*Artemia nauplii*) eggs in a young age daily and pork liver three times per week *ad libitum* when the tadpoles completed metamorphosis [26].

At Nieuwkoop-Faber (NF) stage 47 (13 d post-hatch), mixed sex tadpoles from the same parents were discretionarily divided into two groups. One group was exposed to AZ at a dose of 100 µg/L (dissolved in solvent vehicle DMSO (0.01%)) for 180 days, and the other group was exposed to 0.01% DMSO only. Test solutions were renewed by 50% replacement every 48 h. The animals were monitored daily for health status and morphological changes. All of the females were excluded in this study. On day 180, the *X. laevis* in the control and AZ-exposed groups were sacrificed, respectively. The testes were isolated and weighed, and finally stored at − 80 °C for further analysis [8, 27].

### CircRNAs preparation

Each group was recommended to include at least three biological replicates [29]. We randomly selected six testes (three from controls and three from 100 µg/L AZ-exposed groups) for circRNAs analysis. Total RNA from testes was extracted using Trizol reagent (Invitrogen Corporation, CA, USA) according to the manufacturer’s instruction. The concentration and purity of RNAs were measured using a NanoDrop® ND-2000 spectrophotometer (Thermo, Waltham, MA, USA). RNA integrity was examined by denaturing gel electrophoresis experiments [8]. RNA samples were further purified and converted to double-stranded cDNA for microarray analysis according to the Agilent ® *Xenopus* 4 × 44 k Gene Expression Microarray protocols.

### CircRNAs m^6^A MeRIP sequencing

M^6^A of circRNAs were sequenced by MeRIP sequencing using Illumina HiSeq sequencer. In short, the fragmented RNA was incubated with anti-m^6^A polyclonal antibody (Synaptic Systems, Goettingen, Germany, 202,003) in IPP buffer for 2 h at 4 °C. Under the same conditions, the mixture was immune precipitated by incubation with protein-A beads (Thermo Fisher). Then, bound RNA was eluted and then extracted according to the manufacturer’s instructions. Purified RNA was used for RNA-seq library generation with NEBNext® Ultra™ RNA Library Prep Kit (NEB). Input samples without immune precipitation and m^6^A IP samples were subjected to 150 bp paired-end sequencing on Illumina HiSeq sequencer. Paired-end reads were obtained, and quality control was performed by Q30. More specific operations have been elucidated in our previous study [26, 27].

### Data analysis

After sequencing, reads were trimmed with 3’ adaptor and low-quality readings were removed to produce clean reads with Cutadapt software (v1.9.3). Clean reads from all libraries were aligned to genome by bowtie 2 software [30]. Then circRNAs were detected and identified with find circ software [31], the circBase database [32] was also used to annotate the identified circRNAs, clean reads of all libraries were mapped to genome using hisat2 software (v2.04) [33]. Methylated sites on circRNAs were detected by MACS software [34], and differentially methylated sites of circRNAs were identified by diffReps [35]. In addition, the overlapping sites between the peaks, identified by both software based on the circRNAs exons were chosen for further analyzed. Pathway enrichment analysis of differentially methylated protein coding genes was performed through KEGG pathway database.

## Results

### AZ induced physiological changes of *X. laevis*

Compared with froglets in the control, the mortality of froglets significantly increased, the weight and gonadosomatic index (GSI) of the testis were significantly reduced, the metamorphosis time of tadpole was prolonged in AZ-exposed *X. laevis*. Histopathological results showed large empty spaces and irregular shape of seminiferous lobules in the testicular tissue. The number of germ cells in testicular tissue was significantly reduced. These results have been reported in our previous paper [26].

### M^6^A sites within circRNAs in the testes of control and AZ-exposed *X. laevis*

**Fig. 1:**
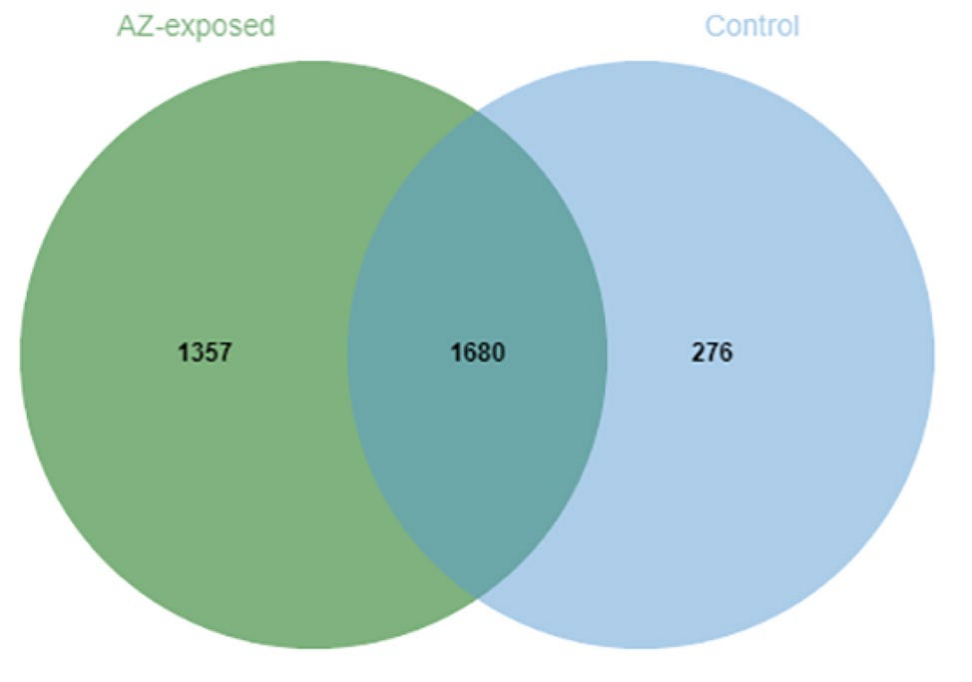
Venn diagram showing the overlap of m^6^A peaks within circRNAs in the testes of control and AZ-exposed *X. laevis*

The m^6^A transcriptome-wide profile of circRNAs were performed in testes in three biological replicates from the controls (n = 3) and AZ-exposed *X. laevis* (n = 3). We found that there were 1680 m^6^A peaks were shared in controls and AZ-exposed *X. laevis*, while 276 m^6^A peaks were identified in controls but absent in AZ-exposed *X. laevis*, and 1357 m^6^A peaks were identified in AZ-exposed *X. laevis* but absent in controls (Fig. 1).

### Distribution of m^6^ A-methylated circRNAs in the testes of control and AZ-exposed *X. laevis*

**Fig. 2:**
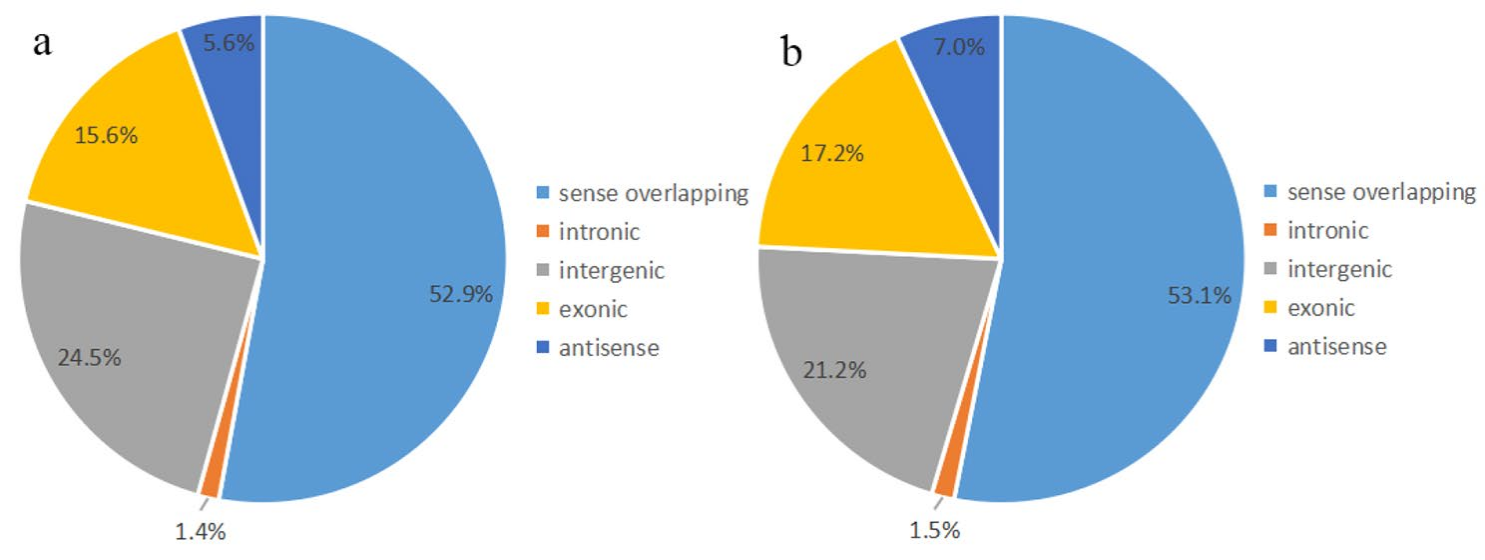
Distribution of m^6^A-methylated circRNAs in the testes of control and AZ-exposed *X. laevis* a: Pie charts showing the percentage of the distribution positions of m^6^A-methylated circRNAs in control group. b: Pie charts showing the percentage of the distribution positions of m^6^A-methylated circRNAs in AZ-exposed group

To further illustrate the distribution profiles of m^6^A-methylated circRNAs, we analyzed the genomic position of total m^6^A-methylated circRNAs in the testes of control and AZ-exposed *X. laevis*, respectively. We found that m^6^A-methylated circRNAs in both groups were divided into 5 groups: antisense, exonic, intergenic, intronic, sense overlapping. Particularly, we found that the most of m^6^A-methylated circRNAs were concentrated in sense overlapping (52.9% in control and 53.1% in AZ-exposed groups) (Fig. 2a and b).

### Differentially m^6^ A modification sites of circRNAs in *X. laevis* exposed to 100 µg/L AZ

**Fig. 3:**
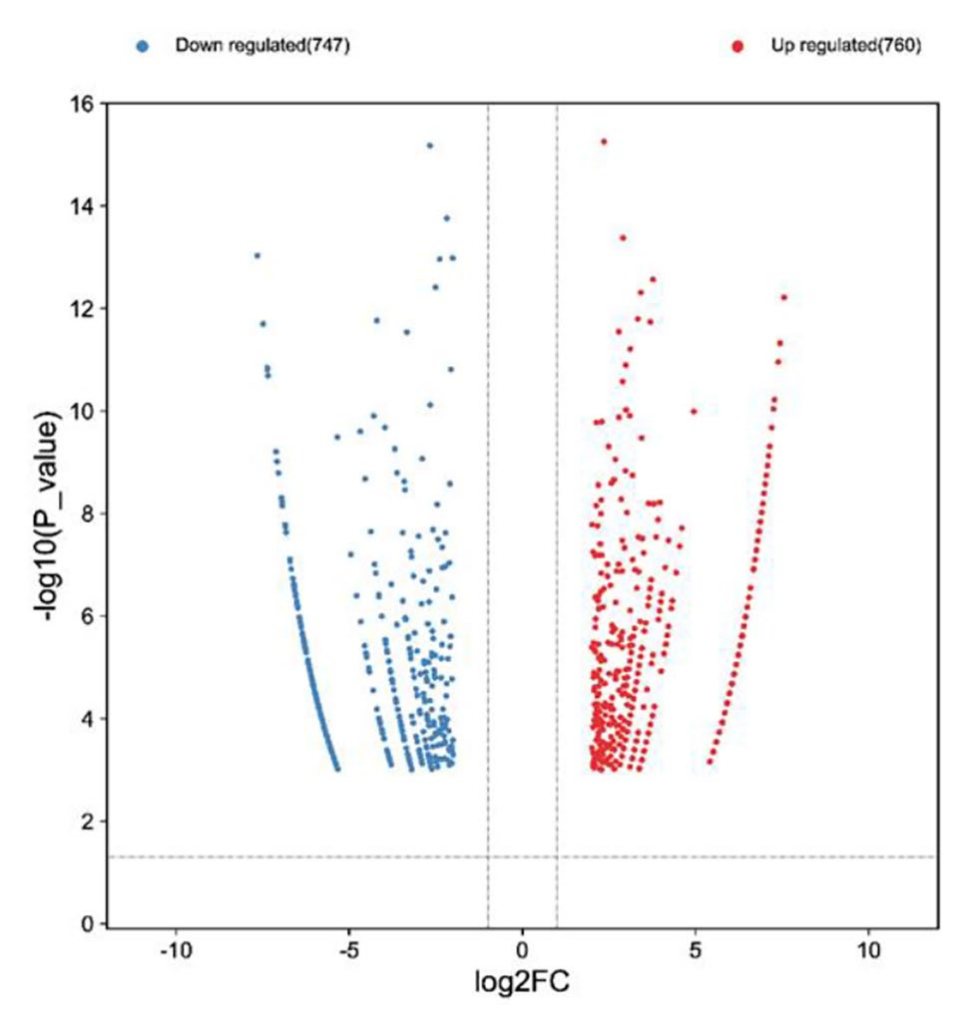
Volcano plots showing –log10 (*P*_value) versus log2FC in m^6^A methylated sites on circRNAs in the testes of control and AZ-exposed *X. laevis*. Red circles denote significantly up-regulated m^6^A methylated sites, whereas blue circles denote significantly down-regulated m^6^A methylated sites (*p* < 0.05 and fold change ≥ 4)

**Fig. 4:**
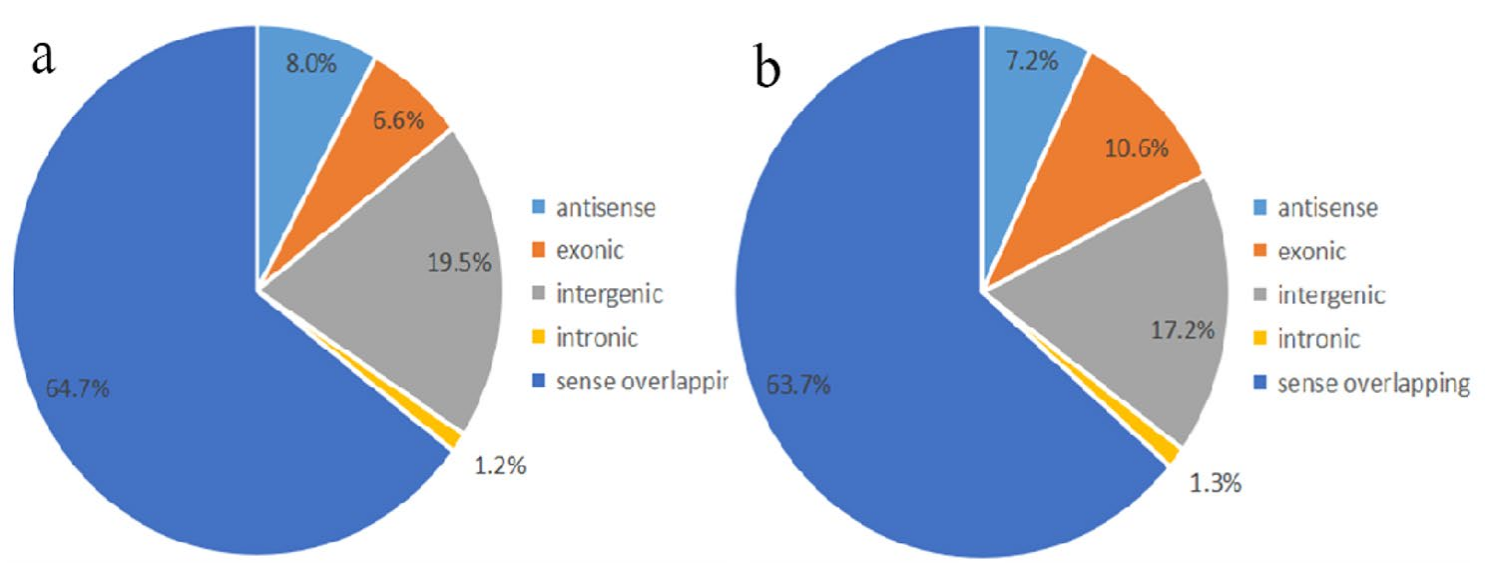
Overview the distribution of differentially m^6^A modification sites of circRNAs. **a**: Pie charts showing the percentage of up-methylated m^6^A in five segments. **b**: Pie charts showing the percentage of down-methylated m^6^A peaks in five segments

There were 1507 differentially methylated m^6^A sites were identified. Among them, 760 m^6^A methylated sites were significantly up-regulated, 747 m^6^A methylated sites were significantly down-regulated (Table S1 and Fig. 3). Tables 1 and 2 showed that top ten up-and down-methylated m^6^A sites of circRNAs with the highest fold change (FC) values. To further illustrate the distribution profiles of differentially methylated circRNAs, we found most significantly methylated circRNAs belonged to sense overlapping (Fig. 4a and b).

**Table 1:** The top 10 up-methylated m^6^A sites of circRNAs

chrom	txStart	txEnd	circRNA	Foldchange	Regulation
NC_030725.1	138,173,201	138,173,760	NC_030725.1:138171762–138,186,798+	1047.5	up
NC_030730.1	60,836,021	60,836,560	NC_030730.1:60770414–60,848,459+	544.8	up
NC_030741.1	38,394,381	38,394,940	NC_030741.1:38391889–38,396,199+	428.8	up
NC_030734.1	51,326,541	51,327,120	NC_030734.1:51319661-51329727-	188	up
NC_030729.1	14,574,441	14,575,060	NC_030729.1:14566445-14575976-	187.9	up
NC_030741.1	94,059,881	94,060,280	NC_030741.1:94040702-94069184-	173.4	up
NC_030732.1	110,818,421	110,818,980	NC_030732.1:110818083-110819953-	167.4	up
NC_030732.1	59,329,921	59,330,840	NC_030732.1:59317677–59,337,960+	155.5	up
NC_030738.1	116,486,141	116,487,120	NC_030738.1:116446394-116502224-	152.5	up
NC_030741.1	20,961,581	20,962,140	NC_030741.1:20954726–20,967,910+	146.6	up

txStart/txEnd: Start/end position of the differentially methylated RNA sites

**Table 2:** The top 10 down-methylated m^6^A sites of circRNAs

chrom	txStart	txEnd	circRNA	Foldchange	Regulation
NC_030741.1	26,736,741	26,737,300	NC_030741.1:26724629–26,775,531+	3995.5	down
NC_030726.1	38,143,141	38,143,700	NC_030726.1:38131187-38150843-	201.3	down
NC_030737.1	3,714,221	3,714,760	NC_030737.1:3707843-3738590-	179.5	down
NC_030728.1	135,118,701	135,119,340	NC_030728.1:135115660–135,120,836+	165.5	down
NC_030724.1	83,840,521	83,841,540	NC_030724.1:83828634-83844198-	165	down
NC_030738.1	83,071,141	83,071,680	NC_030738.1:83069676–83,126,138+	163.1	down
NC_030738.1	11,481,761	11,482,340	NC_030738.1:11462943–11,533,553+	150.7	down
NC_030739.1	19,273,999	19,274,106	NC_030739.1:19272571–19,278,583+	138.9	down
NC_030724.1	152,774,701	152,776,080	NC_030724.1:152754234–152,779,042+	135.9	down
NC_030731.1	115,186,221	115,187,200	NC_030731.1:115185766–115,230,884+	132.3	down

txStart/txEnd: Start/end position of the differentially methylated RNA sites

### Conjoint analysis of m^6^ A-Seq and RNA-Seq Data of control and AZ-exposed *X. laevis*

**Fig. 5:**
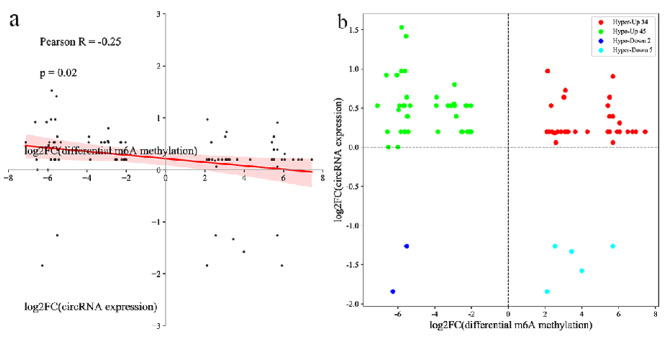
**a**: Dot plot of log2FC (circRNAs expression) against log2FC (differential m^6^A methylation) showing a negative correlation between overall m^6^A methylation and mRNA expression level (*P* = 0.02; Pearson R = -0.25). **b**: Four quadrant plots showing gene expression with differentially methylated m^6^A peaks

To elucidate the potential relationship between m^6^A methylation and gene expression, we performed a cross-analysis of m^6^A-seq and RNA-seq data. As shown in Table S2, in the case of combined analysis of differentially methylated m^6^A sites and differentially expressed circRNAs, circRNA“NC_030733.1:68560883-68632668-”,“NC_030737.1:31865134–31952348”, “NC_030737.1:83574596–83583489+”,“NC_030733.1:34483227-34511810-”,“NC_030733.1:68560883-68632668-”, “NC_030737.1:31865134-31952348-” were down-regulated in the testis of AZ-exposed *X. laevis*, but the m^6^A modification of which were up-regulated. However, circRNA “NC_030738.1:21126033–21131815+” was up-regulated in the testis of AZ-treated *X. laevis*, but the m^6^A modification of which was down-regulated. The result showed that differentially methylated m^6^A modification may negatively regulated the expression of circRNAs. Additionally, in the case of combined analysis of differentially methylated m^6^A sites and total circRNAs, we discovered a negative correlation of differentially methylated m^6^A peaks and circRNAs expression level (*P* = 0.02; Pearson R = -0.25) (Fig. 5a). The results of the four-quadrant diagram analysis showed that 760 hyper-methylated m^6^A sites were positive correlated with 34 up-regulated circRNAs transcripts, namely ‘hyper-up’, but 5 genes were found with down-regulated circRNAs transcripts, namely ‘hyper-down’. In the contrast, in 747 hypo-methylated m^6^A sites, we found 45 genes with up-regulated circRNAs transcripts and 2 genes with down-regulated circRNAs transcripts, named ‘hypo-up’ and ‘hypo-down’, respectively (Fig. 5b). Notably, we found that 57.0% (45/79) of the up-regulated circRNAs transcripts were associated with hypo-methylated m^6^A peaks. Meanwhile, the numbers of ‘hyper-down’ and ‘hypo-up’ genes were more than those of ‘hyper-up’ and ‘hypo-down’ genes. It indicated that m^6^A modifications may tend to be negatively correlated with circRNAs expression in the testes of *X. laevis* exposed to 100 µg/L AZ.

### The enrichment pathways of genes related differentially m^6^A-methylated circRNAs by KEGG

**Fig. 6:**
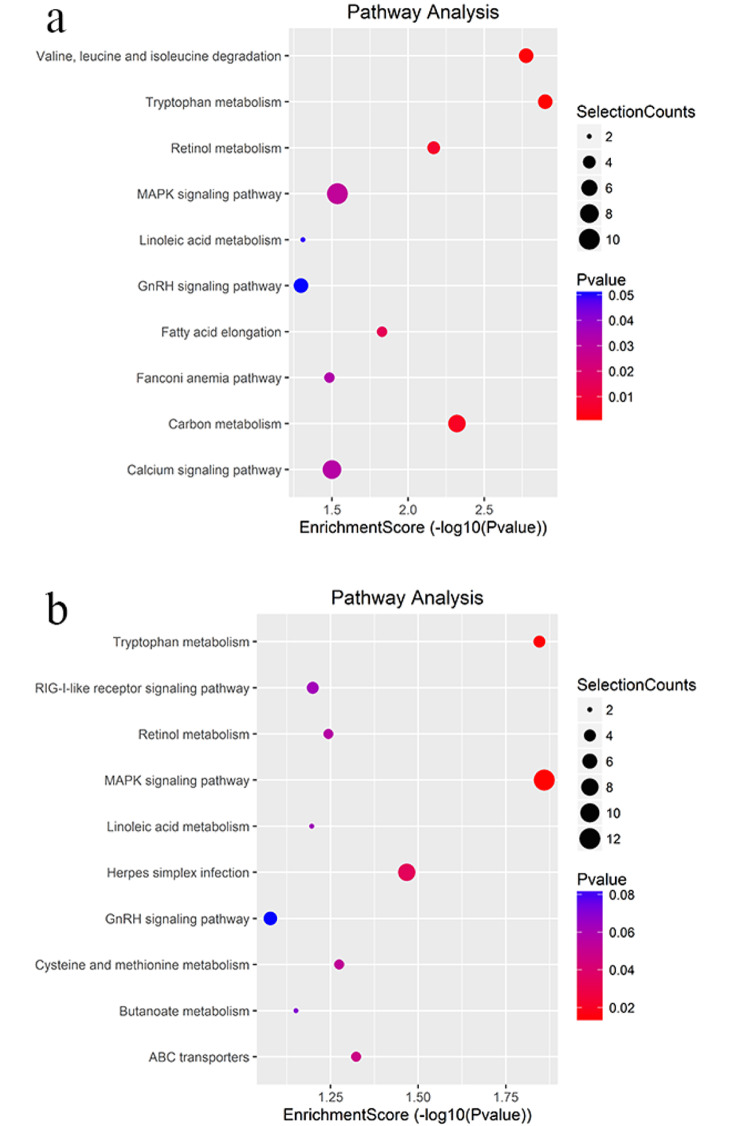
The annotated significant pathways targeted by the enrichment score of the differentially m^6^A-methylated circRNAs-related genes including up-methylated (**a**) and down-methylated (**b**) in testis of *X. laevis* exposed to 100 µg/L AZ. The horizontal axis is the -log10 (*P*-value) for the pathway and the vertical axis is the pathway category

To explore the effect of differential m^6^A-methylated circRNAs in testis of AZ-exposed *X. laevis*. KEGG pathway analysis were performed for target genes related to the modified circRNAs. The result revealed that target genes related to up-methylated circRNAs were mainly enriched in ten pathways, such as Valine, Leucine and isoleucine degradation, Tryptophan metabolism, Retinol metabolism, MAPK signaling pathway, Linoleic acid metabolism, GnRH signaling pathway, Fatty acid elongation, Fanconi anemia pathway, Carbon metabolism and Calcium signaling pathway (Fig. 6a). Meanwhile, target genes related to down-methylated circRNAs were significantly involved in Tryptophan metabolism, RIG-I-Like receptor signaling pathway, Retinol metabolism, MAPK signaling pathway, Linoleic acid metabolism, Herpes simplex infection, GnRH signaling pathway, Cysteine and methionine metabolism, Butanoate metabolism and ABC transporters (Fig. 6b).

## Discussion

Studies have shown that AZ could cause histological damage of the testis, reduced sperm count and generate abnormal sperm [36]. In addition, *X. laevis* exposed to AZ also showed damaged germ cell and reduced testicular weight [37]. These results were consistent with the previous results of our study [26].

M^6^A methylation has been shown to be one of the most abundant internal modifications of RNA in higher eukaryotes [38, 39]. It was characterized by dynamic reversibility, wide spready and unique patterns [10]. Emerging evidence have suggested that m^6^A methylation played important biological functions in RNA modification, tumorigenesis, fat metabolism, reproductive development, and so on [39, 40]. And the role of m^6^A circRNAs has also been gradually elucidated. Hence, we explored m^6^A transcriptome-wide map of circRNAs identified in the testis of normal and AZ-treated *X. laevis*, to predict the role of m^6^A modification of circRNAs in abnormal testicular development. In this study, we investigated the changes of m^6^A modification of circRNAs in the testis of *X. laevis* by exogenous environmental stimulation. Our findings showed that the number of m^6^A sites within circRNAs increased in the testis of AZ-exposed *X. laevis* compared with that of controls. The majority of m^6^A methylated circRNAs and differential m^6^A methylated ones enriched in sense overlapping. Interestingly, studies have found that the back splicing tends to occur predominantly in m^6^A-enriched sites in male germ cells. The change allows the most circRNAs contain large open reading frames to ensure long-term and stable protein production for specific physiological processes in the absence of corresponding linear mRNA [15, 41]. And aberrant m^6^A methylation may affect the abnormal spermatogenesis [42]. Therefore, we speculated that m^6^A modification enriched in sense overlapping may play an important role in abnormal development of testis in amphibian species.

To further explore the potential role of m^6^A modification in the testes of AZ-exposed *X. laevis*, we performed combined analysis of m^6^A-Seq and RNA-Seq. Results revealed that a negative correlation between differentially methylated peaks and circRNAs expression levels. It is reported that circRNAs were characterized by covalently closed structure and resistance to exonucleases, but m^6^A modification could induce the degradation process of circRNAs by regulating their stability [29]. Park et al. found that m^6^A-methylated circRNAs were cleaved by endoribonucleases via a YTHDF2-HRSP12-RNase P/MRP axis [43]. This may affect the biological function of circRNAs [44], such as m^6^A modification of circNSUN2 could promote the liver metastasis of colorectal cancer by forming a circNSUN2/IGF2BP2/HMGA2 RNA-protein ternary complex [12]. Additionally, it has been reported that the specific role of m^6^A modification on gene expression largely depended on the type of m^6^A ‘readers’ [45]. Hence, we speculated that m^6^A modification in circRNAs may play a potential role in the abnormal testicular development of AZ-exposed *X. laevis*. In the present study, we only examined circRNAs expression, more functional experiments will be needed to further verify the regulation role of m^6^A modification on gene expression.

We further analyzed the role of differentially m^6^A-methylated circRNAs related target genes in AZ-treated *X. laevis* for which 20 key pathways were obtained by KEGG pathway annotation method. In this study, the most GeneRatio term was “MAPK signaling pathway”. Studies have shown that some exogenous stimulants could activate MAPK signaling pathway, affected Sertoli cells (SCs) proliferation and blood-testis barrier (BTB) structure [46, 47]. Interestingly, In the process of spermatogenesis, SCs not only provided mechanical and nutritional support [48, 49], but also protected germ cells by forming an immune protective environment through the BTB [50, 51]. Therefore, we assumed that MAPK signaling pathway regulated by circRNAs with m^6^A modifications might mediate the abnormal testis development of AZ-exposed *X. laevis.*

“GnRH signaling pathway” was also included in the result of KEGG. GnRH, as a local bioregulator, controlled the secretion of gonadotropin of the pituitary gland, including follicle stimulating hormone and luteinizing hormone, which were critical components affecting sperm development [52]. In addition, it was reported that GnRH could also be synthesized in seminiferous tubules, and as a paracrine mediator of spermatogenesis [53]. In GnRH deficient males, there would be incomplete testicular growth and maturation, cryptorchidism, androgen deficiency and other symptoms [54]. Therefore, m^6^A-methylation of circRNAs involved in “GnRH signaling pathway” may play an important role in the growing development and functional activity of testis of AZ-exposed *X. laevis*.

In addition, it has been shown that Ca^2+^, which was produced by the Calcium Signaling Pathway, could directly control metabolism, secretion, fertilization, proliferation and other processes [55]. And it also participated in the cAMP-induced steroidogenesis in Leydig cells [56]. Therefore, we speculated that “Calcium Signaling Pathway” regulated by m^6^A-methylated circRNAs may involve testicular dysplasia of AZ-exposed *X. laevis*. Most of these pathways in the result were related to metabolism such as “Linoleic acid metabolism” and “Fatty acid elongation” signaling pathways. Fatty acid (FA) mainly included monounsaturated fatty acids (MUFA) and polyunsaturated fatty acids (PUFA) [57], while Linoleic acid were essential PUFA [58]. Its synthesis was mediated by rate-limiting elongation and desaturation enzymes, which were regulated by fatty acid desaturase 2 (FADS2) and elongation of very-long-chain fatty acids-like 2 (ELOVL2) [58]. It has been found that arrested spermatogenesis and lacked mature spermatozoa in the testis of mice with FADS2 and ELOVL2 gene knockout [59, 60]. Additionally, it was reported that the FA metabolic disorders could occur in infertile men [57]. Hence, the role of m^6^A-methylated circRNAs involved in “Linoleic acid metabolism” and “Fatty acid elongation” signaling pathways deserve further study in male reproductive damage. Consequently, the results predicted that m^6^A methylation of circRNAs may regulate the differential expression of these target genes in abnormal testis development of *X. laevis* exposed to 100 µg/L AZ.

## Conclusion

We detected the m^6^A transcriptome-wide profile of circRNAs in the testes of control and AZ-exposed *X. laevis.* The results showed that AZ could alter expression profile in 1507 m^6^A methylated peaks within circRNAs in which 760 were significantly up-methylated and 747 significantly down-methylated, and they mainly enriched in sense overlapping. Conjoint analysis indicated that a negative correlation of differentially methylated m^6^A peaks and circRNAs expression level, suggesting a regulatory role of m^6^A modification of circRNAs in amphibious gene expression. KEGG pathway analysis revealed that m^6^A related to “MAPK signaling pathway”, “GnRH signaling pathway”, “Calcium signaling pathway” and pathways related to metabolism such as “Linoleic acid metabolism” and “Fatty acid elongation” maybe play a pivotal role in the abnormal testis development of AZ-exposed *X. laevis*. Our study provided a basis for further studies on the function and mechanism of m^6^A methylation of circRNAs in the abnormal testis development of *X. laevis*. Meanwhile, this study presented the first m^6^A transcriptome-wide map of circRNAs in amphibian species *X. laevis.* This may help to further understand the role of m^6^A methylation in testis development and spermatogenesis in amphibians.

### Electronic supplementary material

Below is the link to the electronic supplementary material.

Supplementary Material 1

## Data Availability

All data generated and analyzed during this study are included in this published article and its supplementary information files.

## Abbreviations

M^6^A: N6-methyladenosine
*X. laevis*: *Xenopus laevis*
AZ: Atrazine
circRNAs: Circular RNAs
EDCs: Endocrine disrupting chemicals
FC: Fold change
HCC: Hepatocellular carcinoma
FGSCs: Female germline stem cells
GSI: Gonadosomatic index
KEGG: Kyoto Encyclopedia of Genes and Genomes
MAPK: Mitogen-activated protein kinases
SCs: Sertoli cells
BTB: Blood-testis barrier
GnRH: Gonadotropin-releasing hormone
FA: Fatty acid
MUFA: Monounsaturated fatty acids
PUFA: Polyunsaturated fatty acids
FADS2: Fatty acid desaturase 2
ELOVL2: Elongation of very-long-chain fatty acids-like 2
