# Supplementary material for: M6A transcriptome-wide map of circRNAs identified in the testis of normal and AZ-treated Xenopus laevis
Source: Genes Environ. 2023 Sep 1;45:23. doi: 10.1186/s41021-023-00279-0 (PMC10472591; doi:10.1186/s41021-023-00279-0)
Supplement: Supplementary file 1 — Supplementary Material 1 [file 41021_2023_279_MOESM1_ESM.docx]

**Table S1** The up and down methylated peaks

| **chrom** | **txStart** | **txEnd** | **Regulation** | **circRNA** | **Foldchange** |
| --- | --- | --- | --- | --- | --- |
| NC_030728.1 | 142565601 | 142566100 | up | NC_030728.1:142552653-142570230- | 105 |
| NC_030733.1 | 68600261 | 68600820 | up | NC_030733.1:68560883-68632668- | 60.4 |
| NC_030737.1 | 66983861 | 66984160 | up | NC_030737.1:66982801-67012866- | 45.6 |
| NC_030738.1 | 45705021 | 45705520 | up | NC_030738.1:45688773-45717364- | 48.4 |
| NC_030740.1 | 16602835 | 16602940 | up | NC_030740.1:16602836-16603614+ | 6.006451613 |
| NC_030738.1 | 331621 | 332000 | up | NC_030738.1:261425-337048+ | 116.9 |
| NC_030728.1 | 96236241 | 96236780 | up | NC_030728.1:96198200-96268469+ | 10.39655172 |
| NC_030734.1 | 129412281 | 129412820 | up | NC_030734.1:129407662-129481427- | 42.6 |
| NC_030732.1 | 50708481 | 50708880 | up | NC_030732.1:50672751-50716322+ | 42.6 |
| NC_030727.1 | 108102961 | 108103440 | up | NC_030727.1:108093966-108109905+ | 60.4 |
| NC_030737.1 | 31937501 | 31938020 | up | NC_030737.1:31865134-31952348- | 5.819354839 |
| NC_030732.1 | 80751361 | 80751860 | up | NC_030732.1:80724881-80768348+ | 4.261127596 |
| NC_030732.1 | 45320881 | 45321140 | up | NC_030732.1:45316337-45330198+ | 45.6 |
| NC_030727.1 | 90767501 | 90768420 | up | NC_030727.1:90727016-90785894+ | 11.73831776 |
| NC_030725.1 | 138173201 | 138173760 | up | NC_030725.1:138171762-138186798+ | 1047.5 |
| NC_030730.1 | 27368901 | 27369460 | up | NC_030730.1:27335277-27385854- | 42.5 |
| NC_030738.1 | 14969641 | 14970260 | up | NC_030738.1:14947742-14973216- | 51.5 |
| NC_030736.1 | 5226021 | 5226520 | up | NC_030736.1:5183270-5245593+ | 54.4 |
| NC_030732.1 | 156063381 | 156063960 | up | NC_030732.1:156022969-156066499+ | 8.420560748 |
| NC_030733.1 | 83723381 | 83723760 | up | NC_030733.1:83698731-83725586- | 69.4 |
| NC_030738.1 | 46938041 | 46938880 | up | NC_030738.1:46917518-46954801+ | 4.217213115 |
| NC_030728.1 | 7691881 | 7692260 | up | NC_030728.1:7628695-7711913- | 8.553333333 |
| NC_030734.1 | 49682341 | 49682660 | up | NC_030734.1:49655887-49688000- | 10.544 |
| NC_030732.1 | 113130941 | 113131500 | up | NC_030732.1:113119012-113147396- | 7.432 |
| NC_030738.1 | 94414021 | 94414520 | up | NC_030738.1:94396745-94415826+ | 84.2 |
| NC_030741.1 | 32240961 | 32241077 | up | NC_030741.1:32231106-32241077+ | 10.4137931 |
| NC_030736.1 | 5182036 | 5182380 | up | NC_030736.1:5182037-5223060- | 48.5 |
| NC_030736.1 | 59008121 | 59008320 | up | NC_030736.1:59005035-59035694- | 6.264 |
| NC_030738.1 | 32809261 | 32809520 | up | NC_030738.1:32804454-32826943- | 48.5 |
| NC_030725.1 | 12365741 | 12365767 | up | NC_030725.1:12358541-12365767- | 48.5 |
| NC_030735.1 | 119639301 | 119639920 | up | NC_030735.1:119603296-119686801- | 4.483333333 |
| NC_030741.1 | 66840381 | 66841020 | up | NC_030741.1:66837870-66841460+ | 48.5 |
| NC_030733.1 | 119419741 | 119420280 | up | NC_030733.1:119396840-119430773+ | 10.08411215 |
| NC_030733.1 | 51890021 | 51890480 | up | NC_030733.1:51836941-51895818- | 57.3 |
| NC_030726.1 | 87776421 | 87776900 | up | NC_030726.1:87772700-87777018+ | 42.6 |
| NC_030734.1 | 41967081 | 41967600 | up | NC_030734.1:41955822-41985100- | 10.4137931 |
| NC_030728.1 | 24717501 | 24717624 | up | NC_030728.1:24714519-24723173- | 18.10344828 |
| NC_030738.1 | 108083561 | 108083880 | up | NC_030738.1:108076064-108084184+ | 42.6 |
| NC_030738.1 | 55982261 | 55982640 | up | NC_030738.1:55929146-56005737+ | 42.6 |
| NC_030731.1 | 2935001 | 2935420 | up | NC_030731.1:2934962-2936537- | 7.762237762 |
| NC_030730.1 | 132852261 | 132852780 | up | NC_030730.1:132800458-132873704- | 11.4137931 |
| NC_030732.1 | 127015461 | 127015940 | up | NC_030732.1:127014021-127017022- | 7.366492147 |
| NC_030734.1 | 153464882 | 153465028 | up | NC_030734.1:153464668-153466118- | 66.4 |
| NC_030725.1 | 89999301 | 89999840 | up | NC_030725.1:89990764-90008652- | 12.96551724 |
| NC_030725.1 | 61882441 | 61882631 | up | NC_030725.1:61877102-61884573- | 51.5 |
| NC_030738.1 | 3673741 | 3674120 | up | NC_030738.1:3648726-3676900+ | 20.0862069 |
| NC_030728.1 | 72777261 | 72777820 | up | NC_030728.1:72740935-72780169- | 4.448979592 |
| NC_030730.1 | 115344681 | 115345080 | up | NC_030730.1:115335286-115346965- | 78.2 |
| NC_030734.1 | 84093841 | 84094320 | up | NC_030734.1:84046010-84099457+ | 72.3 |
| NC_030731.1 | 70147621 | 70148480 | up | NC_030731.1:70145975-70160041- | 4.59057072 |
| NC_030730.1 | 113251981 | 113252620 | up | NC_030730.1:113238058-113272018+ | 12.85981308 |
| NC_030727.1 | 35508321 | 35508696 | up | NC_030727.1:35503797-35508696- | 5.963350785 |
| NC_030732.1 | 58886882 | 58887069 | up | NC_030732.1:58886883-58887069+ | 60.4 |
| NC_030729.1 | 14575581 | 14575976 | up | NC_030729.1:14566445-14575976- | 4.995238095 |
| NC_030728.1 | 119600556 | 119600674 | up | NC_030728.1:119600557-119604827+ | 45.5 |
| NC_030726.1 | 60272521 | 60273100 | up | NC_030726.1:60269027-60274466+ | 6.368217054 |
| NC_030733.1 | 110359501 | 110360000 | up | NC_030733.1:110343907-110365771+ | 60.4 |
| NC_030735.1 | 88128361 | 88128581 | up | NC_030735.1:88127758-88128581- | 6.924855491 |
| NC_030726.1 | 60271761 | 60272220 | up | NC_030726.1:60269027-60274466+ | 8.700934579 |
| NC_030724.1 | 59758601 | 59759240 | up | NC_030724.1:59730834-59768634+ | 42.6 |
| NC_030724.1 | 9245781 | 9245929 | up | NC_030724.1:9230753-9245929- | 12.44827586 |
| NC_030728.1 | 121831250 | 121831346 | up | NC_030728.1:121826759-121834119- | 54.5 |
| NC_030741.1 | 83217541 | 83217920 | up | NC_030741.1:83187829-83229601+ | 93.1 |
| NC_030730.1 | 28389561 | 28390040 | up | NC_030730.1:28370802-28412523+ | 54.3 |
| NC_030729.1 | 48147281 | 48147660 | up | NC_030729.1:48137604-48150995- | 42.6 |
| NC_030727.1 | 36358321 | 36358368 | up | NC_030727.1:36357492-36358368- | 5.287662338 |
| NC_030734.1 | 62347521 | 62347760 | up | NC_030734.1:62291069-62364859+ | 45.6 |
| NC_030735.1 | 25936921 | 25937480 | up | NC_030735.1:25909340-25938076+ | 9.53271028 |
| NC_030738.1 | 61190541 | 61190920 | up | NC_030738.1:61187176-61195536- | 42.6 |
| NC_030724.1 | 142868301 | 142868780 | up | NC_030724.1:142835503-142871738+ | 54.4 |
| NC_030730.1 | 21874841 | 21875280 | up | NC_030730.1:21874319-21923139- | 78.2 |
| NC_030725.1 | 114893721 | 114894000 | up | NC_030725.1:114849418-114909834- | 54.5 |
| NC_030740.1 | 26833481 | 26834040 | up | NC_030740.1:26828535-26897319+ | 107.9 |
| NC_030727.1 | 79325121 | 79325480 | up | NC_030727.1:79317481-79326031+ | 42.6 |
| NC_030730.1 | 90983081 | 90983640 | up | NC_030730.1:90977189-91001307+ | 66.2 |
| NC_030736.1 | 56166981 | 56167580 | up | NC_030736.1:56155866-56173055+ | 4.328244275 |
| NC_030727.1 | 155904481 | 155905020 | up | NC_030727.1:155897572-155911596- | 51.5 |
| NC_030730.1 | 28372961 | 28373760 | up | NC_030730.1:28370802-28412523+ | 5.895953757 |
| NC_030734.1 | 47949821 | 47950320 | up | NC_030734.1:47915804-47962405- | 11.43103448 |
| NC_030737.1 | 4506921 | 4507240 | up | NC_030737.1:4501523-4512626- | 45.5 |
| NC_030736.1 | 52107521 | 52107960 | up | NC_030736.1:52034286-52133494- | 8.14953271 |
| NC_030730.1 | 15065812 | 15065946 | up | NC_030730.1:15065813-15065946+ | 66.3 |
| NC_030732.1 | 105472721 | 105473280 | up | NC_030732.1:105467083-105486317- | 66.3 |
| NC_030735.1 | 45818561 | 45819020 | up | NC_030735.1:45818010-45898775+ | 48.5 |
| NC_030726.1 | 7531841 | 7532300 | up | NC_030726.1:7512968-7544722+ | 4.991666667 |
| NC_030725.1 | 74177801 | 74178200 | up | NC_030725.1:74167606-74181704- | 8.14953271 |
| NC_030724.1 | 181300737 | 181300820 | up | NC_030724.1:181299084-181311849- | 15.55844156 |
| NC_030734.1 | 22143824 | 22143908 | up | NC_030734.1:22143825-22145832- | 51.5 |
| NC_030735.1 | 45821701 | 45822400 | up | NC_030735.1:45818010-45898775+ | 4.557971014 |
| NC_030727.1 | 156501261 | 156501740 | up | NC_030727.1:156499293-156517836+ | 57.5 |
| NC_030726.1 | 21842621 | 21843080 | up | NC_030726.1:21836970-21852015+ | 7.972027972 |
| NC_030741.1 | 35715401 | 35715960 | up | NC_030741.1:35713912-35720643+ | 54.5 |
| NC_030738.1 | 102669601 | 102670160 | up | NC_030738.1:102668027-102685953- | 5.935840708 |
| NC_030729.1 | 71831341 | 71831760 | up | NC_030729.1:71831212-71832278- | 10.91588785 |
| NC_030724.1 | 133635121 | 133635500 | up | NC_030724.1:133602617-133648747+ | 78.2 |
| NC_030735.1 | 128356001 | 128356420 | up | NC_030735.1:128354220-128356754+ | 54.5 |
| NC_030729.1 | 112506601 | 112507160 | up | NC_030729.1:112497285-112519662+ | 4.163716814 |
| NC_030726.1 | 93269041 | 93269480 | up | NC_030726.1:93247482-93271939- | 66.3 |
| NC_030733.1 | 88151841 | 88152340 | up | NC_030733.1:88117623-88157807+ | 102.1 |
| NC_030724.1 | 58649921 | 58650420 | up | NC_030724.1:58649735-58651051- | 48.5 |
| NC_030732.1 | 56681266 | 56681560 | up | NC_030732.1:56681267-56682073+ | 78.3 |
| NC_030732.1 | 89422921 | 89423360 | up | NC_030732.1:89415715-89435674+ | 48.5 |
| NC_030737.1 | 24880441 | 24881000 | up | NC_030737.1:24833350-24881829- | 72.2 |
| NC_030737.1 | 44298461 | 44299100 | up | NC_030737.1:44290526-44338283- | 4.783382789 |
| NC_030730.1 | 137272921 | 137273380 | up | NC_030730.1:137271385-137279841- | 6.63546798 |
| NC_030727.1 | 91724081 | 91724180 | up | NC_030727.1:91703652-91724970- | 4.364583333 |
| NC_030729.1 | 107372901 | 107373580 | up | NC_030729.1:107327670-107404496- | 4.114613181 |
| NC_030725.1 | 148092621 | 148093080 | up | NC_030725.1:148065472-148096030+ | 48.5 |
| NC_030734.1 | 47991101 | 47991940 | up | NC_030734.1:47972039-48032732- | 19.08374384 |
| NC_030728.1 | 49346521 | 49347040 | up | NC_030728.1:49345248-49361553+ | 45.5 |
| NC_030725.1 | 117577921 | 117578480 | up | NC_030725.1:117519727-117586436- | 54.4 |
| NC_030734.1 | 36661741 | 36662440 | up | NC_030734.1:36661601-36680828+ | 4.745833333 |
| NC_030727.1 | 49522661 | 49523300 | up | NC_030727.1:49503950-49539388- | 6.48 |
| NC_030738.1 | 7308441 | 7308660 | up | NC_030738.1:7305133-7312053+ | 42.6 |
| NC_030725.1 | 93235021 | 93235580 | up | NC_030725.1:93230612-93252865- | 4.729064039 |
| NC_030724.1 | 163736461 | 163737040 | up | NC_030724.1:163725545-163748811- | 9.838709677 |
| NC_030725.1 | 136444501 | 136444940 | up | NC_030725.1:136397387-136464264+ | 54.5 |
| NC_030729.1 | 2412121 | 2412461 | up | NC_030729.1:2385807-2412461+ | 96 |
| NC_030727.1 | 73616021 | 73616220 | up | NC_030727.1:73613056-73620805- | 4.222222222 |
| NC_030732.1 | 9555501 | 9556040 | up | NC_030732.1:9551538-9591606- | 6.120418848 |
| NC_030733.1 | 71815501 | 71816160 | up | NC_030733.1:71814859-71830135- | 7.579439252 |
| NC_030725.1 | 119649381 | 119649780 | up | NC_030725.1:119647136-119653344- | 66.3 |
| NC_030728.1 | 19930221 | 19930680 | up | NC_030728.1:19913448-19934458+ | 8.664516129 |
| NC_030738.1 | 26243581 | 26243940 | up | NC_030738.1:26233646-26264128+ | 4.876847291 |
| NC_030731.1 | 57212361 | 57212660 | up | NC_030731.1:57201628-57213529- | 42.6 |
| NC_030731.1 | 87058081 | 87058640 | up | NC_030731.1:87026268-87086460+ | 54.5 |
| NC_030728.1 | 60600681 | 60600880 | up | NC_030728.1:60579471-60641817- | 48.4 |
| NC_030732.1 | 59559602 | 59559640 | up | NC_030732.1:59559603-59562426+ | 48.5 |
| NC_030727.1 | 90770401 | 90770960 | up | NC_030727.1:90727016-90785894+ | 57.3 |
| NC_030726.1 | 64695981 | 64696540 | up | NC_030726.1:64684764-64713466+ | 9.514018692 |
| NC_030738.1 | 70925001 | 70925380 | up | NC_030738.1:70916528-70933795- | 5.847619048 |
| NC_030732.1 | 149225181 | 149225820 | up | NC_030732.1:149218542-149284904+ | 4.150579151 |
| NC_030736.1 | 20851561 | 20852040 | up | NC_030736.1:20826788-20890256- | 51.5 |
| NC_030727.1 | 51825941 | 51826500 | up | NC_030727.1:51776586-51855504- | 42.6 |
| NC_030738.1 | 113842801 | 113843520 | up | NC_030738.1:113839831-113843848+ | 119.8 |
| NC_030727.1 | 83393441 | 83393760 | up | NC_030727.1:83391839-83396069+ | 90.1 |
| NC_030738.1 | 87075372 | 87075636 | up | NC_030738.1:87075373-87076262+ | 51.4 |
| NC_030731.1 | 77430301 | 77430820 | up | NC_030731.1:77423416-77432097- | 119.9 |
| NC_030724.1 | 57380021 | 57380520 | up | NC_030724.1:57358424-57381793- | 42.6 |
| NC_030733.1 | 87669821 | 87670400 | up | NC_030733.1:87665599-87681217- | 6.429928741 |
| NC_030728.1 | 133545301 | 133545700 | up | NC_030728.1:133531243-133557486- | 72.3 |
| NC_030734.1 | 138942681 | 138942968 | up | NC_030734.1:138941473-138942968+ | 54.4 |
| NC_030727.1 | 43831361 | 43831760 | up | NC_030727.1:43816052-43847953+ | 54.5 |
| NC_030739.1 | 39616541 | 39616920 | up | NC_030739.1:39616400-39647532- | 60.4 |
| NC_030725.1 | 58435441 | 58435740 | up | NC_030725.1:58433620-58440809+ | 57.3 |
| NC_030737.1 | 89513941 | 89514500 | up | NC_030737.1:89495634-89581675- | 48.5 |
| NC_030738.1 | 91921921 | 91922200 | up | NC_030738.1:91921189-91924021+ | 60.4 |
| NC_030737.1 | 1164781 | 1165340 | up | NC_030737.1:1157751-1172705- | 5.190972222 |
| NC_030738.1 | 24167061 | 24167260 | up | NC_030738.1:24161755-24167975+ | 51.5 |
| NC_030732.1 | 151868561 | 151869100 | up | NC_030732.1:151865724-151870630- | 12.86915888 |
| NC_030726.1 | 28894901 | 28895220 | up | NC_030726.1:28891522-28930934- | 48.5 |
| NC_030737.1 | 76464121 | 76464780 | up | NC_030737.1:76444871-76469767+ | 7.221674877 |
| NC_030727.1 | 58531061 | 58532100 | up | NC_030727.1:58526189-58540012+ | 4.88996139 |
| NC_030729.1 | 51902861 | 51903400 | up | NC_030729.1:51894266-51919807+ | 10.09345794 |
| NC_030741.1 | 58157681 | 58157980 | up | NC_030741.1:58143350-58171708- | 116.9 |
| NC_030736.1 | 64298921 | 64299080 | up | NC_030736.1:64298922-64315701- | 7.951048951 |
| NC_030726.1 | 177693641 | 177693940 | up | NC_030726.1:177689502-177696399+ | 42.6 |
| NC_030730.1 | 48995501 | 48996000 | up | NC_030730.1:48977836-48997611+ | 11.43103448 |
| NC_030732.1 | 78144001 | 78144700 | up | NC_030732.1:78115596-78148608+ | 8.4 |
| NC_030733.1 | 49677021 | 49677420 | up | NC_030733.1:49668714-49683622- | 60.4 |
| NC_030737.1 | 72814353 | 72814470 | up | NC_030737.1:72814354-72827754+ | 60.3 |
| NC_030728.1 | 32061781 | 32061980 | up | NC_030728.1:32007103-32062625- | 10.9137931 |
| NC_030741.1 | 38394381 | 38394940 | up | NC_030741.1:38391889-38396199+ | 428.8 |
| NC_030740.1 | 21899761 | 21900160 | up | NC_030740.1:21870035-21904718+ | 81.2 |
| NC_030740.1 | 78707961 | 78708500 | up | NC_030740.1:78693615-78716048+ | 54.5 |
| NC_030737.1 | 83581621 | 83582140 | up | NC_030737.1:83574596-83583489+ | 10.9137931 |
| NC_030725.1 | 90141221 | 90141780 | up | NC_030725.1:90139038-90146556- | 96.1 |
| NC_030736.1 | 77222341 | 77222820 | up | NC_030736.1:77218847-77232828+ | 42.6 |
| NC_030730.1 | 94920801 | 94921360 | up | NC_030730.1:94918034-94938489- | 12.57009346 |
| NC_030735.1 | 87002361 | 87002900 | up | NC_030735.1:87000270-87010674- | 78.1 |
| NC_030734.1 | 51326541 | 51327120 | up | NC_030734.1:51319661-51329727- | 188 |
| NC_030732.1 | 117108981 | 117110040 | up | NC_030732.1:117102343-117147778+ | 5.462650602 |
| NC_030728.1 | 19554361 | 19554920 | up | NC_030728.1:19532331-19576644- | 4.616666667 |
| NC_030736.1 | 36504741 | 36505120 | up | NC_030736.1:36490382-36505837- | 108 |
| NC_030726.1 | 107668414 | 107668485 | up | NC_030726.1:107668415-107672445+ | 72.3 |
| NC_030728.1 | 52415161 | 52415840 | up | NC_030728.1:52399683-52450464+ | 113.9 |
| NC_030739.1 | 31451481 | 31451920 | up | NC_030739.1:31435865-31468248+ | 10.4137931 |
| NC_030728.1 | 72780341 | 72780700 | up | NC_030728.1:72780023-72789659+ | 4.093333333 |
| NC_030729.1 | 53895721 | 53896000 | up | NC_030729.1:53825546-53901738+ | 4.879844961 |
| NC_030734.1 | 50635021 | 50635540 | up | NC_030734.1:50631842-50647674- | 84.2 |
| NC_030726.1 | 139798201 | 139798420 | up | NC_030726.1:139792143-139799674- | 7.037383178 |
| NC_030740.1 | 13404681 | 13405280 | up | NC_030740.1:13398508-13406543+ | 5.038554217 |
| NC_030728.1 | 134519901 | 134520460 | up | NC_030728.1:134515501-134525279+ | 15.63551402 |
| NC_030738.1 | 4065222 | 4065560 | up | NC_030738.1:4065223-4073012- | 12.98275862 |
| NC_030732.1 | 123270761 | 123271320 | up | NC_030732.1:123244426-123276657+ | 4.99047619 |
| NC_030726.1 | 70541437 | 70542200 | up | NC_030726.1:70541438-70542515+ | 10.98709677 |
| NC_030728.1 | 59014361 | 59014920 | up | NC_030728.1:58991654-59042394+ | 51.5 |
| NC_030738.1 | 10881001 | 10881460 | up | NC_030738.1:10837350-10888110+ | 42.6 |
| NC_030739.1 | 91135581 | 91136160 | up | NC_030739.1:91118535-91136706- | 4.200573066 |
| NC_030730.1 | 112956826 | 112956920 | up | NC_030730.1:112956827-112958974+ | 4.396825397 |
| NC_030729.1 | 18571841 | 18572360 | up | NC_030729.1:18561325-18581555+ | 90.1 |
| NC_030731.1 | 92744630 | 92744920 | up | NC_030731.1:92744631-92747332+ | 60.4 |
| NC_030728.1 | 133600181 | 133600700 | up | NC_030728.1:133599769-133614524- | 60.4 |
| NC_030734.1 | 50333401 | 50333780 | up | NC_030734.1:50304542-50356335- | 54.5 |
| NC_030734.1 | 50351281 | 50351840 | up | NC_030734.1:50304542-50356335- | 10.9137931 |
| NC_030726.1 | 68354981 | 68355460 | up | NC_030726.1:68347310-68362660- | 51.4 |
| NC_030725.1 | 128529581 | 128529960 | up | NC_030725.1:128505751-128531059- | 78.3 |
| NC_030732.1 | 5695261 | 5695780 | up | NC_030732.1:5693122-5697201- | 5.245833333 |
| NC_030726.1 | 80725578 | 80725640 | up | NC_030726.1:80725579-80725825- | 60.4 |
| NC_030733.1 | 71922881 | 71923420 | up | NC_030733.1:71910481-71941210+ | 72.3 |
| NC_030739.1 | 48704041 | 48704600 | up | NC_030739.1:48685824-48739989+ | 10.19075145 |
| NC_030733.1 | 907603 | 907720 | up | NC_030733.1:907604-909490- | 66.4 |
| NC_030728.1 | 136826061 | 136826600 | up | NC_030728.1:136817356-136827850+ | 4.239608802 |
| NC_030724.1 | 199280181 | 199281480 | up | NC_030724.1:199274419-199308721- | 4.548262548 |
| NC_030740.1 | 78713081 | 78713520 | up | NC_030740.1:78693615-78716048+ | 72.3 |
| NC_030724.1 | 49172281 | 49172660 | up | NC_030724.1:49151630-49202844+ | 42.6 |
| NC_030726.1 | 147053921 | 147054300 | up | NC_030726.1:147046311-147072314+ | 48.4 |
| NC_030736.1 | 87779721 | 87780080 | up | NC_030736.1:87751078-87782925+ | 4.222222222 |
| NC_030724.1 | 110858961 | 110859420 | up | NC_030724.1:110848639-110863409- | 60.4 |
| NC_030733.1 | 107177561 | 107178020 | up | NC_030733.1:107171949-107194738+ | 9.803738318 |
| NC_030732.1 | 97300761 | 97301320 | up | NC_030732.1:97286623-97345147+ | 119.8 |
| NC_030736.1 | 67718861 | 67719420 | up | NC_030736.1:67681783-67731942- | 72.3 |
| NC_030727.1 | 114711601 | 114712260 | up | NC_030727.1:114673916-114717487- | 9.38961039 |
| NC_030731.1 | 29599741 | 29600300 | up | NC_030731.1:29572410-29601829+ | 51.4 |
| NC_030736.1 | 29745801 | 29746200 | up | NC_030736.1:29717369-29746357+ | 4.269984917 |
| NC_030732.1 | 156032901 | 156033460 | up | NC_030732.1:156022969-156066499+ | 7.329032258 |
| NC_030740.1 | 7721141 | 7721780 | up | NC_030740.1:7712023-7724122+ | 140.6 |
| NC_030724.1 | 147492861 | 147493200 | up | NC_030724.1:147490683-147526471- | 42.6 |
| NC_030737.1 | 74303458 | 74303580 | up | NC_030737.1:74303459-74303770+ | 8.152 |
| NC_030729.1 | 47256201 | 47256580 | up | NC_030729.1:47236230-47272432+ | 6.06884058 |
| NC_030727.1 | 55403041 | 55403420 | up | NC_030727.1:55400728-55456104- | 6.231213873 |
| NC_030732.1 | 128809761 | 128810300 | up | NC_030732.1:128794248-128838953- | 131.5 |
| NC_030727.1 | 76859341 | 76859860 | up | NC_030727.1:76834359-76871743- | 105 |
| NC_030738.1 | 11979761 | 11980220 | up | NC_030738.1:11978576-11988740- | 114 |
| NC_030735.1 | 34041261 | 34041680 | up | NC_030735.1:34017867-34042915- | 42.6 |
| NC_030732.1 | 60866721 | 60867220 | up | NC_030732.1:60854924-60875352+ | 60.4 |
| NC_030724.1 | 87474981 | 87475480 | up | NC_030724.1:87473490-87484401+ | 60.4 |
| NC_030732.1 | 55525981 | 55526540 | up | NC_030732.1:55502638-55539526- | 6.72972973 |
| NC_030740.1 | 26120761 | 26121180 | up | NC_030740.1:26102651-26129838+ | 4.280802292 |
| NC_030724.1 | 86739621 | 86740120 | up | NC_030724.1:86739361-86741847- | 4.231884058 |
| NC_030726.1 | 101666801 | 101667260 | up | NC_030726.1:101666026-101670018+ | 63.4 |
| NC_030738.1 | 99113841 | 99114373 | up | NC_030738.1:99105817-99114373+ | 5.534986714 |
| NC_030730.1 | 112667901 | 112668460 | up | NC_030730.1:112658344-112684621+ | 5.610837438 |
| NC_030736.1 | 124218101 | 124218320 | up | NC_030736.1:124217468-124219298- | 57.3 |
| NC_030735.1 | 75505281 | 75505680 | up | NC_030735.1:75497564-75527921- | 78.3 |
| NC_030730.1 | 144067481 | 144068180 | up | NC_030730.1:144019758-144071521+ | 60.4 |
| NC_030732.1 | 62018501 | 62019040 | up | NC_030732.1:61939184-62025442- | 6.414285714 |
| NC_030738.1 | 63657201 | 63657660 | up | NC_030738.1:63654392-63665787- | 11.44827586 |
| NC_030727.1 | 131587961 | 131588480 | up | NC_030727.1:131534848-131590150- | 42.6 |
| NC_030728.1 | 85320401 | 85320780 | up | NC_030728.1:85296045-85323213+ | 45.6 |
| NC_030736.1 | 68721901 | 68722600 | up | NC_030736.1:68714657-68733019- | 7.125874126 |
| NC_030735.1 | 41502801 | 41503200 | up | NC_030735.1:41487138-41534145- | 45.6 |
| NC_030724.1 | 136726001 | 136726480 | up | NC_030724.1:136704304-136741846- | 54.4 |
| NC_030731.1 | 87081841 | 87082220 | up | NC_030731.1:87026268-87086460+ | 9.252336449 |
| NC_030727.1 | 103871461 | 103872000 | up | NC_030727.1:103838870-103878228+ | 60.4 |
| NC_030734.1 | 151307301 | 151307860 | up | NC_030734.1:151295820-151309909+ | 42.6 |
| NC_030736.1 | 68971321 | 68971820 | up | NC_030736.1:68957582-68981866+ | 7.869158879 |
| NC_030728.1 | 46443961 | 46444500 | up | NC_030728.1:46429558-46452954+ | 6.976 |
| NC_030734.1 | 58325881 | 58325971 | up | NC_030734.1:58324306-58326672- | 60.4 |
| NC_030734.1 | 70496661 | 70497260 | up | NC_030734.1:70463407-70498492+ | 8.58041958 |
| NC_030734.1 | 95099281 | 95099860 | up | NC_030734.1:95034900-95112827+ | 7.132867133 |
| NC_030728.1 | 134353741 | 134354560 | up | NC_030728.1:134325873-134364159- | 5.019704433 |
| NC_030738.1 | 110763141 | 110763680 | up | NC_030738.1:110737670-110767296- | 12.07792208 |
| NC_030740.1 | 26224581 | 26225300 | up | NC_030740.1:26193657-26246000+ | 6.489130435 |
| NC_030733.1 | 25293361 | 25294160 | up | NC_030733.1:25276600-25305821- | 8.140186916 |
| NC_030730.1 | 69988581 | 69989140 | up | NC_030730.1:69977507-70022250+ | 6.487684729 |
| NC_030724.1 | 114678521 | 114678980 | up | NC_030724.1:114675897-114696750- | 54.5 |
| NC_030735.1 | 119691121 | 119691440 | up | NC_030735.1:119686675-119699889- | 4.319654428 |
| NC_030740.1 | 19548541 | 19549220 | up | NC_030740.1:19530732-19565868- | 11.2987013 |
| NC_030726.1 | 66828041 | 66828600 | up | NC_030726.1:66816860-66833970- | 4.461971831 |
| NC_030730.1 | 60836021 | 60836560 | up | NC_030730.1:60770414-60848459+ | 544.8 |
| NC_030728.1 | 70250561 | 70250840 | up | NC_030728.1:70245847-70265161- | 51.5 |
| NC_030730.1 | 11330841 | 11331160 | up | NC_030730.1:11330589-11332008- | 42.5 |
| NC_030728.1 | 24296661 | 24297200 | up | NC_030728.1:24272004-24307838+ | 75.1 |
| NC_030724.1 | 204821201 | 204821560 | up | NC_030724.1:204808340-204822665- | 51.4 |
| NC_030734.1 | 39449121 | 39449480 | up | NC_030734.1:39419682-39450968- | 19.65517241 |
| NC_030732.1 | 58963281 | 58963303 | up | NC_030732.1:58962774-58963303+ | 10.91588785 |
| NC_030738.1 | 110756141 | 110756820 | up | NC_030738.1:110737670-110767296- | 4.544444444 |
| NC_030724.1 | 177815921 | 177816480 | up | NC_030724.1:177812669-177817622- | 66.3 |
| NC_030734.1 | 112641541 | 112642100 | up | NC_030734.1:112631933-112670446- | 107.9 |
| NC_030724.1 | 112525941 | 112526520 | up | NC_030724.1:112520929-112529129- | 9.242990654 |
| NC_030740.1 | 92731181 | 92731940 | up | NC_030740.1:92703038-92732549+ | 42.6 |
| NC_030735.1 | 1612501 | 1613000 | up | NC_030735.1:1592504-1623753+ | 7.36453202 |
| NC_030736.1 | 53270761 | 53271500 | up | NC_030736.1:53248825-53315622+ | 6.193548387 |
| NC_030739.1 | 7702281 | 7702820 | up | NC_030739.1:7695406-7710946- | 84.2 |
| NC_030727.1 | 29778361 | 29778720 | up | NC_030727.1:29774176-29793043+ | 9.779220779 |
| NC_030741.1 | 46933321 | 46933680 | up | NC_030741.1:46923349-46943372+ | 42.6 |
| NC_030728.1 | 46449301 | 46450120 | up | NC_030728.1:46429558-46452954+ | 4.897790055 |
| NC_030733.1 | 825361 | 825920 | up | NC_030733.1:803592-855582- | 4.733990148 |
| NC_030733.1 | 72211941 | 72212200 | up | NC_030733.1:72188920-72219789+ | 78.3 |
| NC_030729.1 | 56752421 | 56752800 | up | NC_030729.1:56742862-56755680- | 48.5 |
| NC_030725.1 | 26197501 | 26198060 | up | NC_030725.1:26177139-26209695+ | 51.5 |
| NC_030739.1 | 87256681 | 87257200 | up | NC_030739.1:87234076-87284253- | 128.8 |
| NC_030728.1 | 19192061 | 19192440 | up | NC_030728.1:19118367-19211662+ | 10.89655172 |
| NC_030734.1 | 95835121 | 95835560 | up | NC_030734.1:95803532-95839355+ | 51.5 |
| NC_030738.1 | 3332261 | 3332460 | up | NC_030738.1:3313288-3347092- | 48.5 |
| NC_030734.1 | 32061181 | 32061740 | up | NC_030734.1:32051970-32067510+ | 4.586065574 |
| NC_030732.1 | 158796761 | 158797160 | up | NC_030732.1:158786825-158810338+ | 84.1 |
| NC_030728.1 | 97358061 | 97358560 | up | NC_030728.1:97351831-97377270+ | 57.4 |
| NC_030731.1 | 26329341 | 26329720 | up | NC_030731.1:26316657-26330074+ | 42.6 |
| NC_030730.1 | 137785481 | 137785532 | up | NC_030730.1:137778485-137787401+ | 57.5 |
| NC_030734.1 | 68111341 | 68111680 | up | NC_030734.1:68111069-68126103+ | 42.6 |
| NC_030736.1 | 114060761 | 114061300 | up | NC_030736.1:114026061-114113348+ | 69.3 |
| NC_030738.1 | 85166781 | 85167320 | up | NC_030738.1:85152083-85175013+ | 81.2 |
| NC_030727.1 | 103707521 | 103707820 | up | NC_030727.1:103697446-103722426- | 9 |
| NC_030734.1 | 68887961 | 68888500 | up | NC_030734.1:68840258-68902584- | 102.1 |
| NC_030728.1 | 86081201 | 86081760 | up | NC_030728.1:86031894-86083544+ | 66.4 |
| NC_030741.1 | 23359061 | 23359671 | up | NC_030741.1:23354139-23359671+ | 87 |
| NC_030741.1 | 68979161 | 68979720 | up | NC_030741.1:68960339-68982011- | 116.8 |
| NC_030724.1 | 202668681 | 202669300 | up | NC_030724.1:202655191-202679226- | 140.6 |
| NC_030731.1 | 34409061 | 34409600 | up | NC_030731.1:34349928-34411421- | 78.3 |
| NC_030738.1 | 52196521 | 52197080 | up | NC_030738.1:52153149-52199974+ | 24.24137931 |
| NC_030731.1 | 32748881 | 32749600 | up | NC_030731.1:32741139-32759536- | 4.83902439 |
| NC_030725.1 | 52253301 | 52253860 | up | NC_030725.1:52248884-52264240+ | 4.107407407 |
| NC_030729.1 | 58578881 | 58579600 | up | NC_030729.1:58570212-58583355- | 66.4 |
| NC_030728.1 | 81388321 | 81388840 | up | NC_030728.1:81371263-81394791+ | 42.5 |
| NC_030727.1 | 145448161 | 145448720 | up | NC_030727.1:145447666-145449241+ | 5.625806452 |
| NC_030731.1 | 85761401 | 85761620 | up | NC_030731.1:85756851-85762540- | 45.6 |
| NC_030734.1 | 27114041 | 27114420 | up | NC_030734.1:27107339-27117937- | 60.4 |
| NC_030732.1 | 89067161 | 89067400 | up | NC_030732.1:89048989-89089292- | 72.3 |
| NC_030728.1 | 68581 | 68780 | up | NC_030728.1:66278-96409- | 63.4 |
| NC_030724.1 | 95247041 | 95247260 | up | NC_030724.1:95237531-95249314+ | 4.037735849 |
| NC_030735.1 | 109886960 | 109887113 | up | NC_030735.1:109886961-109887113- | 84.2 |
| NC_030728.1 | 80193821 | 80194380 | up | NC_030728.1:80188067-80199394+ | 5.020942408 |
| NC_030741.1 | 27597981 | 27598340 | up | NC_030741.1:27597758-27605898- | 48.5 |
| NC_030725.1 | 57350741 | 57351280 | up | NC_030725.1:57347391-57354895- | 84.1 |
| NC_030734.1 | 287241 | 287620 | up | NC_030734.1:278279-300750- | 4.617210682 |
| NC_030732.1 | 65494061 | 65494360 | up | NC_030732.1:65437863-65501904- | 8.797202797 |
| NC_030726.1 | 106261341 | 106261820 | up | NC_030726.1:106222527-106310030+ | 13.48275862 |
| NC_030724.1 | 30146641 | 30147080 | up | NC_030724.1:30102543-30175557- | 6.72027972 |
| NC_030734.1 | 85374361 | 85374560 | up | NC_030734.1:85373943-85381565+ | 63.4 |
| NC_030733.1 | 110360261 | 110360760 | up | NC_030733.1:110343907-110365771+ | 5.99047619 |
| NC_030725.1 | 46830541 | 46831100 | up | NC_030725.1:46823987-46831562- | 42.6 |
| NC_030732.1 | 55505801 | 55506180 | up | NC_030732.1:55502638-55539526- | 42.5 |
| NC_030736.1 | 89225281 | 89225760 | up | NC_030736.1:89225161-89254716- | 96.1 |
| NC_030737.1 | 24861701 | 24862240 | up | NC_030737.1:24833350-24881829- | 30.87931034 |
| NC_030738.1 | 1992181 | 1992800 | up | NC_030738.1:1991812-1998779+ | 6.57 |
| NC_030741.1 | 15633541 | 15634040 | up | NC_030741.1:15617936-15687625- | 45.5 |
| NC_030734.1 | 98332161 | 98332660 | up | NC_030734.1:98306809-98339971+ | 72.3 |
| NC_030726.1 | 90625481 | 90626040 | up | NC_030726.1:90614504-90660352- | 6.895287958 |
| NC_030728.1 | 1087441 | 1087800 | up | NC_030728.1:1074578-1102370+ | 42.6 |
| NC_030738.1 | 85159101 | 85159640 | up | NC_030738.1:85152083-85175013+ | 6.574193548 |
| NC_030736.1 | 119449041 | 119449205 | up | NC_030736.1:119448669-119449441- | 7.694444444 |
| NC_030734.1 | 26700581 | 26700820 | up | NC_030734.1:26697863-26701311- | 60.4 |
| NC_030735.1 | 24996761 | 24997320 | up | NC_030735.1:24988553-24997989- | 4.832 |
| NC_030726.1 | 145995121 | 145995900 | up | NC_030726.1:145992628-146044461- | 5.335078534 |
| NC_030736.1 | 68162021 | 68162220 | up | NC_030736.1:68142709-68170248+ | 60.4 |
| NC_030738.1 | 88024141 | 88024800 | up | NC_030738.1:87956897-88055456- | 5.562091503 |
| NC_030733.1 | 14113521 | 14113900 | up | NC_030733.1:14112707-14126065+ | 8.61038961 |
| NC_030726.1 | 75075261 | 75075800 | up | NC_030726.1:75069249-75082364+ | 72.3 |
| NC_030724.1 | 78056241 | 78056800 | up | NC_030724.1:78046053-78060463+ | 18.62068966 |
| NC_030732.1 | 156044881 | 156045520 | up | NC_030732.1:156022969-156066499+ | 4.683928571 |
| NC_030738.1 | 110754061 | 110754600 | up | NC_030738.1:110737670-110767296- | 4.991666667 |
| NC_030724.1 | 130561761 | 130562120 | up | NC_030724.1:130550885-130567360- | 60.4 |
| NC_030725.1 | 130491681 | 130492560 | up | NC_030725.1:130465346-130512148- | 7.480237154 |
| NC_030738.1 | 18323841 | 18324107 | up | NC_030738.1:18323651-18324107- | 42.6 |
| NC_030740.1 | 8312064 | 8312200 | up | NC_030740.1:8298006-8312216+ | 54.4 |
| NC_030741.1 | 20961581 | 20962140 | up | NC_030741.1:20954726-20967910+ | 146.6 |
| NC_030734.1 | 108245421 | 108245740 | up | NC_030734.1:108232820-108247724- | 60.4 |
| NC_030726.1 | 75305121 | 75305600 | up | NC_030726.1:75261120-75307492+ | 42.6 |
| NC_030736.1 | 117155801 | 117156340 | up | NC_030736.1:117132843-117163593+ | 6.196296296 |
| NC_030739.1 | 69814041 | 69814068 | up | NC_030739.1:69788059-69814068+ | 51.5 |
| NC_030737.1 | 63529941 | 63530480 | up | NC_030737.1:63511016-63535043+ | 102 |
| NC_030726.1 | 7537221 | 7537760 | up | NC_030726.1:7512968-7544722+ | 11.29370629 |
| NC_030740.1 | 26238341 | 26239000 | up | NC_030740.1:26193657-26246000+ | 4.157303371 |
| NC_030730.1 | 111364921 | 111365480 | up | NC_030730.1:111362095-111385522+ | 48.5 |
| NC_030739.1 | 26719401 | 26719880 | up | NC_030739.1:26716126-26720598+ | 42.6 |
| NC_030735.1 | 89343101 | 89343360 | up | NC_030735.1:89342590-89412284- | 63.4 |
| NC_030724.1 | 211164871 | 211165032 | up | NC_030724.1:211161278-211170193+ | 51.5 |
| NC_030734.1 | 105975121 | 105975480 | up | NC_030734.1:105963223-105976271+ | 48.5 |
| NC_030728.1 | 79701 | 80240 | up | NC_030728.1:66278-96409- | 5.097605893 |
| NC_030732.1 | 110818421 | 110818980 | up | NC_030732.1:110818083-110819953- | 167.4 |
| NC_030730.1 | 94896061 | 94896340 | up | NC_030730.1:94894527-94900175- | 42.6 |
| NC_030736.1 | 39888461 | 39889000 | up | NC_030736.1:39865833-39890228- | 45.6 |
| NC_030741.1 | 75802341 | 75802540 | up | NC_030741.1:75798926-75807569+ | 51.5 |
| NC_030728.1 | 40981961 | 40982500 | up | NC_030728.1:40970468-40998001+ | 51.4 |
| NC_030724.1 | 201432989 | 201433090 | up | NC_030724.1:201430965-201433090- | 60.4 |
| NC_030727.1 | 108039421 | 108039980 | up | NC_030727.1:108015823-108051088+ | 16.05172414 |
| NC_030740.1 | 52105561 | 52106120 | up | NC_030740.1:52098337-52116131+ | 23.20689655 |
| NC_030731.1 | 70429261 | 70429309 | up | NC_030731.1:70427028-70429309- | 125.8 |
| NC_030729.1 | 14574441 | 14575060 | up | NC_030729.1:14566445-14575976- | 187.9 |
| NC_030727.1 | 51475701 | 51476440 | up | NC_030727.1:51451779-51524723+ | 7.341269841 |
| NC_030741.1 | 33693991 | 33694093 | up | NC_030741.1:33693992-33699285+ | 45.4 |
| NC_030726.1 | 145994481 | 145995000 | up | NC_030726.1:145992628-146044461- | 54.3 |
| NC_030738.1 | 59530521 | 59531080 | up | NC_030738.1:59526299-59533396- | 9.514018692 |
| NC_030733.1 | 41904821 | 41905520 | up | NC_030733.1:41897503-41916535+ | 12.52447552 |
| NC_030731.1 | 22479641 | 22479949 | up | NC_030731.1:22473566-22479949+ | 42.6 |
| NC_030724.1 | 62191881 | 62192140 | up | NC_030724.1:62161716-62226589+ | 9 |
| NC_030729.1 | 94495341 | 94495540 | up | NC_030729.1:94475718-94511029- | 42.6 |
| NC_030733.1 | 49020481 | 49021620 | up | NC_030733.1:48989308-49066251+ | 6.336126629 |
| NC_030728.1 | 5743041 | 5743600 | up | NC_030728.1:5704219-5746914+ | 4.74204947 |
| NC_030729.1 | 41559421 | 41559960 | up | NC_030729.1:41502160-41563345+ | 45.6 |
| NC_030731.1 | 44367721 | 44368031 | up | NC_030731.1:44367422-44368031+ | 45.5 |
| NC_030726.1 | 105601016 | 105601260 | up | NC_030726.1:105601017-105601732+ | 134.6 |
| NC_030726.1 | 162361581 | 162362120 | up | NC_030726.1:162354071-162370074- | 60.4 |
| NC_030738.1 | 59583261 | 59583760 | up | NC_030738.1:59570986-59588520+ | 60.4 |
| NC_030728.1 | 123400961 | 123401160 | up | NC_030728.1:123384915-123424625- | 45.6 |
| NC_030731.1 | 67589541 | 67589920 | up | NC_030731.1:67550327-67592692- | 54.5 |
| NC_030732.1 | 127494549 | 127494660 | up | NC_030732.1:127494550-127497320+ | 10.98709677 |
| NC_030729.1 | 113825401 | 113825960 | up | NC_030729.1:113809432-113862614- | 10.15584416 |
| NC_030740.1 | 15946641 | 15947020 | up | NC_030740.1:15940176-15948424- | 51.5 |
| NC_030729.1 | 4611960 | 4612300 | up | NC_030729.1:4611961-4613294+ | 8.140186916 |
| NC_030733.1 | 61055381 | 61056280 | up | NC_030733.1:61050719-61056401+ | 4.551963048 |
| NC_030732.1 | 62003501 | 62004180 | up | NC_030732.1:61939184-62025442- | 4.767634855 |
| NC_030724.1 | 41774631 | 41775060 | up | NC_030724.1:41774632-41816150- | 66.4 |
| NC_030729.1 | 72162821 | 72163460 | up | NC_030729.1:72142126-72170803- | 21.68965517 |
| NC_030727.1 | 155076721 | 155077100 | up | NC_030727.1:155068423-155077503+ | 90.2 |
| NC_030738.1 | 11826261 | 11826460 | up | NC_030738.1:11807499-11839059+ | 45.6 |
| NC_030734.1 | 97857921 | 97858280 | up | NC_030734.1:97836628-97862293- | 48.5 |
| NC_030724.1 | 161932641 | 161933180 | up | NC_030724.1:161894780-161936563+ | 15.18181818 |
| NC_030740.1 | 19553801 | 19554340 | up | NC_030740.1:19530732-19565868- | 6 |
| NC_030738.1 | 7077981 | 7078820 | up | NC_030738.1:7077226-7095666- | 7.119047619 |
| NC_030724.1 | 89234721 | 89235180 | up | NC_030724.1:89222206-89243458- | 63.4 |
| NC_030724.1 | 78039281 | 78040060 | up | NC_030724.1:78002746-78052843+ | 9.075 |
| NC_030730.1 | 56061821 | 56062240 | up | NC_030730.1:56045603-56074142- | 48.5 |
| NC_030741.1 | 10432581 | 10433300 | up | NC_030741.1:10395650-10475723+ | 5.682170543 |
| NC_030738.1 | 7189101 | 7189108 | up | NC_030738.1:7187453-7189108- | 8.167832168 |
| NC_030736.1 | 109579801 | 109580240 | up | NC_030736.1:109575438-109599044- | 45.5 |
| NC_030728.1 | 136827101 | 136827480 | up | NC_030728.1:136817356-136827850+ | 60.2 |
| NC_030726.1 | 42305841 | 42306220 | up | NC_030726.1:42300907-42306335+ | 42.6 |
| NC_030724.1 | 85203667 | 85204040 | up | NC_030724.1:85203668-85204487+ | 7.317757009 |
| NC_030739.1 | 90310521 | 90311160 | up | NC_030739.1:90306464-90337639+ | 12.30841121 |
| NC_030731.1 | 26416761 | 26417620 | up | NC_030731.1:26414920-26464888+ | 5.486146096 |
| NC_030736.1 | 87777981 | 87778740 | up | NC_030736.1:87751078-87782925+ | 4.347181009 |
| NC_030726.1 | 82826961 | 82827480 | up | NC_030726.1:82813289-82833187+ | 72.3 |
| NC_030730.1 | 23868961 | 23869500 | up | NC_030730.1:23862843-23870058+ | 57.4 |
| NC_030733.1 | 57042481 | 57043040 | up | NC_030733.1:57041721-57048583+ | 16.31168831 |
| NC_030725.1 | 69021101 | 69021560 | up | NC_030725.1:69020479-69026529+ | 42.6 |
| NC_030724.1 | 173480781 | 173481220 | up | NC_030724.1:173480072-173481627+ | 54.4 |
| NC_030730.1 | 110346440 | 110346557 | up | NC_030730.1:110345163-110348879+ | 9.252336449 |
| NC_030727.1 | 108015822 | 108016060 | up | NC_030727.1:108015823-108051088+ | 54.5 |
| NC_030728.1 | 7644081 | 7644540 | up | NC_030728.1:7628695-7711913- | 12.98275862 |
| NC_030724.1 | 152923081 | 152923440 | up | NC_030724.1:152913532-152932853- | 5.612546125 |
| NC_030726.1 | 76443461 | 76443573 | up | NC_030726.1:76442268-76443573+ | 8.096899225 |
| NC_030740.1 | 3790984 | 3791107 | up | NC_030740.1:3788241-3794234- | 54.4 |
| NC_030739.1 | 41888001 | 41888540 | up | NC_030739.1:41861007-41937551+ | 66.3 |
| NC_030740.1 | 42957441 | 42957900 | up | NC_030740.1:42950655-42970394- | 42.6 |
| NC_030732.1 | 112439861 | 112440100 | up | NC_030732.1:112382469-112452267- | 51.5 |
| NC_030734.1 | 68117681 | 68118300 | up | NC_030734.1:68111069-68126103+ | 4.795640327 |
| NC_030738.1 | 109447242 | 109447346 | up | NC_030738.1:109447243-109450540+ | 12.92610837 |
| NC_030733.1 | 107085241 | 107085620 | up | NC_030733.1:107072524-107092493- | 8.953271028 |
| NC_030733.1 | 47532821 | 47533400 | up | NC_030733.1:47532658-47559167- | 66.3 |
| NC_030733.1 | 68282321 | 68282780 | up | NC_030733.1:68261780-68291776- | 72.3 |
| NC_030739.1 | 39619981 | 39620700 | up | NC_030739.1:39616400-39647532- | 63.3 |
| NC_030724.1 | 219420881 | 219421260 | up | NC_030724.1:219400130-219459396+ | 87.2 |
| NC_030732.1 | 87054881 | 87055440 | up | NC_030732.1:87053700-87056912+ | 69.3 |
| NC_030729.1 | 13628821 | 13629212 | up | NC_030729.1:13627264-13629212+ | 5.974074074 |
| NC_030724.1 | 202670841 | 202671400 | up | NC_030724.1:202655191-202679226- | 78.3 |
| NC_030729.1 | 94500221 | 94500620 | up | NC_030729.1:94475718-94511029- | 42.6 |
| NC_030740.1 | 105126254 | 105126402 | up | NC_030740.1:105126255-105130193- | 104.9 |
| NC_030738.1 | 23719301 | 23719641 | up | NC_030738.1:23714173-23719641- | 9.412587413 |
| NC_030724.1 | 41834021 | 41834440 | up | NC_030724.1:41811252-41840400- | 96.1 |
| NC_030735.1 | 32174081 | 32174920 | up | NC_030735.1:32157927-32175267+ | 17.05172414 |
| NC_030726.1 | 124937441 | 124937860 | up | NC_030726.1:124928619-124940980+ | 78.3 |
| NC_030736.1 | 52108441 | 52109260 | up | NC_030736.1:52034286-52133494- | 6.840277778 |
| NC_030741.1 | 74620081 | 74620580 | up | NC_030741.1:74600536-74621194+ | 42.5 |
| NC_030734.1 | 81911121 | 81911660 | up | NC_030734.1:81887844-81914177- | 14.53271028 |
| NC_030728.1 | 126626241 | 126627120 | up | NC_030728.1:126585190-126635557- | 4.794361526 |
| NC_030725.1 | 88435921 | 88436400 | up | NC_030725.1:88385035-88452229+ | 48.5 |
| NC_030725.1 | 62441361 | 62441920 | up | NC_030725.1:62398317-62455345+ | 51.4 |
| NC_030724.1 | 116773472 | 116773551 | up | NC_030724.1:116771984-116773551- | 4.400793651 |
| NC_030728.1 | 66885721 | 66886100 | up | NC_030728.1:66877890-66892187+ | 78.2 |
| NC_030727.1 | 64997601 | 64998220 | up | NC_030727.1:64993586-65004068+ | 16.32467532 |
| NC_030732.1 | 50712581 | 50713120 | up | NC_030732.1:50672751-50716322+ | 42.6 |
| NC_030737.1 | 77190641 | 77191020 | up | NC_030737.1:77125808-77217134- | 66.3 |
| NC_030729.1 | 77631421 | 77632240 | up | NC_030729.1:77626647-77633200- | 10.03472222 |
| NC_030733.1 | 34499801 | 34500060 | up | NC_030733.1:34483227-34511810- | 15.96103896 |
| NC_030737.1 | 64069941 | 64070400 | up | NC_030737.1:64064680-64073950+ | 10.8817734 |
| NC_030732.1 | 62677121 | 62677740 | up | NC_030732.1:62672221-62727059+ | 16.03448276 |
| NC_030734.1 | 68848721 | 68849520 | up | NC_030734.1:68840258-68902584- | 63.3 |
| NC_030729.1 | 107417701 | 107418260 | up | NC_030729.1:107386817-107427149- | 13.14953271 |
| NC_030740.1 | 87459021 | 87459400 | up | NC_030740.1:87439001-87473628+ | 134.5 |
| NC_030724.1 | 161462601 | 161463100 | up | NC_030724.1:161447226-161479051- | 7.132867133 |
| NC_030734.1 | 67266841 | 67267280 | up | NC_030734.1:67235050-67286740+ | 45.5 |
| NC_030740.1 | 113113101 | 113113660 | up | NC_030740.1:113111155-113116438- | 7.958041958 |
| NC_030730.1 | 19091064 | 19091205 | up | NC_030730.1:19091065-19099463+ | 78.3 |
| NC_030735.1 | 89343441 | 89343660 | up | NC_030735.1:89342590-89412284- | 51.5 |
| NC_030726.1 | 96284201 | 96284740 | up | NC_030726.1:96266064-96315964+ | 66.4 |
| NC_030736.1 | 50184081 | 50184580 | up | NC_030736.1:50139623-50188849- | 11.93103448 |
| NC_030726.1 | 26919381 | 26919940 | up | NC_030726.1:26876493-26954046- | 78.3 |
| NC_030726.1 | 54234521 | 54234900 | up | NC_030726.1:54209952-54254306- | 63.4 |
| NC_030730.1 | 64690556 | 64690560 | up | NC_030730.1:64681299-64694375+ | 45.6 |
| NC_030730.1 | 65091432 | 65091620 | up | NC_030730.1:65091433-65093926+ | 63.4 |
| NC_030740.1 | 1361281 | 1361681 | up | NC_030740.1:1360933-1361681+ | 4.249329759 |
| NC_030737.1 | 37297621 | 37298120 | up | NC_030737.1:37248833-37329967- | 7.456 |
| NC_030733.1 | 23056803 | 23056872 | up | NC_030733.1:23054849-23056872+ | 10.69565217 |
| NC_030724.1 | 156901181 | 156901304 | up | NC_030724.1:156898374-156901304+ | 9.073298429 |
| NC_030732.1 | 54628501 | 54629420 | up | NC_030732.1:54616134-54635753+ | 113.9 |
| NC_030732.1 | 117129961 | 117130800 | up | NC_030732.1:117102343-117147778+ | 8.490322581 |
| NC_030738.1 | 30347461 | 30347840 | up | NC_030738.1:30343069-30362191+ | 5.133333333 |
| NC_030740.1 | 51178041 | 51178420 | up | NC_030740.1:51173392-51184028+ | 45.5 |
| NC_030725.1 | 119934021 | 119934400 | up | NC_030725.1:119932322-119934628+ | 48.5 |
| NC_030737.1 | 58379329 | 58379418 | up | NC_030737.1:58378094-58390800- | 72.3 |
| NC_030738.1 | 107355241 | 107355720 | up | NC_030738.1:107352543-107356644+ | 42.5 |
| NC_030724.1 | 209698521 | 209699080 | up | NC_030724.1:209696554-209710727- | 66.2 |
| NC_030737.1 | 37274641 | 37275200 | up | NC_030737.1:37248833-37329967- | 69.2 |
| NC_030728.1 | 82718141 | 82718500 | up | NC_030728.1:82696166-82738462- | 60.4 |
| NC_030724.1 | 77377901 | 77378290 | up | NC_030724.1:77345827-77378290- | 101.9 |
| NC_030732.1 | 156040101 | 156040880 | up | NC_030732.1:156022969-156066499+ | 4.588114754 |
| NC_030734.1 | 13006201 | 13006420 | up | NC_030734.1:12984427-13024792+ | 4.392735528 |
| NC_030738.1 | 42153721 | 42154160 | up | NC_030738.1:42122084-42182266- | 42.6 |
| NC_030730.1 | 65882801 | 65883360 | up | NC_030730.1:65882281-65891019+ | 6.482758621 |
| NC_030727.1 | 143486961 | 143487475 | up | NC_030727.1:143477607-143487475- | 5.816618911 |
| NC_030732.1 | 54862161 | 54862920 | up | NC_030732.1:54840367-54869231- | 4.651851852 |
| NC_030733.1 | 81212161 | 81212460 | up | NC_030733.1:81203492-81215706- | 48.5 |
| NC_030728.1 | 17194821 | 17195280 | up | NC_030728.1:17186388-17196807+ | 5.811518325 |
| NC_030726.1 | 7538121 | 7538660 | up | NC_030726.1:7512968-7544722+ | 6.923076923 |
| NC_030730.1 | 42015968 | 42015980 | up | NC_030730.1:42007996-42016056+ | 42.6 |
| NC_030724.1 | 111391401 | 111391780 | up | NC_030724.1:111372219-111400571- | 57.5 |
| NC_030726.1 | 77858481 | 77858740 | up | NC_030726.1:77854588-77861244+ | 42.6 |
| NC_030728.1 | 7746581 | 7747540 | up | NC_030728.1:7742802-7750650- | 9.523364486 |
| NC_030726.1 | 39958161 | 39958620 | up | NC_030726.1:39954076-39971640- | 66.3 |
| NC_030725.1 | 48231921 | 48232420 | up | NC_030725.1:48183697-48277006+ | 18.66233766 |
| NC_030741.1 | 94059881 | 94060280 | up | NC_030741.1:94040702-94069184- | 173.4 |
| NC_030736.1 | 110037361 | 110037920 | up | NC_030736.1:110021064-110041150- | 69.3 |
| NC_030734.1 | 153698741 | 153699200 | up | NC_030734.1:153698550-153699719- | 42.6 |
| NC_030732.1 | 54621921 | 54622480 | up | NC_030732.1:54616134-54635753+ | 54.4 |
| NC_030732.1 | 78119721 | 78120040 | up | NC_030732.1:78115596-78148608+ | 45.6 |
| NC_030735.1 | 99542081 | 99542640 | up | NC_030735.1:99524246-99564285+ | 5.245833333 |
| NC_030731.1 | 50701761 | 50702320 | up | NC_030731.1:50670102-50720455+ | 42.6 |
| NC_030740.1 | 18919701 | 18920000 | up | NC_030740.1:18903164-18920752+ | 13.14018692 |
| NC_030733.1 | 135311821 | 135312360 | up | NC_030733.1:135307725-135317705- | 45.6 |
| NC_030724.1 | 199294721 | 199295100 | up | NC_030724.1:199274419-199308721- | 66.4 |
| NC_030728.1 | 19137681 | 19138620 | up | NC_030728.1:19118367-19211662+ | 11.47663551 |
| NC_030735.1 | 64033941 | 64034440 | up | NC_030735.1:64030106-64037280- | 54.5 |
| NC_030737.1 | 4267221 | 4267500 | up | NC_030737.1:4266554-4272268+ | 6.006944444 |
| NC_030724.1 | 130502121 | 130502520 | up | NC_030724.1:130492408-130512444+ | 5.901477833 |
| NC_030730.1 | 91161616 | 91161887 | up | NC_030730.1:91161617-91161887+ | 84.1 |
| NC_030732.1 | 133961881 | 133962620 | up | NC_030732.1:133951778-133970946+ | 7.220125786 |
| NC_030724.1 | 74727161 | 74727660 | up | NC_030724.1:74726337-74730038+ | 54.4 |
| NC_030733.1 | 68598961 | 68599960 | up | NC_030733.1:68560883-68632668- | 4.30859375 |
| NC_030728.1 | 42207161 | 42207500 | up | NC_030728.1:42204238-42221361+ | 48.5 |
| NC_030740.1 | 8150341 | 8150560 | up | NC_030740.1:8149013-8153176+ | 9.376623377 |
| NC_030733.1 | 135589901 | 135590220 | up | NC_030733.1:135570977-135592453- | 5.722543353 |
| NC_030732.1 | 34798441 | 34798920 | up | NC_030732.1:34780977-34801045- | 17.60344828 |
| NC_030737.1 | 3713421 | 3713720 | up | NC_030737.1:3707843-3738590- | 48.5 |
| NC_030736.1 | 5220781 | 5221340 | up | NC_030736.1:5182037-5223060- | 42.6 |
| NC_030734.1 | 49682681 | 49682940 | up | NC_030734.1:49655887-49688000- | 9.38961039 |
| NC_030724.1 | 193247561 | 193248100 | up | NC_030724.1:193229266-193254807+ | 90.1 |
| NC_030728.1 | 53474061 | 53474780 | up | NC_030728.1:53456089-53537641+ | 4.87434555 |
| NC_030725.1 | 112376141 | 112376680 | up | NC_030725.1:112373686-112423017- | 87.2 |
| NC_030724.1 | 67126301 | 67126840 | up | NC_030724.1:67122652-67130333+ | 7.86 |
| NC_030735.1 | 35252121 | 35252500 | up | NC_030735.1:35251978-35259829+ | 7.018691589 |
| NC_030724.1 | 45034821 | 45035200 | up | NC_030724.1:45029589-45051252+ | 84.2 |
| NC_030739.1 | 77879981 | 77880300 | up | NC_030739.1:77878031-77890240- | 57.5 |
| NC_030738.1 | 110995361 | 110996440 | up | NC_030738.1:110987169-111007104+ | 7.535483871 |
| NC_030732.1 | 61991781 | 61992320 | up | NC_030732.1:61939184-62025442- | 5.896551724 |
| NC_030736.1 | 78219041 | 78219440 | up | NC_030736.1:78217247-78219740- | 48.5 |
| NC_030727.1 | 33933501 | 33933860 | up | NC_030727.1:33923955-33947498- | 42.6 |
| NC_030738.1 | 83336485 | 83336588 | up | NC_030738.1:83319450-83344106- | 42.6 |
| NC_030738.1 | 110988941 | 110989700 | up | NC_030738.1:110987169-111007104+ | 4.280276817 |
| NC_030730.1 | 91507821 | 91508460 | up | NC_030730.1:91494392-91551577+ | 8.420560748 |
| NC_030725.1 | 58039021 | 58039520 | up | NC_030725.1:58021045-58048458- | 12.46551724 |
| NC_030728.1 | 131412081 | 131412460 | up | NC_030728.1:131410454-131418629- | 60.3 |
| NC_030739.1 | 48710721 | 48711000 | up | NC_030739.1:48685824-48739989+ | 54.5 |
| NC_030724.1 | 219420461 | 219420800 | up | NC_030724.1:219400130-219459396+ | 9 |
| NC_030737.1 | 31865133 | 31865180 | up | NC_030737.1:31865134-31952348- | 51.4 |
| NC_030740.1 | 29227561 | 29228040 | up | NC_030740.1:29209437-29232654+ | 5.226190476 |
| NC_030726.1 | 10024681 | 10025240 | up | NC_030726.1:9979052-10034529- | 51.5 |
| NC_030726.1 | 99285841 | 99286360 | up | NC_030726.1:99272499-99292674+ | 45.5 |
| NC_030727.1 | 52167801 | 52168180 | up | NC_030727.1:52167646-52172164- | 60.4 |
| NC_030724.1 | 163788301 | 163788580 | up | NC_030724.1:163785621-163793093- | 57.5 |
| NC_030735.1 | 11946541 | 11947100 | up | NC_030735.1:11944603-11952995+ | 102 |
| NC_030727.1 | 72214881 | 72215420 | up | NC_030727.1:72213920-72220679+ | 5.728323699 |
| NC_030734.1 | 152306581 | 152307420 | up | NC_030734.1:152269026-152339382- | 122.5 |
| NC_030737.1 | 24863801 | 24864260 | up | NC_030737.1:24833350-24881829- | 137.7 |
| NC_030726.1 | 6288137 | 6288286 | up | NC_030726.1:6285053-6289894- | 13.84 |
| NC_030733.1 | 87413041 | 87413640 | up | NC_030733.1:87409484-87440693+ | 4.430267062 |
| NC_030729.1 | 56064641 | 56065040 | up | NC_030729.1:56054039-56073344+ | 48.5 |
| NC_030730.1 | 98296236 | 98296436 | up | NC_030730.1:98296237-98296436- | 66.4 |
| NC_030728.1 | 7680901 | 7681140 | up | NC_030728.1:7628695-7711913- | 45.4 |
| NC_030727.1 | 92047661 | 92048340 | up | NC_030727.1:92042286-92067178- | 107.9 |
| NC_030724.1 | 94360221 | 94360780 | up | NC_030724.1:94356008-94369760+ | 13.62337662 |
| NC_030725.1 | 117586421 | 117586780 | up | NC_030725.1:117533252-117588339- | 7.729032258 |
| NC_030734.1 | 94911301 | 94911382 | up | NC_030734.1:94880885-94911382- | 17.46753247 |
| NC_030740.1 | 3028521 | 3028525 | up | NC_030740.1:3023516-3028525- | 63.3 |
| NC_030733.1 | 90165621 | 90166180 | up | NC_030733.1:90162700-90168343+ | 81.2 |
| NC_030728.1 | 122671921 | 122672980 | up | NC_030728.1:122663424-122676493+ | 4.55758427 |
| NC_030728.1 | 5720521 | 5721260 | up | NC_030728.1:5704219-5746914+ | 10.21935484 |
| NC_030733.1 | 132961649 | 132961793 | up | NC_030733.1:132961650-132962439+ | 140.6 |
| NC_030733.1 | 87675581 | 87675920 | up | NC_030733.1:87665599-87681217- | 54.5 |
| NC_030728.1 | 117355681 | 117356240 | up | NC_030728.1:117350763-117357967- | 6.286713287 |
| NC_030733.1 | 56000761 | 56001020 | up | NC_030733.1:55988879-56002536- | 14.01724138 |
| NC_030726.1 | 21836461 | 21836840 | up | NC_030726.1:21824213-21839993+ | 75.3 |
| NC_030728.1 | 19192561 | 19193100 | up | NC_030728.1:19118367-19211662+ | 54.3 |
| NC_030727.1 | 144900781 | 144901320 | up | NC_030727.1:144899726-144903768+ | 48.5 |
| NC_030727.1 | 42600601 | 42601160 | up | NC_030727.1:42584624-42607814+ | 14 |
| NC_030728.1 | 66641 | 67080 | up | NC_030728.1:66278-96409- | 48.4 |
| NC_030738.1 | 106788521 | 106789060 | up | NC_030738.1:106783446-106827778- | 54.3 |
| NC_030726.1 | 40537461 | 40538200 | up | NC_030726.1:40507498-40553799+ | 13.23376623 |
| NC_030734.1 | 72013201 | 72013520 | up | NC_030734.1:71978005-72042575- | 105 |
| NC_030741.1 | 77376641 | 77377160 | up | NC_030741.1:77356699-77387064+ | 66.3 |
| NC_030727.1 | 92308401 | 92308780 | up | NC_030727.1:92303806-92316721+ | 66.4 |
| NC_030732.1 | 117110441 | 117110660 | up | NC_030732.1:117102343-117147778+ | 45.6 |
| NC_030734.1 | 90834521 | 90835240 | up | NC_030734.1:90822398-90837233- | 7.361256545 |
| NC_030728.1 | 32008341 | 32008740 | up | NC_030728.1:32007103-32062625- | 54.4 |
| NC_030726.1 | 98163481 | 98164020 | up | NC_030726.1:98147788-98186666- | 78.2 |
| NC_030734.1 | 3491881 | 3492440 | up | NC_030734.1:3471634-3503297- | 69.2 |
| NC_030725.1 | 103934921 | 103935440 | up | NC_030725.1:103931724-103937024- | 69.3 |
| NC_030728.1 | 5733941 | 5734320 | up | NC_030728.1:5704219-5746914+ | 10.4137931 |
| NC_030733.1 | 57826328 | 57826660 | up | NC_030733.1:57826329-57828822- | 4.136235955 |
| NC_030727.1 | 75183461 | 75183880 | up | NC_030727.1:75177898-75188236+ | 17.5862069 |
| NC_030734.1 | 126428681 | 126429020 | up | NC_030734.1:126427245-126430200+ | 87.1 |
| NC_030724.1 | 63031621 | 63032280 | up | NC_030724.1:63030948-63033663- | 7.341935484 |
| NC_030739.1 | 96178701 | 96178709 | up | NC_030739.1:96178293-96178709- | 48.5 |
| NC_030733.1 | 72155861 | 72156200 | up | NC_030733.1:72129649-72191694+ | 84.2 |
| NC_030724.1 | 69540981 | 69541660 | up | NC_030724.1:69539956-69544780- | 81.1 |
| NC_030727.1 | 37459001 | 37459520 | up | NC_030727.1:37455464-37498815+ | 4.020295203 |
| NC_030738.1 | 63656021 | 63656560 | up | NC_030738.1:63654392-63665787- | 57.3 |
| NC_030737.1 | 24873281 | 24873840 | up | NC_030737.1:24833350-24881829- | 54.4 |
| NC_030737.1 | 57754301 | 57754393 | up | NC_030737.1:57729711-57754393- | 9 |
| NC_030728.1 | 94684901 | 94685880 | up | NC_030728.1:94680725-94707426- | 4.616666667 |
| NC_030730.1 | 106344681 | 106345040 | up | NC_030730.1:106334735-106350572- | 48.5 |
| NC_030724.1 | 125053881 | 125054420 | up | NC_030724.1:125035768-125060840+ | 4.77173913 |
| NC_030740.1 | 35631024 | 35631158 | up | NC_030740.1:35631025-35631453+ | 4.495833333 |
| NC_030726.1 | 54230421 | 54230940 | up | NC_030726.1:54209952-54254306- | 42.6 |
| NC_030738.1 | 14967241 | 14967940 | up | NC_030738.1:14947742-14973216- | 5.220930233 |
| NC_030738.1 | 171801 | 172340 | up | NC_030738.1:168083-177777+ | 6.256 |
| NC_030724.1 | 69542261 | 69542620 | up | NC_030724.1:69539956-69544780- | 60.3 |
| NC_030733.1 | 91102201 | 91102760 | up | NC_030733.1:91094287-91108574+ | 4.646112601 |
| NC_030726.1 | 152818681 | 152819180 | up | NC_030726.1:152811274-152822475+ | 8.986013986 |
| NC_030727.1 | 64662881 | 64663440 | up | NC_030727.1:64657175-64672280- | 5.99047619 |
| NC_030728.1 | 118311181 | 118311720 | up | NC_030728.1:118298705-118346521+ | 48.5 |
| NC_030738.1 | 83151349 | 83151531 | up | NC_030738.1:83150533-83160693- | 4.621776504 |
| NC_030725.1 | 40155741 | 40156300 | up | NC_030725.1:40120474-40176046- | 7.859813084 |
| NC_030730.1 | 22222541 | 22223100 | up | NC_030730.1:22213724-22223464- | 54.5 |
| NC_030732.1 | 89078101 | 89078300 | up | NC_030732.1:89048989-89089292- | 42.6 |
| NC_030738.1 | 18309281 | 18309760 | up | NC_030738.1:18307446-18323735- | 7.869158879 |
| NC_030738.1 | 13900901 | 13901146 | up | NC_030738.1:13898504-13901146+ | 42.6 |
| NC_030725.1 | 133980441 | 133980820 | up | NC_030725.1:133940320-133989696+ | 54.5 |
| NC_030729.1 | 87009261 | 87010000 | up | NC_030729.1:87002342-87017043- | 4.022113022 |
| NC_030728.1 | 60929341 | 60929600 | up | NC_030728.1:60925035-60947949+ | 60.4 |
| NC_030728.1 | 75361781 | 75362620 | up | NC_030728.1:75340893-75396640- | 15.08411215 |
| NC_030738.1 | 55131701 | 55131761 | up | NC_030738.1:55131320-55136617- | 10.35514019 |
| NC_030726.1 | 145784541 | 145784840 | up | NC_030726.1:145769157-145790675+ | 57.4 |
| NC_030730.1 | 51487601 | 51487980 | up | NC_030730.1:51458746-51490555- | 87.1 |
| NC_030732.1 | 53445041 | 53445600 | up | NC_030732.1:53423582-53454672+ | 134.7 |
| NC_030739.1 | 55886541 | 55886587 | up | NC_030739.1:55872715-55886587- | 42.5 |
| NC_030737.1 | 29522801 | 29522824 | up | NC_030737.1:29517836-29522824+ | 45.5 |
| NC_030726.1 | 2251901 | 2252380 | up | NC_030726.1:2244761-2298671+ | 8.987012987 |
| NC_030730.1 | 112104121 | 112104640 | up | NC_030730.1:112089945-112109468+ | 9.20979021 |
| NC_030726.1 | 145993741 | 145994440 | up | NC_030726.1:145992628-146044461- | 5.881118881 |
| NC_030732.1 | 49529121 | 49529660 | up | NC_030732.1:49526771-49540017+ | 5.38150289 |
| NC_030738.1 | 18210661 | 18211220 | up | NC_030738.1:18199153-18226092- | 7.68 |
| NC_030739.1 | 78191321 | 78191840 | up | NC_030739.1:78182351-78193695- | 45.5 |
| NC_030730.1 | 60809481 | 60809920 | up | NC_030730.1:60770414-60848459+ | 5.096997691 |
| NC_030732.1 | 74377501 | 74378100 | up | NC_030732.1:74353708-74390265+ | 4.126811594 |
| NC_030725.1 | 64751501 | 64752120 | up | NC_030725.1:64747957-64754143- | 51.5 |
| NC_030732.1 | 10634361 | 10634780 | up | NC_030732.1:10608254-10651110+ | 48.5 |
| NC_030728.1 | 7630881 | 7631160 | up | NC_030728.1:7628695-7711913- | 48.5 |
| NC_030727.1 | 42824321 | 42824880 | up | NC_030727.1:42795012-42825474+ | 45.5 |
| NC_030734.1 | 102947041 | 102947520 | up | NC_030734.1:102946691-102996073+ | 4.989130435 |
| NC_030728.1 | 17406621 | 17407040 | up | NC_030728.1:17391642-17433701+ | 15.57142857 |
| NC_030740.1 | 1402341 | 1402693 | up | NC_030740.1:1399717-1402693+ | 8.641618497 |
| NC_030732.1 | 117136681 | 117137460 | up | NC_030732.1:117102343-117147778+ | 9.50867052 |
| NC_030734.1 | 98976001 | 98976560 | up | NC_030734.1:98965296-99020496- | 10.9137931 |
| NC_030740.1 | 52779831 | 52780060 | up | NC_030740.1:52779832-52782055- | 72.2 |
| NC_030732.1 | 132735601 | 132736500 | up | NC_030732.1:132709399-132743064- | 45.5 |
| NC_030732.1 | 70559021 | 70559461 | up | NC_030732.1:70559007-70559461- | 4.720720721 |
| NC_030732.1 | 57993781 | 57994340 | up | NC_030732.1:57982477-57995691+ | 4.583333333 |
| NC_030726.1 | 40533941 | 40534220 | up | NC_030726.1:40507498-40553799+ | 81.3 |
| NC_030726.1 | 77770721 | 77771180 | up | NC_030726.1:77769813-77772094- | 54.4 |
| NC_030728.1 | 2536021 | 2536560 | up | NC_030728.1:2513586-2551183- | 16.32467532 |
| NC_030725.1 | 13008233 | 13008640 | up | NC_030725.1:13008234-13009059+ | 6.884337349 |
| NC_030724.1 | 182924021 | 182924422 | up | NC_030724.1:182920091-182924422+ | 13.66009852 |
| NC_030736.1 | 77032721 | 77033580 | up | NC_030736.1:77020027-77039466+ | 8.973684211 |
| NC_030724.1 | 48558101 | 48558400 | up | NC_030724.1:48549189-48576698+ | 7.317757009 |
| NC_030726.1 | 146006141 | 146006820 | up | NC_030726.1:145992628-146044461- | 4.182170543 |
| NC_030728.1 | 26455521 | 26455720 | up | NC_030728.1:26435784-26456070- | 60.4 |
| NC_030728.1 | 94264141 | 94264560 | up | NC_030728.1:94239770-94280813- | 11.43103448 |
| NC_030736.1 | 52116801 | 52117360 | up | NC_030736.1:52034286-52133494- | 51.4 |
| NC_030730.1 | 645841 | 646140 | up | NC_030730.1:621366-668331- | 81.2 |
| NC_030739.1 | 90124721 | 90125560 | up | NC_030739.1:90122598-90150545+ | 7.208 |
| NC_030727.1 | 91555001 | 91555240 | up | NC_030727.1:91553042-91557101- | 45.6 |
| NC_030734.1 | 99015981 | 99016400 | up | NC_030734.1:98965296-99020496- | 84.1 |
| NC_030727.1 | 71827948 | 71828029 | up | NC_030727.1:71813401-71828554+ | 78.3 |
| NC_030734.1 | 70772441 | 70772960 | up | NC_030734.1:70762612-70819895- | 51.5 |
| NC_030737.1 | 21921261 | 21922200 | up | NC_030737.1:21918707-21970718+ | 6.237529691 |
| NC_030724.1 | 142836521 | 142837000 | up | NC_030724.1:142835503-142843470+ | 7.91152815 |
| NC_030729.1 | 80273341 | 80273900 | up | NC_030729.1:80250333-80340756+ | 113.7 |
| NC_030736.1 | 5216841 | 5217400 | up | NC_030736.1:5182037-5223060- | 75.2 |
| NC_030726.1 | 109156281 | 109156580 | up | NC_030726.1:109145473-109158690+ | 90.1 |
| NC_030732.1 | 156057761 | 156058320 | up | NC_030732.1:156022969-156066499+ | 9.53271028 |
| NC_030732.1 | 68692201 | 68692900 | up | NC_030732.1:68686066-68760771+ | 4.68 |
| NC_030736.1 | 73598241 | 73598440 | up | NC_030736.1:73595001-73602821+ | 96.1 |
| NC_030728.1 | 50750381 | 50750700 | up | NC_030728.1:50701333-50795211- | 57.5 |
| NC_030730.1 | 111087321 | 111087860 | up | NC_030730.1:111073975-111097999+ | 12.09090909 |
| NC_030740.1 | 8667201 | 8667660 | up | NC_030740.1:8627416-8677514+ | 42.6 |
| NC_030739.1 | 77994901 | 77995400 | up | NC_030739.1:77949377-78011718- | 66.4 |
| NC_030733.1 | 1087401 | 1087840 | up | NC_030733.1:1063768-1098992+ | 119.8 |
| NC_030733.1 | 48306064 | 48306180 | up | NC_030733.1:48272142-48332868+ | 4.53515625 |
| NC_030726.1 | 93373941 | 93374420 | up | NC_030726.1:93357563-93386907- | 72.3 |
| NC_030735.1 | 45867601 | 45868160 | up | NC_030735.1:45818010-45898775+ | 12.85981308 |
| NC_030727.1 | 55437381 | 55437940 | up | NC_030727.1:55400728-55456104- | 101.9 |
| NC_030727.1 | 51850981 | 51851540 | up | NC_030727.1:51776586-51855504- | 60.4 |
| NC_030734.1 | 123107101 | 123107480 | up | NC_030734.1:123082126-123138097+ | 108 |
| NC_030730.1 | 88729201 | 88729760 | up | NC_030730.1:88705632-88774092- | 54.5 |
| NC_030728.1 | 19171041 | 19171600 | up | NC_030728.1:19118367-19211662+ | 54.5 |
| NC_030731.1 | 12603781 | 12604320 | up | NC_030731.1:12580263-12609893+ | 54.4 |
| NC_030731.1 | 88484981 | 88485008 | up | NC_030731.1:88484757-88485008- | 7.929032258 |
| NC_030724.1 | 155478801 | 155479320 | up | NC_030724.1:155464464-155499720+ | 4.990990991 |
| NC_030734.1 | 50140841 | 50141140 | up | NC_030734.1:50136410-50142910+ | 10.08411215 |
| NC_030729.1 | 35196001 | 35196640 | up | NC_030729.1:35192286-35207785+ | 6.300699301 |
| NC_030734.1 | 91604261 | 91604451 | up | NC_030734.1:91604035-91604451+ | 54.5 |
| NC_030738.1 | 83093601 | 83094140 | up | NC_030738.1:83069676-83126138+ | 45.6 |
| NC_030739.1 | 95132681 | 95133180 | up | NC_030739.1:95131523-95156527+ | 10.64485981 |
| NC_030729.1 | 107288701 | 107289160 | up | NC_030729.1:107268494-107289928+ | 72.3 |
| NC_030735.1 | 103192841 | 103193420 | up | NC_030735.1:103186860-103193420- | 4.493150685 |
| NC_030739.1 | 65974821 | 65975260 | up | NC_030739.1:65971821-65980875+ | 93.1 |
| NC_030728.1 | 50300581 | 50300980 | up | NC_030728.1:50297093-50307605- | 6.72027972 |
| NC_030734.1 | 49664121 | 49664520 | up | NC_030734.1:49655887-49688000- | 4.729166667 |
| NC_030736.1 | 73233321 | 73233700 | up | NC_030736.1:73226670-73238013+ | 60.4 |
| NC_030733.1 | 71825741 | 71826180 | up | NC_030733.1:71814859-71830135- | 54.3 |
| NC_030732.1 | 37986641 | 37986980 | up | NC_030732.1:37943041-38001342+ | 54.5 |
| NC_030727.1 | 31089321 | 31089860 | up | NC_030727.1:31082728-31095469- | 45.6 |
| NC_030727.1 | 43745661 | 43746240 | up | NC_030727.1:43744888-43747591- | 4.501298701 |
| NC_030733.1 | 51844881 | 51845420 | up | NC_030733.1:51836941-51895818- | 5.625806452 |
| NC_030729.1 | 96376721 | 96377120 | up | NC_030729.1:96330375-96381729+ | 8.623376623 |
| NC_030738.1 | 26285301 | 26285712 | up | NC_030738.1:26282065-26285712- | 4.277777778 |
| NC_030727.1 | 131993801 | 131994180 | up | NC_030727.1:131971919-131999298+ | 96.1 |
| NC_030740.1 | 104115381 | 104115920 | up | NC_030740.1:104052938-104133614+ | 78.2 |
| NC_030735.1 | 54498081 | 54498400 | up | NC_030735.1:54465428-54535295+ | 48.5 |
| NC_030732.1 | 55524221 | 55524800 | up | NC_030732.1:55502638-55539526- | 7.96735905 |
| NC_030734.1 | 14468201 | 14468780 | up | NC_030734.1:14407760-14476027- | 5.138461538 |
| NC_030737.1 | 80315041 | 80315600 | up | NC_030737.1:80306590-80371329- | 48.5 |
| NC_030737.1 | 27915341 | 27916040 | up | NC_030737.1:27904202-27945062+ | 8.377622378 |
| NC_030725.1 | 92376961 | 92377160 | up | NC_030725.1:92357726-92385188+ | 10.93103448 |
| NC_030724.1 | 65832901 | 65833240 | up | NC_030724.1:65817593-65843570- | 9.012987013 |
| NC_030724.1 | 151923161 | 151923420 | up | NC_030724.1:151916198-151925943+ | 57.5 |
| NC_030736.1 | 73093378 | 73093715 | up | NC_030736.1:73093379-73093715+ | 110.9 |
| NC_030740.1 | 66534461 | 66534740 | up | NC_030740.1:66525602-66539570- | 69.4 |
| NC_030725.1 | 48901029 | 48901600 | up | NC_030725.1:48901030-48901662- | 4.98015873 |
| NC_030732.1 | 55523781 | 55524200 | up | NC_030732.1:55502638-55539526- | 7.588785047 |
| NC_030728.1 | 132407401 | 132407780 | up | NC_030728.1:132403003-132408402- | 7.43324937 |
| NC_030730.1 | 97568741 | 97569200 | up | NC_030730.1:97537498-97588328- | 60.4 |
| NC_030733.1 | 109930861 | 109931060 | up | NC_030733.1:109921602-109933542- | 42.6 |
| NC_030726.1 | 19726914 | 19727029 | up | NC_030726.1:19726915-19731641- | 51.5 |
| NC_030728.1 | 19563961 | 19564500 | up | NC_030728.1:19532331-19576644- | 75.1 |
| NC_030736.1 | 12168521 | 12168900 | up | NC_030736.1:12100267-12188354+ | 48.5 |
| NC_030736.1 | 77027881 | 77028460 | up | NC_030736.1:77020027-77039466+ | 21.71612903 |
| NC_030726.1 | 46295641 | 46296200 | up | NC_030726.1:46252404-46299558- | 78.1 |
| NC_030733.1 | 2036221 | 2036540 | up | NC_030733.1:2021873-2041627- | 54.5 |
| NC_030729.1 | 58611221 | 58611250 | up | NC_030729.1:58610798-58611250+ | 48.5 |
| NC_030727.1 | 82848561 | 82849180 | up | NC_030727.1:82814356-82850722- | 69.2 |
| NC_030727.1 | 119174581 | 119175122 | up | NC_030727.1:119173762-119175122+ | 7.358695652 |
| NC_030738.1 | 10105101 | 10105595 | up | NC_030738.1:10102030-10105595- | 66.4 |
| NC_030730.1 | 25111821 | 25112009 | up | NC_030730.1:25111226-25112009+ | 45.6 |
| NC_030738.1 | 2347138 | 2347300 | up | NC_030738.1:2347139-2358663+ | 4.171084337 |
| NC_030727.1 | 79029821 | 79030360 | up | NC_030727.1:79024706-79052083- | 90.1 |
| NC_030727.1 | 144546241 | 144546402 | up | NC_030727.1:144546242-144549468- | 6.383045526 |
| NC_030725.1 | 60424701 | 60425040 | up | NC_030725.1:60404279-60444147+ | 66.4 |
| NC_030738.1 | 116486141 | 116487120 | up | NC_030738.1:116446394-116502224- | 152.5 |
| NC_030740.1 | 5907467 | 5907551 | up | NC_030740.1:5906861-5907551+ | 6.858299595 |
| NC_030730.1 | 59651101 | 59651540 | up | NC_030730.1:59645750-59698171- | 4.809651475 |
| NC_030734.1 | 119938961 | 119939340 | up | NC_030734.1:119932137-119941801- | 93.1 |
| NC_030728.1 | 97361041 | 97361600 | up | NC_030728.1:97351831-97377270+ | 66.4 |
| NC_030734.1 | 153648535 | 153648680 | up | NC_030734.1:153648536-153648719- | 42.6 |
| NC_030734.1 | 50307721 | 50308220 | up | NC_030734.1:50290680-50318543- | 4.371232877 |
| NC_030732.1 | 56802381 | 56802860 | up | NC_030732.1:56764774-56815592+ | 69.3 |
| NC_030728.1 | 19183841 | 19184420 | up | NC_030728.1:19118367-19211662+ | 8.377622378 |
| NC_030732.1 | 40136141 | 40136540 | up | NC_030732.1:40094815-40152356+ | 60.3 |
| NC_030732.1 | 59329921 | 59330840 | up | NC_030732.1:59317677-59337960+ | 155.5 |
| NC_030740.1 | 105155961 | 105156520 | up | NC_030740.1:105149516-105169207+ | 6.193548387 |
| NC_030740.1 | 107588281 | 107588350 | up | NC_030740.1:107587122-107588350+ | 48.4 |
| NC_030735.1 | 45857281 | 45858040 | up | NC_030735.1:45818010-45898775+ | 8.649851632 |
| NC_030732.1 | 65523881 | 65524320 | up | NC_030732.1:65499495-65533192- | 78.1 |
| NC_030738.1 | 30351881 | 30352080 | up | NC_030738.1:30343069-30362191+ | 81 |
| NC_030734.1 | 113351421 | 113351960 | up | NC_030734.1:113328243-113362975- | 108 |
| NC_030736.1 | 4004321 | 4004660 | up | NC_030736.1:3998340-4017313- | 54.5 |
| NC_030724.1 | 70025741 | 70026280 | up | NC_030724.1:69982465-70034293- | 54.5 |
| NC_030733.1 | 84925661 | 84926220 | down | NC_030733.1:84923375-84965967- | 60.3 |
| NC_030727.1 | 102083001 | 102083520 | down | NC_030727.1:102078131-102132237- | 40.3 |
| NC_030733.1 | 48959821 | 48960260 | down | NC_030733.1:48942375-49018351+ | 50.6 |
| NC_030727.1 | 72007981 | 72008360 | down | NC_030727.1:71984875-72008392- | 14.15 |
| NC_030730.1 | 11631461 | 11632040 | down | NC_030730.1:11630713-11634237- | 9.173913043 |
| NC_030740.1 | 14338501 | 14339020 | down | NC_030740.1:14329209-14354181+ | 50.6 |
| NC_030736.1 | 102765963 | 102766140 | down | NC_030736.1:102765964-102800187+ | 50.6 |
| NC_030738.1 | 11892361 | 11892740 | down | NC_030738.1:11838973-11925201+ | 53 |
| NC_030738.1 | 1997541 | 1997900 | down | NC_030738.1:1991812-1998779+ | 48.8 |
| NC_030739.1 | 42374681 | 42374940 | down | NC_030739.1:42334596-42390515+ | 50.6 |
| NC_030738.1 | 3083721 | 3084280 | down | NC_030738.1:3020722-3115169- | 53.6 |
| NC_030735.1 | 96658621 | 96659160 | down | NC_030735.1:96603145-96671190+ | 44 |
| NC_030736.1 | 85503541 | 85504100 | down | NC_030736.1:85488744-85504554+ | 63.9 |
| NC_030741.1 | 60683481 | 60684200 | down | NC_030741.1:60675819-60694398- | 7.164893617 |
| NC_030728.1 | 41207941 | 41208340 | down | NC_030728.1:41196567-41215196- | 66.3 |
| NC_030725.1 | 77706921 | 77707340 | down | NC_030725.1:77673438-77708207+ | 56.6 |
| NC_030738.1 | 75125621 | 75126180 | down | NC_030738.1:75125254-75127053- | 47 |
| NC_030739.1 | 92095964 | 92096320 | down | NC_030739.1:92095965-92097223+ | 18.4 |
| NC_030730.1 | 107745561 | 107746200 | down | NC_030730.1:107741556-107749432+ | 66.3 |
| NC_030728.1 | 134520621 | 134521180 | down | NC_030728.1:134515501-134525279+ | 5.613924051 |
| NC_030741.1 | 90592281 | 90592840 | down | NC_030741.1:90562090-90596437+ | 40.3 |
| NC_030729.1 | 90831161 | 90831720 | down | NC_030729.1:90795182-90838097+ | 5.727848101 |
| NC_030724.1 | 106731401 | 106731940 | down | NC_030724.1:106729225-106756564- | 50.6 |
| NC_030725.1 | 71818161 | 71818540 | down | NC_030725.1:71790390-71820301+ | 72.4 |
| NC_030734.1 | 68900181 | 68900780 | down | NC_030734.1:68840258-68902584- | 113.5 |
| NC_030727.1 | 27238561 | 27238760 | down | NC_030727.1:27220351-27257620+ | 47 |
| NC_030733.1 | 49968661 | 49969440 | down | NC_030733.1:49949746-49972572- | 7.555555556 |
| NC_030729.1 | 25296041 | 25296451 | down | NC_030729.1:25295295-25296451+ | 47 |
| NC_030736.1 | 93383761 | 93384320 | down | NC_030736.1:93380494-93386669- | 25.5 |
| NC_030727.1 | 51804661 | 51805320 | down | NC_030727.1:51759692-51808876- | 14.52173913 |
| NC_030724.1 | 178911281 | 178911840 | down | NC_030724.1:178875686-178969784- | 93.6 |
| NC_030725.1 | 88636881 | 88637300 | down | NC_030725.1:88613715-88650437+ | 7.434343434 |
| NC_030732.1 | 90749568 | 90749632 | down | NC_030732.1:90733092-90764637+ | 23.525 |
| NC_030726.1 | 29238981 | 29239500 | down | NC_030726.1:29197325-29259269- | 47 |
| NC_030741.1 | 9054301 | 9054740 | down | NC_030741.1:9046889-9080477- | 40.3 |
| NC_030736.1 | 104026701 | 104027260 | down | NC_030736.1:104026564-104028790+ | 63.9 |
| NC_030731.1 | 115154161 | 115154540 | down | NC_030731.1:115153352-115157461- | 51.8 |
| NC_030728.1 | 99465621 | 99466020 | down | NC_030728.1:99450764-99467246- | 9.695652174 |
| NC_030739.1 | 51880881 | 51881380 | down | NC_030739.1:51845286-51884593- | 10.66666667 |
| NC_030730.1 | 112665001 | 112665900 | down | NC_030730.1:112658344-112684621+ | 4.396761134 |
| NC_030728.1 | 134179441 | 134180000 | down | NC_030728.1:134172639-134181223+ | 65.1 |
| NC_030727.1 | 83062500 | 83062618 | down | NC_030727.1:83061263-83062618- | 50.6 |
| NC_030729.1 | 87010161 | 87010920 | down | NC_030729.1:87002342-87017043- | 6.164893617 |
| NC_030737.1 | 37290841 | 37291700 | down | NC_030737.1:37248833-37329967- | 5.013761468 |
| NC_030729.1 | 67712059 | 67712186 | down | NC_030729.1:67661306-67725087+ | 50.6 |
| NC_030724.1 | 174902255 | 174902324 | down | NC_030724.1:174883985-174902645+ | 9.695652174 |
| NC_030734.1 | 49674441 | 49675120 | down | NC_030734.1:49655887-49688000- | 6.627530364 |
| NC_030725.1 | 58055741 | 58056120 | down | NC_030725.1:58050947-58056292- | 50.6 |
| NC_030734.1 | 92129101 | 92129520 | down | NC_030734.1:92126617-92138406+ | 51.8 |
| NC_030728.1 | 5717821 | 5718300 | down | NC_030728.1:5704219-5746914+ | 55.4 |
| NC_030732.1 | 59267741 | 59268880 | down | NC_030732.1:59259492-59274022- | 4.674033149 |
| NC_030724.1 | 88692321 | 88692760 | down | NC_030724.1:88688709-88712164+ | 53 |
| NC_030732.1 | 4748330 | 4748660 | down | NC_030732.1:4745419-4748660- | 51.8 |
| NC_030736.1 | 96962721 | 96963500 | down | NC_030736.1:96956853-96971877+ | 5.418502203 |
| NC_030727.1 | 131575261 | 131575480 | down | NC_030727.1:131534848-131590150- | 47 |
| NC_030738.1 | 48021021 | 48021380 | down | NC_030738.1:47992959-48028405- | 40.3 |
| NC_030738.1 | 24819201 | 24819660 | down | NC_030738.1:24808165-24823149- | 13.11594203 |
| NC_030738.1 | 83071141 | 83071680 | down | NC_030738.1:83069676-83126138+ | 163.1 |
| NC_030726.1 | 179330518 | 179330666 | down | NC_030726.1:179330519-179330666- | 62.7 |
| NC_030740.1 | 9709121 | 9709500 | down | NC_030740.1:9689469-9712044+ | 55.4 |
| NC_030740.1 | 21892021 | 21892400 | down | NC_030740.1:21870035-21904718+ | 59.1 |
| NC_030735.1 | 104733561 | 104733609 | down | NC_030735.1:104731667-104733609- | 48.2 |
| NC_030725.1 | 48694941 | 48695440 | down | NC_030725.1:48688313-48696526- | 55.4 |
| NC_030729.1 | 52064641 | 52065460 | down | NC_030729.1:52060061-52069161- | 9.550387597 |
| NC_030733.1 | 110462881 | 110463500 | down | NC_030733.1:110455192-110468395- | 50 |
| NC_030725.1 | 92377101 | 92377700 | down | NC_030725.1:92357726-92385188+ | 5.109311741 |
| NC_030734.1 | 104893201 | 104893700 | down | NC_030734.1:104862067-104895568- | 63.9 |
| NC_030724.1 | 177669961 | 177670340 | down | NC_030724.1:177620022-177671619+ | 57.3 |
| NC_030728.1 | 135118701 | 135119340 | down | NC_030728.1:135115660-135120836+ | 165.5 |
| NC_030739.1 | 89937301 | 89937860 | down | NC_030739.1:89931202-89940678- | 7.858585859 |
| NC_030724.1 | 38338561 | 38339120 | down | NC_030724.1:38301752-38362774+ | 44 |
| NC_030734.1 | 15740086 | 15740580 | down | NC_030734.1:15740087-15743233+ | 17.65 |
| NC_030740.1 | 23975741 | 23976300 | down | NC_030740.1:23959774-23977913- | 5.306339904 |
| NC_030727.1 | 56148521 | 56148900 | down | NC_030727.1:56147792-56155050+ | 97.2 |
| NC_030725.1 | 114883481 | 114884240 | down | NC_030725.1:114849418-114909834- | 8.751937984 |
| NC_030726.1 | 151227021 | 151227580 | down | NC_030726.1:151157827-151233081+ | 42.1 |
| NC_030737.1 | 38285601 | 38286200 | down | NC_030737.1:38264622-38344070+ | 6.189873418 |
| NC_030730.1 | 65890261 | 65890800 | down | NC_030730.1:65882281-65891019+ | 115.9 |
| NC_030734.1 | 151252881 | 151253077 | down | NC_030734.1:151250088-151253077- | 47 |
| NC_030726.1 | 38143141 | 38143700 | down | NC_030726.1:38131187-38150843- | 201.3 |
| NC_030734.1 | 108548101 | 108548340 | down | NC_030734.1:108545777-108551795- | 6.453125 |
| NC_030724.1 | 77706706 | 77706800 | down | NC_030724.1:77706707-77765323+ | 63.9 |
| NC_030740.1 | 28290232 | 28290381 | down | NC_030740.1:28289962-28290381- | 47 |
| NC_030740.1 | 34304241 | 34304740 | down | NC_030740.1:34303516-34306452+ | 50.6 |
| NC_030727.1 | 18870821 | 18871280 | down | NC_030727.1:18847003-18886389- | 42.1 |
| NC_030728.1 | 126585189 | 126585300 | down | NC_030728.1:126585190-126635557- | 47 |
| NC_030737.1 | 2752827 | 2752954 | down | NC_030737.1:2752325-2752954+ | 6.607594937 |
| NC_030740.1 | 35052341 | 35052900 | down | NC_030740.1:35038281-35061733- | 40.3 |
| NC_030735.1 | 119272381 | 119273120 | down | NC_030735.1:119271843-119287981+ | 15.4057971 |
| NC_030739.1 | 19273999 | 19274106 | down | NC_030739.1:19272571-19278583+ | 138.9 |
| NC_030728.1 | 32024481 | 32024940 | down | NC_030728.1:32007103-32062625- | 6.061371841 |
| NC_030733.1 | 55997741 | 55998300 | down | NC_030733.1:55988879-56002536- | 43.3 |
| NC_030730.1 | 100091621 | 100092180 | down | NC_030730.1:100052897-100094756+ | 7.069620253 |
| NC_030728.1 | 11270621 | 11271100 | down | NC_030728.1:11267058-11364592- | 47 |
| NC_030736.1 | 78128001 | 78128540 | down | NC_030736.1:78126283-78128899+ | 50.6 |
| NC_030734.1 | 115987441 | 115988020 | down | NC_030734.1:115918223-116005028+ | 7.78125 |
| NC_030733.1 | 27309881 | 27310520 | down | NC_030733.1:27296459-27329399- | 18.82608696 |
| NC_030734.1 | 25777401 | 25777860 | down | NC_030734.1:25711085-25778062+ | 53 |
| NC_030733.1 | 55647221 | 55647980 | down | NC_030733.1:55629942-55679330+ | 4.673387097 |
| NC_030738.1 | 9834101 | 9834660 | down | NC_030738.1:9825166-9841174+ | 47 |
| NC_030733.1 | 51843101 | 51843980 | down | NC_030733.1:51836941-51895818- | 5.735507246 |
| NC_030728.1 | 61204121 | 61204340 | down | NC_030728.1:61185028-61205132- | 44 |
| NC_030740.1 | 61854581 | 61854709 | down | NC_030740.1:61846674-61860440- | 50.6 |
| NC_030728.1 | 53464601 | 53465040 | down | NC_030728.1:53456089-53537641+ | 42.1 |
| NC_030736.1 | 25243021 | 25243560 | down | NC_030736.1:25230969-25244564- | 62.7 |
| NC_030740.1 | 35695301 | 35696100 | down | NC_030740.1:35675311-35714153+ | 6.759493671 |
| NC_030736.1 | 70965641 | 70966120 | down | NC_030736.1:70938042-70985562+ | 47 |
| NC_030741.1 | 70751000 | 70751075 | down | NC_030741.1:70732609-70769249+ | 63.9 |
| NC_030729.1 | 1552461 | 1552840 | down | NC_030729.1:1552451-1553682+ | 53 |
| NC_030733.1 | 35191241 | 35191700 | down | NC_030733.1:35167206-35225123- | 44 |
| NC_030738.1 | 29894590 | 29894685 | down | NC_030738.1:29894591-29924947- | 15.05797101 |
| NC_030725.1 | 164860661 | 164861240 | down | NC_030725.1:164859740-164864200+ | 56.6 |
| NC_030731.1 | 6008661 | 6009100 | down | NC_030731.1:5995387-6016944- | 43.3 |
| NC_030737.1 | 37889781 | 37890320 | down | NC_030737.1:37860497-37906843- | 70 |
| NC_030736.1 | 69647001 | 69647560 | down | NC_030736.1:69632968-69649712+ | 50 |
| NC_030739.1 | 77382321 | 77382520 | down | NC_030739.1:77381642-77386649- | 50.6 |
| NC_030732.1 | 7301961 | 7302500 | down | NC_030732.1:7296019-7314229- | 66.9 |
| NC_030724.1 | 209901181 | 209901560 | down | NC_030724.1:209899351-209905826- | 50.6 |
| NC_030726.1 | 2692941 | 2693500 | down | NC_030726.1:2655999-2707392+ | 47 |
| NC_030738.1 | 113788861 | 113789420 | down | NC_030738.1:113776011-113813536+ | 9.434782609 |
| NC_030733.1 | 48966241 | 48966860 | down | NC_030733.1:48942375-49018351+ | 4.65397351 |
| NC_030741.1 | 79662601 | 79663140 | down | NC_030741.1:79639670-79664137+ | 42.1 |
| NC_030733.1 | 54591061 | 54591620 | down | NC_030733.1:54568365-54645211+ | 14 |
| NC_030732.1 | 35401281 | 35401840 | down | NC_030732.1:35372064-35465281- | 65.1 |
| NC_030735.1 | 24626881 | 24627440 | down | NC_030735.1:24616682-24632726+ | 43.3 |
| NC_030739.1 | 98515221 | 98515780 | down | NC_030739.1:98509968-98520055- | 47 |
| NC_030737.1 | 16949521 | 16949606 | down | NC_030737.1:16947983-16949606- | 55.4 |
| NC_030732.1 | 70461501 | 70462180 | down | NC_030732.1:70449737-70473298- | 4.733944954 |
| NC_030727.1 | 94346661 | 94347180 | down | NC_030727.1:94344340-94352312+ | 55.4 |
| NC_030732.1 | 156047541 | 156048400 | down | NC_030732.1:156022969-156066499+ | 4.038488952 |
| NC_030734.1 | 124381821 | 124382320 | down | NC_030734.1:124380916-124382521+ | 14.325 |
| NC_030733.1 | 135733781 | 135734180 | down | NC_030733.1:135732040-135737941+ | 63.9 |
| NC_030730.1 | 6952501 | 6953080 | down | NC_030730.1:6940032-6973909+ | 10.66666667 |
| NC_030737.1 | 23636841 | 23637220 | down | NC_030737.1:23635211-23646165+ | 65.7 |
| NC_030725.1 | 45753121 | 45753660 | down | NC_030725.1:45709432-45782798+ | 42.1 |
| NC_030741.1 | 79777921 | 79778460 | down | NC_030741.1:79776869-79780799+ | 43.3 |
| NC_030730.1 | 42465081 | 42465620 | down | NC_030730.1:42464694-42469205+ | 17.2 |
| NC_030725.1 | 170330641 | 170331200 | down | NC_030725.1:170311834-170337985+ | 50.6 |
| NC_030733.1 | 14133881 | 14134420 | down | NC_030733.1:14125570-14147570+ | 11.36231884 |
| NC_030728.1 | 30765381 | 30765860 | down | NC_030728.1:30764077-30767125+ | 43.3 |
| NC_030739.1 | 44211901 | 44211964 | down | NC_030739.1:44201241-44211964+ | 94.1 |
| NC_030738.1 | 4100621 | 4101000 | down | NC_030738.1:4094285-4102494- | 47 |
| NC_030732.1 | 78975621 | 78976040 | down | NC_030732.1:78960199-78995968- | 44 |
| NC_030726.1 | 139015041 | 139015580 | down | NC_030726.1:139008053-139021110- | 47 |
| NC_030727.1 | 157840123 | 157840333 | down | NC_030727.1:157839195-157843784- | 104.4 |
| NC_030725.1 | 123667981 | 123668520 | down | NC_030725.1:123636949-123711966+ | 48.2 |
| NC_030741.1 | 79644861 | 79645420 | down | NC_030741.1:79639670-79652845+ | 9.869565217 |
| NC_030726.1 | 7533001 | 7533560 | down | NC_030726.1:7512968-7544722+ | 113.5 |
| NC_030736.1 | 26782544 | 26782568 | down | NC_030736.1:26758675-26786113- | 50.6 |
| NC_030730.1 | 19190741 | 19190854 | down | NC_030730.1:19188557-19194700- | 50.6 |
| NC_030738.1 | 11481761 | 11482340 | down | NC_030738.1:11462943-11533553+ | 150.7021277 |
| NC_030733.1 | 49003301 | 49003860 | down | NC_030733.1:48942375-49018351+ | 62.1 |
| NC_030731.1 | 115186221 | 115187200 | down | NC_030731.1:115185766-115230884+ | 132.3 |
| NC_030724.1 | 215312721 | 215313060 | down | NC_030724.1:215294921-215330846- | 60.3 |
| NC_030735.1 | 119673981 | 119674380 | down | NC_030735.1:119603296-119686801- | 50.6 |
| NC_030740.1 | 35677241 | 35677800 | down | NC_030740.1:35675311-35714153+ | 13.78787879 |
| NC_030728.1 | 19122201 | 19122620 | down | NC_030728.1:19118367-19132156+ | 48.2 |
| NC_030740.1 | 10657821 | 10658360 | down | NC_030740.1:10640082-10671501+ | 53 |
| NC_030726.1 | 19781621 | 19782083 | down | NC_030726.1:19780604-19782083- | 6.8359375 |
| NC_030741.1 | 69165821 | 69165869 | down | NC_030741.1:69165164-69165869+ | 57.3 |
| NC_030734.1 | 94858161 | 94858720 | down | NC_030734.1:94855839-94859689+ | 73.44927536 |
| NC_030728.1 | 134174921 | 134175180 | down | NC_030728.1:134172639-134181223+ | 45.8 |
| NC_030735.1 | 54519301 | 54519840 | down | NC_030735.1:54465428-54535295+ | 53 |
| NC_030732.1 | 149253801 | 149254360 | down | NC_030732.1:149218542-149284904+ | 5.982278481 |
| NC_030727.1 | 102089241 | 102089780 | down | NC_030727.1:102078131-102132237- | 50.6 |
| NC_030725.1 | 88423521 | 88424080 | down | NC_030725.1:88385035-88452229+ | 9.695652174 |
| NC_030736.1 | 67146081 | 67146620 | down | NC_030736.1:67138248-67224186- | 55.4 |
| NC_030736.1 | 97585041 | 97585420 | down | NC_030736.1:97579404-97590100+ | 48.8 |
| NC_030724.1 | 205813581 | 205814080 | down | NC_030724.1:205812321-205816242- | 53 |
| NC_030736.1 | 87752801 | 87753560 | down | NC_030736.1:87751078-87782925+ | 5.687413555 |
| NC_030731.1 | 49097410 | 49097515 | down | NC_030731.1:49094729-49110459- | 44 |
| NC_030740.1 | 15929781 | 15929836 | down | NC_030740.1:15917324-15944574- | 15.075 |
| NC_030729.1 | 47302501 | 47303260 | down | NC_030729.1:47297976-47326091+ | 6.936170213 |
| NC_030728.1 | 96178461 | 96179520 | down | NC_030728.1:96170912-96198335+ | 4.813765182 |
| NC_030730.1 | 112775221 | 112775680 | down | NC_030730.1:112772088-112786917+ | 79.6 |
| NC_030726.1 | 117221861 | 117222380 | down | NC_030726.1:117221803-117236138+ | 60.3 |
| NC_030731.1 | 1349581 | 1349768 | down | NC_030731.1:1347674-1349768+ | 5.285714286 |
| NC_030736.1 | 22999065 | 22999114 | down | NC_030736.1:22983968-23004037- | 5.616352201 |
| NC_030730.1 | 116978821 | 116979520 | down | NC_030730.1:116963359-116995247- | 124.4 |
| NC_030724.1 | 105224801 | 105225180 | down | NC_030724.1:105213124-105234018- | 12.5942029 |
| NC_030735.1 | 89369381 | 89369940 | down | NC_030735.1:89342590-89412284- | 50.6 |
| NC_030738.1 | 5856541 | 5857300 | down | NC_030738.1:5790954-5859660- | 7.555555556 |
| NC_030735.1 | 25754381 | 25754920 | down | NC_030735.1:25754299-25755164+ | 44 |
| NC_030731.1 | 119595621 | 119596140 | down | NC_030731.1:119590190-119598501+ | 77.2 |
| NC_030740.1 | 14334681 | 14335160 | down | NC_030740.1:14329209-14354181+ | 47 |
| NC_030737.1 | 80919331 | 80919640 | down | NC_030737.1:80919332-80919699- | 9.434782609 |
| NC_030725.1 | 79304721 | 79304980 | down | NC_030725.1:79288675-79337801- | 40.3 |
| NC_030730.1 | 28502261 | 28502573 | down | NC_030730.1:28478961-28502573+ | 9.90625 |
| NC_030736.1 | 104805881 | 104806320 | down | NC_030736.1:104734960-104808722- | 50.6 |
| NC_030732.1 | 40157361 | 40157920 | down | NC_030732.1:40120067-40190795- | 10.4765625 |
| NC_030734.1 | 41286081 | 41286980 | down | NC_030734.1:41284645-41288191+ | 4.261728395 |
| NC_030724.1 | 151418981 | 151419480 | down | NC_030724.1:151417117-151424662+ | 7.543778802 |
| NC_030730.1 | 17695179 | 17695300 | down | NC_030730.1:17693266-17695395- | 53 |
| NC_030738.1 | 42173201 | 42173740 | down | NC_030738.1:42122084-42182266- | 56.6 |
| NC_030726.1 | 44470561 | 44471120 | down | NC_030726.1:44463045-44485903- | 62.1 |
| NC_030740.1 | 70809561 | 70809780 | down | NC_030740.1:70799814-70811547- | 42.1 |
| NC_030726.1 | 63633881 | 63634440 | down | NC_030726.1:63633559-63635120- | 63.9 |
| NC_030728.1 | 134149941 | 134150480 | down | NC_030728.1:134149583-134162846+ | 9.434782609 |
| NC_030724.1 | 24753541 | 24754080 | down | NC_030724.1:24748450-24772201+ | 50.6 |
| NC_030741.1 | 26736741 | 26737300 | down | NC_030741.1:26724629-26775531+ | 3995.5 |
| NC_030724.1 | 219423921 | 219424480 | down | NC_030724.1:219400130-219459396+ | 14.38562092 |
| NC_030727.1 | 120671561 | 120672000 | down | NC_030727.1:120670174-120675604+ | 22.925 |
| NC_030734.1 | 148617821 | 148618380 | down | NC_030734.1:148614707-148627999- | 4.692307692 |
| NC_030730.1 | 62626141 | 62626496 | down | NC_030730.1:62623964-62626496- | 43.3 |
| NC_030736.1 | 77448101 | 77448660 | down | NC_030736.1:77432067-77459583- | 44 |
| NC_030736.1 | 108148478 | 108148520 | down | NC_030736.1:108141991-108149645+ | 5.071428571 |
| NC_030733.1 | 68561921 | 68562460 | down | NC_030733.1:68560883-68632668- | 77.2 |
| NC_030726.1 | 145829428 | 145829440 | down | NC_030726.1:145829429-145853876- | 21.725 |
| NC_030732.1 | 31987621 | 31988180 | down | NC_030732.1:31986858-31992151+ | 14.325 |
| NC_030739.1 | 77992221 | 77992660 | down | NC_030739.1:77949377-78011718- | 54.8 |
| NC_030735.1 | 64526281 | 64526660 | down | NC_030735.1:64508561-64533748- | 43.3 |
| NC_030738.1 | 21128321 | 21128540 | down | NC_030738.1:21126033-21131815+ | 59.1 |
| NC_030728.1 | 32007481 | 32007780 | down | NC_030728.1:32007103-32062625- | 50 |
| NC_030728.1 | 57900541 | 57901080 | down | NC_030728.1:57891731-57909513- | 47 |
| NC_030732.1 | 75195921 | 75196300 | down | NC_030732.1:75189264-75200365+ | 73.6 |
| NC_030729.1 | 13189781 | 13190160 | down | NC_030729.1:13163893-13200566- | 53 |
| NC_030736.1 | 39880821 | 39881200 | down | NC_030736.1:39865833-39890228- | 44 |
| NC_030728.1 | 11272141 | 11272660 | down | NC_030728.1:11267058-11364592- | 47 |
| NC_030732.1 | 40161921 | 40162420 | down | NC_030732.1:40120067-40190795- | 53.6 |
| NC_030741.1 | 104412321 | 104413020 | down | NC_030741.1:104322455-104421793- | 8.840425532 |
| NC_030734.1 | 67365421 | 67365960 | down | NC_030734.1:67349394-67379340+ | 6.493670886 |
| NC_030731.1 | 49057961 | 49057990 | down | NC_030731.1:49057756-49057990- | 7.428571429 |
| NC_030725.1 | 67385981 | 67386520 | down | NC_030725.1:67385235-67389817- | 6.359375 |
| NC_030724.1 | 76322943 | 76323025 | down | NC_030724.1:76322345-76323025+ | 48.2 |
| NC_030730.1 | 80240061 | 80240540 | down | NC_030730.1:80237828-80242133- | 40.3 |
| NC_030732.1 | 17768497 | 17768620 | down | NC_030732.1:17760534-17779805- | 54.8 |
| NC_030725.1 | 68166461 | 68166980 | down | NC_030725.1:68165193-68169041- | 9.173913043 |
| NC_030725.1 | 141638241 | 141638620 | down | NC_030725.1:141635960-141639036- | 60.3 |
| NC_030725.1 | 125457361 | 125457820 | down | NC_030725.1:125454381-125463291- | 68.8 |
| NC_030731.1 | 92746701 | 92747332 | down | NC_030731.1:92744631-92747332+ | 4.538251366 |
| NC_030736.1 | 75273001 | 75273400 | down | NC_030736.1:75265993-75308707- | 50.6 |
| NC_030725.1 | 117902301 | 117902700 | down | NC_030725.1:117873200-117918284+ | 50.6 |
| NC_030741.1 | 53271296 | 53271398 | down | NC_030741.1:53259399-53286518- | 50.6 |
| NC_030741.1 | 10240721 | 10241280 | down | NC_030741.1:10229796-10258813- | 40.3 |
| NC_030727.1 | 140979821 | 140980360 | down | NC_030727.1:140962216-141000190+ | 90.5 |
| NC_030733.1 | 85758729 | 85758800 | down | NC_030733.1:85757776-85759096- | 47 |
| NC_030724.1 | 54824479 | 54824603 | down | NC_030724.1:54824480-54841406+ | 50.6 |
| NC_030734.1 | 108579261 | 108579700 | down | NC_030734.1:108568794-108596502+ | 115.4 |
| NC_030729.1 | 72744721 | 72745280 | down | NC_030729.1:72706996-72758563+ | 102 |
| NC_030727.1 | 25152721 | 25153340 | down | NC_030727.1:25144817-25155669- | 10.97468354 |
| NC_030730.1 | 28480141 | 28480740 | down | NC_030730.1:28478961-28502573+ | 21.725 |
| NC_030735.1 | 16028498 | 16028564 | down | NC_030735.1:16028499-16029912+ | 45.2 |
| NC_030724.1 | 94301041 | 94301420 | down | NC_030724.1:94300924-94301765+ | 6.727848101 |
| NC_030727.1 | 58759681 | 58759900 | down | NC_030727.1:58753410-58761615- | 62.7 |
| NC_030734.1 | 85178061 | 85178540 | down | NC_030734.1:85170727-85196178+ | 71.8 |
| NC_030724.1 | 112868181 | 112868400 | down | NC_030724.1:112844854-112877035+ | 45.8 |
| NC_030738.1 | 9846241 | 9846620 | down | NC_030738.1:9840888-9861554+ | 66.3 |
| NC_030734.1 | 144164101 | 144164660 | down | NC_030734.1:144154727-144189623- | 43.3 |
| NC_030735.1 | 94438369 | 94438411 | down | NC_030735.1:94437596-94442623- | 46.26086957 |
| NC_030727.1 | 95075661 | 95076140 | down | NC_030727.1:95075624-95092019+ | 53.6 |
| NC_030724.1 | 170779741 | 170779926 | down | NC_030724.1:170751686-170779926- | 104.4 |
| NC_030734.1 | 129264961 | 129265900 | down | NC_030734.1:129258419-129266660+ | 4.519607843 |
| NC_030737.1 | 21903061 | 21903620 | down | NC_030737.1:21850697-21919060+ | 4.099118943 |
| NC_030734.1 | 72454501 | 72455040 | down | NC_030734.1:72451502-72464681+ | 7.10106383 |
| NC_030738.1 | 5859161 | 5859560 | down | NC_030738.1:5790954-5859660- | 13.63768116 |
| NC_030740.1 | 79488621 | 79489180 | down | NC_030740.1:79448400-79505654- | 55.4 |
| NC_030733.1 | 75880281 | 75880840 | down | NC_030733.1:75870224-75885690- | 44 |
| NC_030729.1 | 8424503 | 8424560 | down | NC_030729.1:8424504-8424688+ | 56.6 |
| NC_030726.1 | 38197441 | 38197980 | down | NC_030726.1:38152920-38227731+ | 63.9 |
| NC_030724.1 | 177369681 | 177370080 | down | NC_030724.1:177353481-177372341+ | 43.3 |
| NC_030738.1 | 25354921 | 25355460 | down | NC_030738.1:25336037-25364511+ | 66.9 |
| NC_030739.1 | 82508121 | 82508162 | down | NC_030739.1:82502240-82508162- | 51.8 |
| NC_030730.1 | 22213723 | 22214160 | down | NC_030730.1:22213724-22223464- | 47 |
| NC_030726.1 | 92571781 | 92572600 | down | NC_030726.1:92559073-92573737- | 47 |
| NC_030728.1 | 24818841 | 24819620 | down | NC_030728.1:24789371-24838647+ | 4.53672788 |
| NC_030734.1 | 116834681 | 116835020 | down | NC_030734.1:116833406-116840355- | 14.775 |
| NC_030727.1 | 157468041 | 157468600 | down | NC_030727.1:157463759-157474652- | 50.6 |
| NC_030728.1 | 74722581 | 74723140 | down | NC_030728.1:74696456-74739829- | 4.664259928 |
| NC_030729.1 | 57721421 | 57721920 | down | NC_030729.1:57692884-57734588+ | 62.1 |
| NC_030738.1 | 22143361 | 22143740 | down | NC_030738.1:22132860-22164554- | 42.1 |
| NC_030731.1 | 72451161 | 72451660 | down | NC_030731.1:72388873-72482319- | 63.9 |
| NC_030733.1 | 49675981 | 49676420 | down | NC_030733.1:49668714-49683622- | 123.2 |
| NC_030737.1 | 44300501 | 44301520 | down | NC_030737.1:44290526-44338283- | 4.997260274 |
| NC_030736.1 | 77229901 | 77230460 | down | NC_030736.1:77218847-77232828+ | 9.695652174 |
| NC_030729.1 | 19416621 | 19417160 | down | NC_030729.1:19413785-19441564+ | 43.3 |
| NC_030726.1 | 64688341 | 64688900 | down | NC_030726.1:64684764-64713466+ | 63.9 |
| NC_030726.1 | 89039901 | 89040460 | down | NC_030726.1:89036276-89040676- | 82 |
| NC_030741.1 | 45722761 | 45723380 | down | NC_030741.1:45718333-45729008- | 61.5 |
| NC_030724.1 | 117843301 | 117843860 | down | NC_030724.1:117830118-117848802+ | 62.1 |
| NC_030727.1 | 41100921 | 41101255 | down | NC_030727.1:41099828-41101255+ | 4.98245614 |
| NC_030739.1 | 66950581 | 66951140 | down | NC_030739.1:66936078-66951585+ | 5.686170213 |
| NC_030736.1 | 110959861 | 110960420 | down | NC_030736.1:110935986-110962311- | 50.6 |
| NC_030735.1 | 26714421 | 26714640 | down | NC_030735.1:26676000-26719112- | 40.3 |
| NC_030731.1 | 40204508 | 40204689 | down | NC_030731.1:40199617-40204689- | 9.198811453 |
| NC_030738.1 | 4259621 | 4260160 | down | NC_030738.1:4198904-4293824- | 47 |
| NC_030736.1 | 102811041 | 102811600 | down | NC_030736.1:102765964-102813100+ | 5.425531915 |
| NC_030738.1 | 74762381 | 74762920 | down | NC_030738.1:74755345-74775455+ | 44 |
| NC_030726.1 | 37288561 | 37289000 | down | NC_030726.1:37283008-37292891+ | 60.3 |
| NC_030731.1 | 15472346 | 15472996 | down | NC_030731.1:15472347-15472996+ | 4.130903899 |
| NC_030738.1 | 117265421 | 117265474 | down | NC_030738.1:117262761-117268068- | 115.9 |
| NC_030740.1 | 61706645 | 61706734 | down | NC_030740.1:61689155-61732394+ | 43.3 |
| NC_030740.1 | 83820661 | 83821200 | down | NC_030740.1:83816718-83825877- | 43.3 |
| NC_030730.1 | 53098061 | 53098520 | down | NC_030730.1:53088235-53100754- | 86.9 |
| NC_030728.1 | 109355901 | 109356460 | down | NC_030728.1:109328761-109359576- | 44 |
| NC_030740.1 | 27449821 | 27450180 | down | NC_030740.1:27443734-27456617+ | 40.3 |
| NC_030730.1 | 122128321 | 122128880 | down | NC_030730.1:122127697-122130702+ | 9.260869565 |
| NC_030741.1 | 98997481 | 98998020 | down | NC_030741.1:98996202-99000849- | 40.3 |
| NC_030733.1 | 32062130 | 32062180 | down | NC_030733.1:32052698-32072375- | 63.3 |
| NC_030734.1 | 76006981 | 76007480 | down | NC_030734.1:75986995-76046521- | 11.79710145 |
| NC_030728.1 | 92358321 | 92358860 | down | NC_030728.1:92348639-92370902- | 50.6 |
| NC_030730.1 | 57735046 | 57735176 | down | NC_030730.1:57733696-57739842- | 47 |
| NC_030738.1 | 6744281 | 6744840 | down | NC_030738.1:6738738-6752450- | 10.14801444 |
| NC_030739.1 | 95805881 | 95806580 | down | NC_030739.1:95791358-95823418- | 4.112318841 |
| NC_030741.1 | 3308859 | 3309120 | down | NC_030741.1:3308860-3309267+ | 4.821176471 |
| NC_030733.1 | 16289981 | 16290540 | down | NC_030733.1:16273438-16292639+ | 44 |
| NC_030739.1 | 89867241 | 89867800 | down | NC_030739.1:89844015-89881717+ | 63.9 |
| NC_030726.1 | 38222321 | 38222880 | down | NC_030726.1:38152920-38227731+ | 18.41860465 |
| NC_030732.1 | 144239731 | 144240180 | down | NC_030732.1:144239732-144240632+ | 4.707581227 |
| NC_030726.1 | 5995021 | 5995360 | down | NC_030726.1:5994533-6000497- | 77.8 |
| NC_030729.1 | 814681 | 815220 | down | NC_030729.1:804965-839132+ | 56.6 |
| NC_030733.1 | 51890841 | 51891320 | down | NC_030733.1:51836941-51895818- | 43.3 |
| NC_030738.1 | 61270121 | 61271040 | down | NC_030738.1:61258245-61272981+ | 6.475570033 |
| NC_030730.1 | 116090281 | 116090820 | down | NC_030730.1:116070276-116091373+ | 17.5 |
| NC_030729.1 | 86841141 | 86841500 | down | NC_030729.1:86839753-86885639+ | 47 |
| NC_030727.1 | 94728501 | 94728720 | down | NC_030727.1:94726141-94730778- | 45.8 |
| NC_030728.1 | 8525161 | 8525720 | down | NC_030728.1:8511201-8533210+ | 70.6 |
| NC_030733.1 | 62404341 | 62404880 | down | NC_030733.1:62377893-62408229+ | 6.7890625 |
| NC_030737.1 | 21909141 | 21909520 | down | NC_030737.1:21850697-21919060+ | 44 |
| NC_030730.1 | 75243241 | 75243800 | down | NC_030730.1:75175064-75245088+ | 77.2 |
| NC_030738.1 | 7088621 | 7089000 | down | NC_030738.1:7077226-7095666- | 10.34883721 |
| NC_030729.1 | 18573201 | 18573960 | down | NC_030729.1:18561325-18581555+ | 12.38383838 |
| NC_030736.1 | 65759201 | 65759740 | down | NC_030736.1:65757828-65776024+ | 44 |
| NC_030728.1 | 32009861 | 32010060 | down | NC_030728.1:32007103-32062625- | 16.72463768 |
| NC_030725.1 | 158596063 | 158596157 | down | NC_030725.1:158593343-158602518- | 43.3 |
| NC_030741.1 | 5354860 | 5355097 | down | NC_030741.1:5354861-5360394+ | 10.66666667 |
| NC_030729.1 | 99462996 | 99463086 | down | NC_030729.1:99459492-99470443- | 9.608695652 |
| NC_030734.1 | 139379002 | 139379420 | down | NC_030734.1:139379003-139389040- | 60.3 |
| NC_030725.1 | 75717770 | 75717860 | down | NC_030725.1:75707855-75718230- | 17.76811594 |
| NC_030733.1 | 100512001 | 100512560 | down | NC_030733.1:100509770-100532936+ | 44 |
| NC_030732.1 | 65494401 | 65494740 | down | NC_030732.1:65437863-65501904- | 9.695652174 |
| NC_030733.1 | 87265581 | 87265960 | down | NC_030733.1:87262924-87289166+ | 47 |
| NC_030724.1 | 111913481 | 111914020 | down | NC_030724.1:111896273-111923058- | 17.5 |
| NC_030732.1 | 55864477 | 55864503 | down | NC_030732.1:55846512-55867689- | 23.37681159 |
| NC_030734.1 | 280521 | 280960 | down | NC_030734.1:278279-300750- | 57.3 |
| NC_030724.1 | 142916761 | 142916829 | down | NC_030724.1:142915956-142916829- | 122 |
| NC_030731.1 | 40199616 | 40199763 | down | NC_030731.1:40199617-40204689- | 4.421146953 |
| NC_030734.1 | 1962821 | 1963260 | down | NC_030734.1:1953722-1966791+ | 47 |
| NC_030734.1 | 99003901 | 99004440 | down | NC_030734.1:98965296-99020496- | 63.9 |
| NC_030734.1 | 94878321 | 94878880 | down | NC_030734.1:94874285-94907137- | 7.721518987 |
| NC_030735.1 | 105852356 | 105852720 | down | NC_030735.1:105852357-105883555- | 90.5 |
| NC_030740.1 | 3593041 | 3593139 | down | NC_030740.1:3590189-3593139- | 47 |
| NC_030740.1 | 74735721 | 74736160 | down | NC_030740.1:74728135-74744784+ | 47 |
| NC_030732.1 | 40508961 | 40509600 | down | NC_030732.1:40487710-40517909+ | 17.68115942 |
| NC_030730.1 | 64427601 | 64427616 | down | NC_030730.1:64398215-64430267- | 95.4 |
| NC_030725.1 | 123658021 | 123658440 | down | NC_030725.1:123613284-123664846- | 6.496124031 |
| NC_030726.1 | 63205141 | 63205520 | down | NC_030726.1:63188409-63228806- | 6.131782946 |
| NC_030735.1 | 1239181 | 1239620 | down | NC_030735.1:1235972-1249038+ | 9.260869565 |
| NC_030730.1 | 56061121 | 56061660 | down | NC_030730.1:56045603-56074142- | 81.4 |
| NC_030732.1 | 19214721 | 19215560 | down | NC_030732.1:19214115-19219212+ | 5.518595041 |
| NC_030738.1 | 104698241 | 104698640 | down | NC_030738.1:104692550-104701830- | 60.3 |
| NC_030733.1 | 100449661 | 100450400 | down | NC_030733.1:100439304-100454719- | 9.457364341 |
| NC_030733.1 | 55663741 | 55664300 | down | NC_030733.1:55629942-55679330+ | 9.319148936 |
| NC_030738.1 | 6746541 | 6747260 | down | NC_030738.1:6738738-6752450- | 5.240229885 |
| NC_030735.1 | 82697481 | 82697740 | down | NC_030735.1:82696522-82708561- | 7.434343434 |
| NC_030741.1 | 90635521 | 90635900 | down | NC_030741.1:90629737-90642238+ | 45.8 |
| NC_030727.1 | 123201981 | 123202440 | down | NC_030727.1:123187633-123240783- | 53 |
| NC_030728.1 | 16988656 | 16989180 | down | NC_030728.1:16988657-16996742- | 12.3164557 |
| NC_030727.1 | 81009357 | 81009860 | down | NC_030727.1:81009358-81016995+ | 43.3 |
| NC_030726.1 | 38111281 | 38111820 | down | NC_030726.1:38104223-38115262- | 50.6 |
| NC_030737.1 | 40172862 | 40173360 | down | NC_030737.1:40172863-40173473- | 60.3 |
| NC_030735.1 | 39751741 | 39752280 | down | NC_030735.1:39715393-39773623- | 50.6 |
| NC_030731.1 | 26462021 | 26462520 | down | NC_030731.1:26414920-26464888+ | 19.24637681 |
| NC_030732.1 | 56814001 | 56814780 | down | NC_030732.1:56764774-56815592+ | 6.651898734 |
| NC_030728.1 | 67266141 | 67266700 | down | NC_030728.1:67254216-67270711- | 42.1 |
| NC_030726.1 | 2710641 | 2711200 | down | NC_030726.1:2658327-2722058+ | 40.3 |
| NC_030725.1 | 157872441 | 157873000 | down | NC_030725.1:157852488-157936090- | 40.3 |
| NC_030725.1 | 114881721 | 114882680 | down | NC_030725.1:114849418-114909834- | 10.9453125 |
| NC_030735.1 | 12321761 | 12322140 | down | NC_030735.1:12303709-12343610+ | 47 |
| NC_030727.1 | 49511681 | 49512240 | down | NC_030727.1:49503950-49539388- | 40.3 |
| NC_030735.1 | 22252461 | 22252495 | down | NC_030735.1:22207853-22258519- | 77.8 |
| NC_030730.1 | 97541501 | 97542060 | down | NC_030730.1:97537498-97588328- | 40.475 |
| NC_030728.1 | 126013201 | 126013760 | down | NC_030728.1:125999976-126027134+ | 6.6015625 |
| NC_030729.1 | 86038341 | 86038880 | down | NC_030729.1:86036225-86077810- | 40.3 |
| NC_030734.1 | 92154901 | 92155460 | down | NC_030734.1:92153509-92158089- | 63.9 |
| NC_030730.1 | 48984461 | 48984880 | down | NC_030730.1:48977836-48997611+ | 14.45 |
| NC_030730.1 | 88262801 | 88263360 | down | NC_030730.1:88253032-88267464+ | 48.2 |
| NC_030730.1 | 120608921 | 120609480 | down | NC_030730.1:120606068-120609950- | 14.52173913 |
| NC_030724.1 | 117380701 | 117381200 | down | NC_030724.1:117333458-117401717+ | 50.6 |
| NC_030727.1 | 82795381 | 82795920 | down | NC_030727.1:82794818-82828287- | 44 |
| NC_030736.1 | 117076781 | 117077180 | down | NC_030736.1:117050506-117080090- | 53 |
| NC_030725.1 | 123669861 | 123670440 | down | NC_030725.1:123636949-123711966+ | 7.737373737 |
| NC_030727.1 | 55448341 | 55448740 | down | NC_030727.1:55400728-55456104- | 6 |
| NC_030728.1 | 94727521 | 94728220 | down | NC_030728.1:94699134-94742730- | 8.222222222 |
| NC_030724.1 | 215297521 | 215297960 | down | NC_030724.1:215294921-215330846- | 43.3 |
| NC_030733.1 | 56245121 | 56245480 | down | NC_030733.1:56183365-56250785+ | 15.975 |
| NC_030730.1 | 80694541 | 80695020 | down | NC_030730.1:80693697-80695646- | 48.8 |
| NC_030731.1 | 13467141 | 13467700 | down | NC_030731.1:13465955-13487196+ | 77.2 |
| NC_030733.1 | 54612881 | 54613420 | down | NC_030733.1:54568365-54645211+ | 53 |
| NC_030726.1 | 107261561 | 107262100 | down | NC_030726.1:107252335-107263026- | 5.012145749 |
| NC_030730.1 | 58277641 | 58278600 | down | NC_030730.1:58259653-58295936- | 4.333333333 |
| NC_030741.1 | 33005261 | 33005720 | down | NC_030741.1:33001086-33007662- | 80.3 |
| NC_030734.1 | 85381241 | 85381565 | down | NC_030734.1:85373943-85381565+ | 85.7 |
| NC_030728.1 | 42738381 | 42738780 | down | NC_030728.1:42732008-42748572+ | 40.3 |
| NC_030736.1 | 42195381 | 42195940 | down | NC_030736.1:42193927-42196835- | 15.56521739 |
| NC_030738.1 | 106808601 | 106809140 | down | NC_030738.1:106783446-106827778- | 50.6 |
| NC_030732.1 | 105473561 | 105475100 | down | NC_030732.1:105467083-105486317- | 7.287449393 |
| NC_030734.1 | 58481161 | 58481720 | down | NC_030734.1:58474629-58485760+ | 5.078175896 |
| NC_030740.1 | 84633281 | 84633820 | down | NC_030740.1:84632052-84639705+ | 96.6 |
| NC_030724.1 | 99231175 | 99231262 | down | NC_030724.1:99227261-99233398- | 77.2 |
| NC_030724.1 | 92540361 | 92540860 | down | NC_030724.1:92521854-92541472+ | 9.260869565 |
| NC_030728.1 | 17163161 | 17163700 | down | NC_030728.1:17139602-17184083+ | 77.8 |
| NC_030735.1 | 26439721 | 26440640 | down | NC_030735.1:26435576-26480600- | 91.7 |
| NC_030741.1 | 99394687 | 99394700 | down | NC_030741.1:99394688-99394997+ | 43.3 |
| NC_030728.1 | 66277 | 66320 | down | NC_030728.1:66278-66496- | 7.975708502 |
| NC_030736.1 | 62528981 | 62529540 | down | NC_030736.1:62519217-62530694+ | 47 |
| NC_030734.1 | 14655861 | 14656400 | down | NC_030734.1:14654464-14662440- | 43.3 |
| NC_030730.1 | 127266641 | 127266840 | down | NC_030730.1:127257238-127274734+ | 53.6 |
| NC_030727.1 | 90341961 | 90342700 | down | NC_030727.1:90313971-90349569+ | 79.6 |
| NC_030736.1 | 87802321 | 87802616 | down | NC_030736.1:87759542-87802616- | 8.483028721 |
| NC_030730.1 | 97563581 | 97564140 | down | NC_030730.1:97537498-97588328- | 42.1 |
| NC_030735.1 | 77028481 | 77029040 | down | NC_030735.1:77010646-77031371+ | 7.259493671 |
| NC_030737.1 | 67347261 | 67347820 | down | NC_030737.1:67330074-67378218- | 13.46376812 |
| NC_030729.1 | 73831321 | 73832020 | down | NC_030729.1:73822446-73857325+ | 4.919354839 |
| NC_030727.1 | 90522901 | 90523640 | down | NC_030727.1:90493354-90554773+ | 14.15 |
| NC_030728.1 | 26442141 | 26442520 | down | NC_030728.1:26435784-26456070- | 47 |
| NC_030738.1 | 80425221 | 80425760 | down | NC_030738.1:80366594-80437257- | 56.6 |
| NC_030725.1 | 116636681 | 116637220 | down | NC_030725.1:116629986-116668178- | 91.7 |
| NC_030731.1 | 12607261 | 12607820 | down | NC_030731.1:12580263-12609893+ | 12.15942029 |
| NC_030724.1 | 178901701 | 178902400 | down | NC_030724.1:178875686-178969784- | 9.875968992 |
| NC_030729.1 | 47861661 | 47862280 | down | NC_030729.1:47847561-47867214- | 5.614754098 |
| NC_030733.1 | 72779401 | 72779960 | down | NC_030733.1:72778498-72808177+ | 5.904255319 |
| NC_030725.1 | 92612521 | 92613320 | down | NC_030725.1:92611294-92615999- | 5.845744681 |
| NC_030734.1 | 132698781 | 132698800 | down | NC_030734.1:132698603-132701453- | 45.2 |
| NC_030732.1 | 53183201 | 53183540 | down | NC_030732.1:53180171-53187152- | 4.08496732 |
| NC_030724.1 | 95328281 | 95328840 | down | NC_030724.1:95303144-95334046+ | 60.3 |
| NC_030738.1 | 103675481 | 103675860 | down | NC_030738.1:103664816-103676431- | 54.8 |
| NC_030733.1 | 20419141 | 20419520 | down | NC_030733.1:20381730-20435294- | 50.6 |
| NC_030724.1 | 95243121 | 95243580 | down | NC_030724.1:95237531-95249314+ | 25.66666667 |
| NC_030728.1 | 830941 | 831460 | down | NC_030728.1:829782-836135+ | 44 |
| NC_030737.1 | 4089781 | 4090280 | down | NC_030737.1:4084890-4114972+ | 53.6 |
| NC_030731.1 | 29601281 | 29601829 | down | NC_030731.1:29572410-29601829+ | 48.8 |
| NC_030728.1 | 93421361 | 93421920 | down | NC_030728.1:93400860-93446683+ | 16.725 |
| NC_030733.1 | 73841301 | 73842060 | down | NC_030733.1:73816968-73855795- | 12.68115942 |
| NC_030734.1 | 60359201 | 60359640 | down | NC_030734.1:60356514-60361474- | 61.5 |
| NC_030728.1 | 126243711 | 126243828 | down | NC_030728.1:126241500-126243828+ | 10.84057971 |
| NC_030735.1 | 24620281 | 24620880 | down | NC_030735.1:24616682-24632726+ | 14.52173913 |
| NC_030728.1 | 106555201 | 106555760 | down | NC_030728.1:106532429-106558332+ | 50.6 |
| NC_030739.1 | 56021881 | 56022220 | down | NC_030739.1:56009911-56031741+ | 44 |
| NC_030732.1 | 65452341 | 65452780 | down | NC_030732.1:65436341-65488605- | 44 |
| NC_030736.1 | 123916661 | 123917120 | down | NC_030736.1:123913827-123928310+ | 58.5 |
| NC_030726.1 | 149208041 | 149208500 | down | NC_030726.1:149195795-149212177+ | 42.1 |
| NC_030738.1 | 88013981 | 88014520 | down | NC_030738.1:87956897-88055456- | 73.6 |
| NC_030740.1 | 785641 | 786020 | down | NC_030740.1:743487-787000+ | 60.3 |
| NC_030738.1 | 23804421 | 23804740 | down | NC_030738.1:23802016-23807338+ | 79.1 |
| NC_030724.1 | 178638301 | 178638860 | down | NC_030724.1:178634907-178659015- | 44 |
| NC_030740.1 | 18984141 | 18984680 | down | NC_030740.1:18970340-18997734+ | 48.8 |
| NC_030735.1 | 40923941 | 40924500 | down | NC_030735.1:40859694-40940435- | 48.8 |
| NC_030735.1 | 51092361 | 51093000 | down | NC_030735.1:51054847-51111738- | 5.206422018 |
| NC_030731.1 | 101279181 | 101279980 | down | NC_030731.1:101273617-101281630- | 7.14556962 |
| NC_030740.1 | 10663861 | 10664320 | down | NC_030740.1:10640082-10671501+ | 6.7890625 |
| NC_030730.1 | 95371105 | 95371160 | down | NC_030730.1:95371106-95381040+ | 47 |
| NC_030730.1 | 116188901 | 116189240 | down | NC_030730.1:116186166-116190473- | 44 |
| NC_030735.1 | 20300841 | 20301400 | down | NC_030735.1:20292006-20332812- | 57.3 |
| NC_030736.1 | 42239181 | 42239740 | down | NC_030736.1:42219245-42241192- | 90.5 |
| NC_030728.1 | 114393881 | 114394440 | down | NC_030728.1:114389671-114396038- | 77.2 |
| NC_030726.1 | 28124461 | 28125020 | down | NC_030726.1:28119397-28134931- | 4.927797834 |
| NC_030738.1 | 5790461 | 5791000 | down | NC_030738.1:5763818-5810717- | 13.63768116 |
| NC_030725.1 | 157443401 | 157443940 | down | NC_030725.1:157441552-157458896+ | 62.1 |
| NC_030729.1 | 86186741 | 86187340 | down | NC_030729.1:86185532-86209222+ | 5.654255319 |
| NC_030738.1 | 1410261 | 1410800 | down | NC_030738.1:1344503-1437839- | 44 |
| NC_030737.1 | 40184141 | 40184227 | down | NC_030737.1:40177916-40184227+ | 70 |
| NC_030731.1 | 6622301 | 6622840 | down | NC_030731.1:6621296-6648172+ | 89.9 |
| NC_030732.1 | 111738841 | 111739220 | down | NC_030732.1:111722289-111758786+ | 9.260869565 |
| NC_030732.1 | 96108941 | 96109500 | down | NC_030732.1:96103376-96118377- | 77.2 |
| NC_030730.1 | 101179111 | 101179180 | down | NC_030730.1:101179112-101179828+ | 60.3 |
| NC_030738.1 | 113793941 | 113794580 | down | NC_030738.1:113776011-113813536+ | 5.894736842 |
| NC_030727.1 | 103015601 | 103015840 | down | NC_030727.1:103012594-103021306- | 43.3 |
| NC_030727.1 | 55308381 | 55308760 | down | NC_030727.1:55308024-55317716+ | 62.7 |
| NC_030732.1 | 62691921 | 62692440 | down | NC_030732.1:62672221-62727059+ | 42.1 |
| NC_030741.1 | 34417727 | 34417831 | down | NC_030741.1:34415884-34417831- | 44 |
| NC_030726.1 | 67867601 | 67868140 | down | NC_030726.1:67846403-67879835- | 60.3 |
| NC_030726.1 | 158189261 | 158189820 | down | NC_030726.1:158180215-158224268- | 5.817813765 |
| NC_030729.1 | 97092041 | 97092580 | down | NC_030729.1:97084385-97102561- | 6.496124031 |
| NC_030729.1 | 25179381 | 25179920 | down | NC_030729.1:25178879-25197609+ | 6.113360324 |
| NC_030724.1 | 62187441 | 62187860 | down | NC_030724.1:62161716-62226589+ | 57.3 |
| NC_030740.1 | 37548481 | 37549180 | down | NC_030740.1:37547646-37552854+ | 6.476534296 |
| NC_030735.1 | 89391521 | 89392080 | down | NC_030735.1:89342590-89412284- | 51.8 |
| NC_030730.1 | 71872501 | 71872900 | down | NC_030730.1:71870565-71876335+ | 47 |
| NC_030741.1 | 38044763 | 38044916 | down | NC_030741.1:38044764-38044916- | 4.306451613 |
| NC_030738.1 | 26239501 | 26239960 | down | NC_030738.1:26233646-26264128+ | 50 |
| NC_030741.1 | 74273146 | 74273247 | down | NC_030741.1:74267099-74273247+ | 15.03594771 |
| NC_030738.1 | 10338516 | 10338700 | down | NC_030738.1:10338517-10348751+ | 9.608695652 |
| NC_030725.1 | 56532157 | 56532265 | down | NC_030725.1:56526705-56540203+ | 53.6 |
| NC_030730.1 | 112946921 | 112947300 | down | NC_030730.1:112940464-112954211- | 99 |
| NC_030728.1 | 19439761 | 19440300 | down | NC_030728.1:19435791-19443982- | 47 |
| NC_030732.1 | 150551501 | 150552060 | down | NC_030732.1:150528647-150578749- | 42.1 |
| NC_030734.1 | 129433401 | 129433840 | down | NC_030734.1:129407662-129481427- | 8.426356589 |
| NC_030727.1 | 72025801 | 72026500 | down | NC_030727.1:71984875-72041779- | 66.9 |
| NC_030734.1 | 72019681 | 72020020 | down | NC_030734.1:71978005-72042575- | 14.325 |
| NC_030730.1 | 28376281 | 28376780 | down | NC_030730.1:28370802-28412523+ | 43.3 |
| NC_030739.1 | 96386201 | 96386920 | down | NC_030739.1:96379137-96387192- | 6.092165899 |
| NC_030737.1 | 4862661 | 4863220 | down | NC_030737.1:4861389-4879629+ | 63.3 |
| NC_030726.1 | 98031421 | 98031807 | down | NC_030726.1:97998007-98031807+ | 43.3 |
| NC_030730.1 | 23330421 | 23330800 | down | NC_030730.1:23324938-23363272+ | 57.3 |
| NC_030724.1 | 117844441 | 117844840 | down | NC_030724.1:117830118-117848802+ | 50.6 |
| NC_030736.1 | 25741841 | 25742380 | down | NC_030736.1:25739814-25747374+ | 72.3 |
| NC_030733.1 | 134167481 | 134168300 | down | NC_030733.1:134138537-134179828- | 9.34375 |
| NC_030726.1 | 53106158 | 53106293 | down | NC_030726.1:53097737-53106293- | 60.3 |
| NC_030730.1 | 65614901 | 65614930 | down | NC_030730.1:65612978-65616247- | 50.6 |
| NC_030724.1 | 127443881 | 127444260 | down | NC_030724.1:127441538-127447143+ | 6.7890625 |
| NC_030738.1 | 111201981 | 111202478 | down | NC_030738.1:111195418-111202478+ | 42.1 |
| NC_030728.1 | 106723281 | 106723700 | down | NC_030728.1:106705589-106729257+ | 47 |
| NC_030735.1 | 86651221 | 86652040 | down | NC_030735.1:86643280-86657983+ | 4.697580645 |
| NC_030725.1 | 32818881 | 32819280 | down | NC_030725.1:32816746-32824857- | 55.4 |
| NC_030725.1 | 60504341 | 60504900 | down | NC_030725.1:60502623-60508315+ | 70.6 |
| NC_030731.1 | 49018201 | 49018600 | down | NC_030731.1:49015909-49043515+ | 48.2 |
| NC_030724.1 | 155495821 | 155496300 | down | NC_030724.1:155464464-155499720+ | 44 |
| NC_030725.1 | 71162681 | 71163220 | down | NC_030725.1:71159154-71179397+ | 76 |
| NC_030741.1 | 26299976 | 26300080 | down | NC_030741.1:26299977-26300409- | 55.4 |
| NC_030736.1 | 73490201 | 73491220 | down | NC_030736.1:73480300-73494827- | 47 |
| NC_030734.1 | 149136621 | 149137180 | down | NC_030734.1:149111445-149140082+ | 66.3 |
| NC_030734.1 | 25774321 | 25774700 | down | NC_030734.1:25711085-25778062+ | 68.8 |
| NC_030738.1 | 106922941 | 106923500 | down | NC_030738.1:106920412-106927081- | 60.3 |
| NC_030737.1 | 3714221 | 3714760 | down | NC_030737.1:3707843-3738590- | 179.5 |
| NC_030736.1 | 51302641 | 51303100 | down | NC_030736.1:51247395-51344846- | 42.1 |
| NC_030730.1 | 144057461 | 144058440 | down | NC_030730.1:144019758-144071521+ | 8.240310078 |
| NC_030739.1 | 79633161 | 79633660 | down | NC_030739.1:79633025-79637269- | 44 |
| NC_030732.1 | 97334581 | 97335140 | down | NC_030732.1:97286623-97345147+ | 6.21875 |
| NC_030730.1 | 74976021 | 74976400 | down | NC_030730.1:74974786-74976432+ | 9.223404255 |
| NC_030734.1 | 108694811 | 108694940 | down | NC_030734.1:108694812-108695403+ | 55.4 |
| NC_030732.1 | 89417281 | 89417660 | down | NC_030732.1:89415715-89435674+ | 55.4 |
| NC_030732.1 | 9552481 | 9552860 | down | NC_030732.1:9551538-9591606- | 6.2734375 |
| NC_030739.1 | 36898461 | 36898860 | down | NC_030739.1:36878783-36932595- | 50.6 |
| NC_030735.1 | 109179141 | 109179680 | down | NC_030735.1:109177839-109193009- | 5.769230769 |
| NC_030727.1 | 73866341 | 73866720 | down | NC_030727.1:73866342-73867917- | 56.6 |
| NC_030738.1 | 11470161 | 11470700 | down | NC_030738.1:11462943-11533553+ | 50.6 |
| NC_030740.1 | 89412521 | 89412740 | down | NC_030740.1:89395635-89414192- | 57.3 |
| NC_030735.1 | 106520901 | 106521440 | down | NC_030735.1:106463895-106539403+ | 85.1 |
| NC_030731.1 | 61123501 | 61124040 | down | NC_030731.1:61117816-61139683- | 9.141414141 |
| NC_030724.1 | 24603521 | 24603683 | down | NC_030724.1:24597643-24603683- | 40.3 |
| NC_030724.1 | 113649322 | 113649360 | down | NC_030724.1:113646911-113650656+ | 63.9 |
| NC_030724.1 | 45050141 | 45050680 | down | NC_030724.1:45029589-45051252+ | 42.1 |
| NC_030732.1 | 50708921 | 50709480 | down | NC_030732.1:50672751-50716322+ | 19.9 |
| NC_030738.1 | 68828901 | 68829300 | down | NC_030738.1:68826078-68836226- | 53 |
| NC_030739.1 | 81200781 | 81201160 | down | NC_030739.1:81195195-81204486+ | 41.5 |
| NC_030740.1 | 71563501 | 71563760 | down | NC_030740.1:71542285-71563779- | 79.6 |
| NC_030738.1 | 6487615 | 6487980 | down | NC_030738.1:6487616-6494254- | 43.3 |
| NC_030734.1 | 111967261 | 111967800 | down | NC_030734.1:111965120-111974522- | 53 |
| NC_030736.1 | 87802041 | 87802260 | down | NC_030736.1:87759542-87802616- | 8.67721519 |
| NC_030738.1 | 116951961 | 116952380 | down | NC_030738.1:116950683-116953045+ | 4.26185567 |
| NC_030729.1 | 52987081 | 52987700 | down | NC_030729.1:52980304-53048108+ | 5.132978723 |
| NC_030740.1 | 5516961 | 5517440 | down | NC_030740.1:5516068-5518657+ | 48.2 |
| NC_030724.1 | 66700521 | 66700626 | down | NC_030724.1:66700441-66729015+ | 45.8 |
| NC_030735.1 | 24588841 | 24589380 | down | NC_030735.1:24582902-24591722+ | 50.6 |
| NC_030735.1 | 35167301 | 35167780 | down | NC_030735.1:35166287-35169493+ | 63.3 |
| NC_030738.1 | 20291721 | 20292040 | down | NC_030738.1:20289265-20295282- | 57.3 |
| NC_030728.1 | 60057561 | 60057940 | down | NC_030728.1:60012289-60064952- | 77.2 |
| NC_030725.1 | 71088061 | 71088620 | down | NC_030725.1:71055458-71089817+ | 43.3 |
| NC_030730.1 | 103284561 | 103285120 | down | NC_030730.1:103279413-103290638- | 47 |
| NC_030738.1 | 113805381 | 113805820 | down | NC_030738.1:113776011-113813536+ | 50.6 |
| NC_030732.1 | 65458621 | 65459180 | down | NC_030732.1:65436341-65488605- | 40.3 |
| NC_030737.1 | 37258801 | 37259360 | down | NC_030737.1:37248833-37329967- | 21.425 |
| NC_030739.1 | 87286221 | 87286780 | down | NC_030739.1:87268043-87301184- | 14.95652174 |
| NC_030737.1 | 31937881 | 31938180 | down | NC_030737.1:31865134-31952348- | 45.8 |
| NC_030728.1 | 43602181 | 43602580 | down | NC_030728.1:43589751-43610080+ | 14.325 |
| NC_030737.1 | 80746541 | 80747140 | down | NC_030737.1:80739320-80749740+ | 60.3 |
| NC_030733.1 | 87331121 | 87331500 | down | NC_030733.1:87327918-87344392+ | 50.6 |
| NC_030726.1 | 10010161 | 10010660 | down | NC_030726.1:9962038-10015841- | 55.4 |
| NC_030738.1 | 105007021 | 105007420 | down | NC_030738.1:105006607-105012229- | 15.975 |
| NC_030738.1 | 11826681 | 11827220 | down | NC_030738.1:11807499-11839059+ | 6.16194332 |
| NC_030737.1 | 86595356 | 86595449 | down | NC_030737.1:86595357-86595746- | 50 |
| NC_030739.1 | 81401421 | 81401940 | down | NC_030739.1:81400770-81402301- | 22.625 |
| NC_030740.1 | 23337741 | 23338300 | down | NC_030740.1:23319315-23345504- | 56.6 |
| NC_030733.1 | 27921201 | 27921500 | down | NC_030733.1:27915473-27922505+ | 43.3 |
| NC_030729.1 | 22805081 | 22805620 | down | NC_030729.1:22802389-22856039- | 14.15 |
| NC_030729.1 | 52066161 | 52066620 | down | NC_030729.1:52060061-52069161- | 12.15942029 |
| NC_030732.1 | 70453141 | 70453580 | down | NC_030732.1:70449737-70473298- | 14.15 |
| NC_030724.1 | 199282361 | 199282900 | down | NC_030724.1:199274419-199308721- | 50.6 |
| NC_030737.1 | 21866401 | 21866960 | down | NC_030737.1:21850697-21919060+ | 40.3 |
| NC_030734.1 | 130774441 | 130774940 | down | NC_030734.1:130757249-130810923- | 51.8 |
| NC_030724.1 | 151240821 | 151241040 | down | NC_030724.1:151240750-151243118- | 7.813953488 |
| NC_030740.1 | 26241621 | 26242560 | down | NC_030740.1:26193657-26246000+ | 4.083860759 |
| NC_030739.1 | 26465001 | 26465540 | down | NC_030739.1:26452785-26466904- | 15.671875 |
| NC_030728.1 | 4471561 | 4471800 | down | NC_030728.1:4438839-4473875- | 6.340206186 |
| NC_030738.1 | 87405561 | 87405956 | down | NC_030738.1:87401471-87405956+ | 45.2 |
| NC_030733.1 | 13327561 | 13327760 | down | NC_030733.1:13320759-13334120- | 7.494949495 |
| NC_030730.1 | 94923721 | 94924100 | down | NC_030730.1:94918034-94938489- | 8.898989899 |
| NC_030741.1 | 53274321 | 53274384 | down | NC_030741.1:53259399-53286518- | 44 |
| NC_030726.1 | 26933941 | 26934320 | down | NC_030726.1:26876493-26954046- | 59.1 |
| NC_030730.1 | 75445421 | 75445640 | down | NC_030730.1:75415038-75495235- | 77.2 |
| NC_030734.1 | 106652981 | 106653303 | down | NC_030734.1:106644563-106653303+ | 44 |
| NC_030735.1 | 26714921 | 26715220 | down | NC_030735.1:26676000-26719112- | 40.3 |
| NC_030738.1 | 42175341 | 42175920 | down | NC_030738.1:42122084-42182266- | 9.695652174 |
| NC_030736.1 | 52088521 | 52088960 | down | NC_030736.1:52034286-52133494- | 55.4 |
| NC_030724.1 | 7163761 | 7164300 | down | NC_030724.1:7148842-7190782- | 4.755760369 |
| NC_030739.1 | 48716881 | 48717420 | down | NC_030739.1:48685824-48739989+ | 9.695652174 |
| NC_030734.1 | 49672321 | 49672560 | down | NC_030734.1:49655887-49688000- | 4.359477124 |
| NC_030727.1 | 58454421 | 58454880 | down | NC_030727.1:58444248-58477195- | 63.9 |
| NC_030724.1 | 88700481 | 88701020 | down | NC_030724.1:88688709-88712164+ | 50.6 |
| NC_030731.1 | 9878701 | 9878872 | down | NC_030731.1:9855606-9878872- | 11.79710145 |
| NC_030734.1 | 40976701 | 40976960 | down | NC_030734.1:40950570-40985891- | 46.3 |
| NC_030727.1 | 85988481 | 85989000 | down | NC_030727.1:85970055-86003833+ | 50.6 |
| NC_030724.1 | 141722981 | 141723340 | down | NC_030724.1:141715643-141740686- | 42.1 |
| NC_030737.1 | 46725561 | 46725940 | down | NC_030737.1:46724188-46743135- | 50.6 |
| NC_030724.1 | 152774701 | 152776080 | down | NC_030724.1:152754234-152779042+ | 135.9 |
| NC_030730.1 | 58192781 | 58192795 | down | NC_030730.1:58190365-58210707+ | 7.555555556 |
| NC_030729.1 | 84809821 | 84810120 | down | NC_030729.1:84809156-84810465- | 51.8 |
| NC_030733.1 | 113085461 | 113086020 | down | NC_030733.1:113026628-113092151+ | 5.465408805 |
| NC_030731.1 | 43087481 | 43088040 | down | NC_030731.1:43076943-43112840+ | 63.9 |
| NC_030727.1 | 87325081 | 87325460 | down | NC_030727.1:87321955-87328696+ | 47 |
| NC_030732.1 | 40661188 | 40661311 | down | NC_030732.1:40658102-40667177- | 81.4 |
| NC_030731.1 | 7063466 | 7063569 | down | NC_030731.1:7059412-7070535- | 79.6 |
| NC_030732.1 | 9554421 | 9554980 | down | NC_030732.1:9551538-9591606- | 4.026785714 |
| NC_030724.1 | 151920081 | 151920520 | down | NC_030724.1:151916198-151925943+ | 14.15 |
| NC_030730.1 | 112680961 | 112681160 | down | NC_030730.1:112658344-112684621+ | 70 |
| NC_030724.1 | 36499481 | 36500040 | down | NC_030724.1:36479171-36506385+ | 50.6 |
| NC_030725.1 | 119652301 | 119652900 | down | NC_030725.1:119647136-119653344- | 7.2578125 |
| NC_030728.1 | 129289821 | 129290200 | down | NC_030728.1:129271277-129293691+ | 63.9 |
| NC_030732.1 | 112453561 | 112454040 | down | NC_030732.1:112452100-112483281- | 56.6 |
| NC_030733.1 | 27680461 | 27681000 | down | NC_030733.1:27677076-27695790+ | 77.2 |
| NC_030727.1 | 49509041 | 49509660 | down | NC_030727.1:49503950-49539388- | 9.608695652 |
| NC_030740.1 | 33251461 | 33251820 | down | NC_030740.1:33241736-33252497+ | 41.5 |
| NC_030734.1 | 109950621 | 109951160 | down | NC_030734.1:109926070-110019550- | 47 |
| NC_030736.1 | 32259341 | 32260440 | down | NC_030736.1:32259040-32260672- | 10.5106383 |
| NC_030724.1 | 133763481 | 133763980 | down | NC_030724.1:133707130-133771900+ | 57.3 |
| NC_030724.1 | 68500281 | 68500920 | down | NC_030724.1:68488476-68512398+ | 12.85443038 |
| NC_030726.1 | 133930781 | 133931320 | down | NC_030726.1:133928623-133949475+ | 14.15 |
| NC_030741.1 | 40489241 | 40489800 | down | NC_030741.1:40475716-40500241+ | 30.95 |
| NC_030728.1 | 139704362 | 139704460 | down | NC_030728.1:139704363-139706088+ | 4.307971014 |
| NC_030731.1 | 95561641 | 95562420 | down | NC_030731.1:95559361-95563805- | 6.457364341 |
| NC_030724.1 | 83840521 | 83841540 | down | NC_030724.1:83828634-83844198- | 165 |
| NC_030728.1 | 19540921 | 19541480 | down | NC_030728.1:19532331-19576644- | 16.725 |
| NC_030734.1 | 49670741 | 49671460 | down | NC_030734.1:49655887-49688000- | 4.97107438 |
| NC_030727.1 | 47394661 | 47395020 | down | NC_030727.1:47394598-47396007- | 8.945736434 |
| NC_030729.1 | 59373461 | 59373840 | down | NC_030729.1:59341935-59382514- | 80.3 |
| NC_030724.1 | 5083061 | 5083440 | down | NC_030724.1:5082022-5084230+ | 11.27536232 |
| NC_030727.1 | 51515761 | 51516320 | down | NC_030727.1:51451779-51524723+ | 50.6 |
| NC_030734.1 | 106573141 | 106573660 | down | NC_030734.1:106570294-106592066+ | 51.8 |
| NC_030725.1 | 117579321 | 117579880 | down | NC_030725.1:117519727-117586436- | 8.414141414 |
| NC_030726.1 | 92910441 | 92911000 | down | NC_030726.1:92897631-92912991+ | 45.2 |
| NC_030738.1 | 28397981 | 28398089 | down | NC_030738.1:28396873-28398089+ | 85.7 |
| NC_030734.1 | 71983061 | 71983280 | down | NC_030734.1:71968902-71997437- | 42.1 |
| NC_030736.1 | 124912741 | 124913440 | down | NC_030736.1:124911525-124978289+ | 15.075 |
| NC_030740.1 | 103915281 | 103915880 | down | NC_030740.1:103910403-103929517+ | 4.273282443 |
| NC_030724.1 | 21778581 | 21779100 | down | NC_030724.1:21759382-21781690- | 40.3 |
| NC_030738.1 | 82267081 | 82268020 | down | NC_030738.1:82226834-82278380- | 6.359375 |
| NC_030728.1 | 50287561 | 50288040 | down | NC_030728.1:50269778-50297257- | 77.2 |
| NC_030726.1 | 47146861 | 47147300 | down | NC_030726.1:47139888-47148374+ | 10.23188406 |
| NC_030724.1 | 64345681 | 64346220 | down | NC_030724.1:64293221-64360872- | 50.6 |
| NC_030735.1 | 45879261 | 45879460 | down | NC_030735.1:45818010-45898775+ | 83.9 |
| NC_030731.1 | 40367690 | 40367920 | down | NC_030731.1:40367691-40376291- | 4.283526487 |
| NC_030728.1 | 142560061 | 142560720 | down | NC_030728.1:142552653-142570230- | 5.138297872 |
| NC_030729.1 | 113607201 | 113607720 | down | NC_030729.1:113606433-113617975- | 50 |
| NC_030741.1 | 5555294 | 5555436 | down | NC_030741.1:5544637-5556960+ | 45.2 |
| NC_030732.1 | 149241901 | 149242460 | down | NC_030732.1:149218542-149284904+ | 77.2 |
| NC_030741.1 | 90432261 | 90432820 | down | NC_030741.1:90429828-90443295- | 43.3 |
| NC_030738.1 | 1962361 | 1962920 | down | NC_030738.1:1959074-1964076- | 5.654255319 |
| NC_030731.1 | 64456601 | 64456800 | down | NC_030731.1:64452542-64456810- | 105.1 |
| NC_030725.1 | 88434321 | 88434880 | down | NC_030725.1:88385035-88452229+ | 8.253164557 |
| NC_030728.1 | 64999261 | 64999640 | down | NC_030728.1:64942543-65025535- | 6.310077519 |
| NC_030734.1 | 55170821 | 55171240 | down | NC_030734.1:55170101-55172665+ | 43.3 |
| NC_030740.1 | 37078051 | 37078238 | down | NC_030740.1:37062039-37078915- | 14.925 |
| NC_030735.1 | 17242861 | 17243240 | down | NC_030735.1:17200470-17283574- | 57.3 |
| NC_030730.1 | 65340641 | 65341200 | down | NC_030730.1:65304172-65363549- | 48.2 |
| NC_030739.1 | 95801401 | 95801960 | down | NC_030739.1:95791358-95823418- | 4.797833935 |
| NC_030739.1 | 36923341 | 36923900 | down | NC_030739.1:36878783-36932595- | 6.354666667 |
| NC_030739.1 | 13211441 | 13211980 | down | NC_030739.1:13211149-13213788- | 58.5 |
| NC_030741.1 | 1390261 | 1390740 | down | NC_030741.1:1389355-1417562- | 48.8 |
| NC_030727.1 | 74527541 | 74527980 | down | NC_030727.1:74517468-74531572- | 50.6 |
| NC_030735.1 | 115901816 | 115901870 | down | NC_030735.1:115850931-115913631+ | 72.4 |
| NC_030739.1 | 89948976 | 89949280 | down | NC_030739.1:89948977-89950609+ | 6.808510638 |
| NC_030736.1 | 50144001 | 50144480 | down | NC_030736.1:50139623-50188849- | 7.919191919 |
| NC_030728.1 | 126610621 | 126611420 | down | NC_030728.1:126585190-126635557- | 94.1 |
| NC_030726.1 | 8391781 | 8392340 | down | NC_030726.1:8385549-8396453+ | 8.426356589 |
| NC_030725.1 | 111176461 | 111177100 | down | NC_030725.1:111170995-111180088+ | 11.10144928 |
| NC_030725.1 | 39873041 | 39873119 | down | NC_030725.1:39867870-39876958- | 45.8 |
| NC_030729.1 | 12808361 | 12808900 | down | NC_030729.1:12803645-12815184+ | 48.2 |
| NC_030735.1 | 127835361 | 127835413 | down | NC_030735.1:127835249-127843134- | 60.3 |
| NC_030735.1 | 89601121 | 89601660 | down | NC_030735.1:89583720-89602905- | 7.858585859 |
| NC_030735.1 | 96663581 | 96664140 | down | NC_030735.1:96603145-96671190+ | 44 |
| NC_030725.1 | 124797781 | 124798160 | down | NC_030725.1:124792644-124889820- | 80.3 |
| NC_030724.1 | 83828961 | 83829420 | down | NC_030724.1:83828634-83844198- | 7.0234375 |
| NC_030740.1 | 103576341 | 103576364 | down | NC_030740.1:103572214-103577482+ | 50.6 |
| NC_030724.1 | 30145961 | 30146340 | down | NC_030724.1:30102543-30175557- | 60.3 |
| NC_030734.1 | 70774061 | 70774620 | down | NC_030734.1:70762612-70819895- | 4.604166667 |
| NC_030735.1 | 32173541 | 32174080 | down | NC_030735.1:32157927-32175267+ | 40.3 |
| NC_030737.1 | 21903681 | 21904220 | down | NC_030737.1:21850697-21919060+ | 76.6 |
| NC_030734.1 | 45980501 | 45980880 | down | NC_030734.1:45946484-46031765- | 10.23188406 |
| NC_030728.1 | 7632461 | 7632840 | down | NC_030728.1:7628695-7711913- | 13.85 |
| NC_030729.1 | 96878781 | 96879280 | down | NC_030729.1:96873774-96881356+ | 40.3 |
| NC_030731.1 | 18225741 | 18226300 | down | NC_030731.1:18169009-18244923+ | 5.9566787 |
| NC_030729.1 | 18442481 | 18442940 | down | NC_030729.1:18414494-18453627- | 40.3 |
| NC_030727.1 | 35611501 | 35611880 | down | NC_030727.1:35597679-35618311- | 44 |
| NC_030740.1 | 66555381 | 66555600 | down | NC_030740.1:66547118-66560595- | 45.2 |
| NC_030725.1 | 39668841 | 39669320 | down | NC_030725.1:39647328-39746555+ | 50.6 |
| NC_030730.1 | 75420261 | 75420540 | down | NC_030730.1:75415038-75495235- | 50.6 |
| NC_030733.1 | 76866879 | 76867004 | down | NC_030733.1:76815120-76867004+ | 4.013029316 |
| NC_030724.1 | 119834881 | 119835360 | down | NC_030724.1:119831308-119840530- | 42.1 |
| NC_030734.1 | 115547061 | 115547360 | down | NC_030734.1:115541716-115549149- | 27.625 |
| NC_030737.1 | 21920221 | 21920560 | down | NC_030737.1:21918707-21970718+ | 43.3 |
| NC_030724.1 | 106175541 | 106175940 | down | NC_030724.1:106172989-106227305- | 4.71659919 |
| NC_030730.1 | 96291981 | 96292500 | down | NC_030730.1:96286137-96293016- | 47 |
| NC_030734.1 | 50309981 | 50310860 | down | NC_030734.1:50290680-50318543- | 11.53623188 |
| NC_030734.1 | 40854561 | 40854960 | down | NC_030734.1:40801049-40876523+ | 43.3 |
| NC_030728.1 | 69146121 | 69146500 | down | NC_030728.1:69075891-69152944- | 47 |
| NC_030732.1 | 105471901 | 105472420 | down | NC_030732.1:105467083-105486317- | 86.9 |
| NC_030736.1 | 22507961 | 22508520 | down | NC_030736.1:22499685-22522247- | 53.6 |
| NC_030734.1 | 110001921 | 110002460 | down | NC_030734.1:109926070-110019550- | 67.5 |
| NC_030732.1 | 54866441 | 54867000 | down | NC_030732.1:54840367-54869231- | 12.50724638 |
| NC_030737.1 | 85072452 | 85072575 | down | NC_030737.1:85072453-85073007- | 65.1 |
| NC_030734.1 | 67266101 | 67266360 | down | NC_030734.1:67235050-67286740+ | 16.425 |
| NC_030730.1 | 134723381 | 134723920 | down | NC_030730.1:134708333-134735516- | 50.6 |
| NC_030729.1 | 2681386 | 2681560 | down | NC_030729.1:2681387-2690832- | 56.6 |
| NC_030734.1 | 8378001 | 8378540 | down | NC_030734.1:8313019-8380616+ | 55.4 |
| NC_030738.1 | 22171861 | 22172560 | down | NC_030738.1:22132860-22178509- | 9.403100775 |
| NC_030727.1 | 34273781 | 34274320 | down | NC_030727.1:34257312-34283962+ | 50.6 |
| NC_030725.1 | 8964141 | 8964700 | down | NC_030725.1:8929446-8966866- | 50.6 |
| NC_030740.1 | 107475981 | 107476400 | down | NC_030740.1:107474472-107483075- | 53.6 |
| NC_030731.1 | 61126321 | 61126940 | down | NC_030731.1:61117816-61139683- | 6.744680851 |
| NC_030728.1 | 46450681 | 46451420 | down | NC_030728.1:46429558-46452954+ | 80.3 |
| NC_030731.1 | 40374801 | 40376291 | down | NC_030731.1:40367691-40376291- | 4.368980123 |
| NC_030733.1 | 113130841 | 113131380 | down | NC_030733.1:113086872-113155941+ | 6.2734375 |
| NC_030735.1 | 26452261 | 26452800 | down | NC_030735.1:26435576-26480600- | 10.66666667 |
| NC_030725.1 | 124848821 | 124849880 | down | NC_030725.1:124792644-124889820- | 4.219844358 |
| NC_030735.1 | 40867121 | 40867680 | down | NC_030735.1:40859694-40940435- | 43.3 |
| NC_030724.1 | 49168861 | 49169400 | down | NC_030724.1:49151630-49202844+ | 60.3 |
| NC_030731.1 | 32305101 | 32305480 | down | NC_030731.1:32281404-32308020+ | 57.3 |
| NC_030740.1 | 106544581 | 106544820 | down | NC_030740.1:106530809-106551812- | 60.9 |
| NC_030724.1 | 210632312 | 210632440 | down | NC_030724.1:210632313-210632640+ | 14.08695652 |
| NC_030728.1 | 67314321 | 67314840 | down | NC_030728.1:67312475-67318246- | 15.075 |
| NC_030724.1 | 125055861 | 125056320 | down | NC_030724.1:125035768-125060840+ | 4.14379085 |
| NC_030724.1 | 99236073 | 99236161 | down | NC_030724.1:99234967-99244277- | 4.438967136 |
| NC_030741.1 | 26729301 | 26729680 | down | NC_030741.1:26724629-26775531+ | 9.971014493 |
| NC_030727.1 | 14012599 | 14012640 | down | NC_030727.1:14012600-14012831- | 19.5959596 |
| NC_030728.1 | 106724461 | 106725540 | down | NC_030728.1:106705589-106729257+ | 8.414141414 |
| NC_030729.1 | 107399601 | 107400160 | down | NC_030729.1:107327670-107404496- | 50.6 |
| NC_030728.1 | 24307521 | 24307838 | down | NC_030728.1:24272004-24307838+ | 65.1 |
| NC_030732.1 | 156066041 | 156066499 | down | NC_030732.1:156022969-156066499+ | 4.180722892 |
| NC_030731.1 | 44440221 | 44440780 | down | NC_030731.1:44438446-44462377- | 53.6 |
| NC_030725.1 | 172163586 | 172163822 | down | NC_030725.1:172163587-172163822- | 6.361702128 |
| NC_030740.1 | 8468741 | 8469280 | down | NC_030740.1:8466111-8474820+ | 50.6 |
| NC_030736.1 | 51323201 | 51324080 | down | NC_030736.1:51247395-51344846- | 6.516129032 |
| NC_030740.1 | 1011761 | 1012100 | down | NC_030740.1:1004020-1015147- | 14.325 |
| NC_030725.1 | 52029561 | 52030120 | down | NC_030725.1:52011672-52032281+ | 50.6 |
| NC_030737.1 | 85497061 | 85497460 | down | NC_030737.1:85491206-85500653- | 10.67553191 |
| NC_030734.1 | 106222301 | 106222840 | down | NC_030734.1:106218704-106230213- | 62.1 |
| NC_030736.1 | 51498061 | 51498280 | down | NC_030736.1:51469385-51543210- | 48.8 |
| NC_030726.1 | 29553841 | 29554860 | down | NC_030726.1:29538309-29558007+ | 66.3 |
| NC_030730.1 | 106340561 | 106341120 | down | NC_030730.1:106334735-106350572- | 20.82608696 |
| NC_030732.1 | 123262121 | 123262720 | down | NC_030732.1:123244426-123276657+ | 7.676767677 |
| NC_030738.1 | 6951661 | 6952220 | down | NC_030738.1:6937442-6965380+ | 95.4 |
| NC_030738.1 | 21801681 | 21802120 | down | NC_030738.1:21799549-21805965- | 93.6 |
| NC_030727.1 | 39317386 | 39317400 | down | NC_030727.1:39313369-39318775- | 4.325434439 |
| NC_030725.1 | 55248127 | 55248244 | down | NC_030725.1:55228932-55249969- | 50.6 |
| NC_030734.1 | 106553981 | 106554580 | down | NC_030734.1:106551618-106571778+ | 61.5 |
| NC_030728.1 | 133182365 | 133182400 | down | NC_030728.1:133178398-133184234+ | 40.3 |
| NC_030732.1 | 13528241 | 13528700 | down | NC_030732.1:13488859-13572457- | 55.4 |

txStart/txEnd: Start/end position of the differentially methylated RNA peak.

**Table S2** Exhibit a significant change in both m^6^A level and circRNA expression in AZ-exposed *X. laevis* compared to the controls

|  |  | Differentlly methylated m^6^A sites | | | | | Differentially expressed circRNAs | | |
| --- | --- | --- | --- | --- | --- | --- | --- | --- | --- |
| circRNA | chrom | txStart | txEnd | Foldchange | P_value | Regulation | Foldchange | P_value | Regulation |
| NC_030733.1:68560883-68632668- | NC_030733.1 | 68600261 | 68600820 | 60.4 | 4.95285E-05 | up | 3.5860167 | 0.043969985 | down |
| NC_030737.1:31865134-31952348- | NC_030737.1 | 31937501 | 31938020 | 5.819354839 | 0.000545014 | up | 2.4008951 | 0.043632488 | down |
| NC_030737.1:83574596-83583489+ | NC_030737.1 | 83581621 | 83582140 | 10.9137931 | 0.000633668 | up | 2.51604 | 0.010475416 | down |
| NC_030733.1:34483227-34511810- | NC_030733.1 | 34499801 | 34500060 | 15.96103896 | 5.26742E-07 | up | 2.9830953 | 0.021171355 | down |
| NC_030733.1:68560883-68632668- | NC_030733.1 | 68598961 | 68599960 | 4.30859375 | 1.65526E-06 | up | 3.5860167 | 0.043969985 | down |
| NC_030737.1:31865134-31952348- | NC_030737.1 | 31865133 | 31865180 | 51.4 | 0.000185095 | up | 2.4008951 | 0.043632488 | down |
| NC_030733.1:68560883-68632668- | NC_030733.1 | 68561921 | 68562460 | 77.2 | 4.27704E-06 | down | 3.5860167 | 0.043969985 | down |
| NC_030738.1:21126033-21131815+ | NC_030738.1 | 21128321 | 21128540 | 59.1 | 5.99372E-05 | down | 3.0045334 | 0.015214659 | up |
| NC_030737.1:31865134-31952348- | NC_030737.1 | 31937881 | 31938180 | 45.8 | 0.000426193 | down | 2.4008951 | 0.043632488 | down |

txStart/txEnd: Start/end position of the differentially methylated RNA sites.
